# Intravenous vitamin C monotherapy in critically ill patients: a systematic review and meta-analysis of randomized controlled trials with trial sequential analysis

**DOI:** 10.1186/s13613-023-01116-x

**Published:** 2023-03-07

**Authors:** Zheng-Yii Lee, Luis Ortiz-Reyes, Charles Chin Han Lew, M. Shahnaz Hasan, Lu Ke, Jayshil J. Patel, Christian Stoppe, Daren K. Heyland

**Affiliations:** 1 Department of Anaesthesiology, Faculty of Medicine, University of Malaya, 50603 Kuala Lumpur, Malaysia; 2 Clinical Evaluation Research Unit, Department of Critical Care Medicine, Queen’s University, Kingston, ON K7L 3N6 Canada; 3 Department of Dietetics & Nutrition, Ng Teng Fong General Hospital, 1 Jurong East Street 21, Singapore, 609606 Singapore; 4 Department of Critical Care Medicine, Jinling Hospital, Medical School of Nanjing University, No. 305 Zhongshan East Road, Nanjing, 210000 Jiangsu China; 5 National Institute of Healthcare Data Science, Nanjing University, Nanjing, China; 6 Division of Pulmonary & Critical Care Medicine, Medical College of Wisconsin, Milwaukee, WI USA; 7 Department of Anesthesiology, Intensive Care, Emergency and Pain Medicine, University Hospital Würzburg, Würzburg, Germany; 8 Department of Cardiac Anesthesiology and Intensive Care Medicine, Charité Berlin, Berlin, Germany

**Keywords:** Vitamin C, Ascorbic acids, Sepsis, Critical illness, Systematic review

## Abstract

**Background:**

A recent landmark randomized controlled trial (RCT) in septic patients demonstrated an increased risk of death and persistent organ dysfunction with intravenous Vitamin C (IVVC) monotherapy, which represents a disparate result from previous systematic reviews and meta-analyses (SRMA). We performed an updated SRMA of IVVC monotherapy to summarize and explore heterogeneity across current trials and conduct trial sequential analysis (TSA) to guard against type-I or type-II statistical errors.

**Methods:**

RCTs evaluating IVVC in adult critically ill patients were included. Four databases were searched from inception to 22 June 2022 without language restrictions. The primary outcome was overall mortality. Random effect meta-analysis was performed to estimate the pooled risk ratio. TSA for mortality was performed using the DerSimonian–Laird random effect model, alpha 5%, beta 10%, and relative risk reduction (RRR) of 30%, 25%, and 20%.

**Results:**

We included 16 RCTs (*n* = 2130). IVVC monotherapy is associated with significant reduction in overall mortality [risk ratio (RR) 0.73, 95% confidence interval (CI) 0.60–0.89; *p* = 0.002; *I*^2^ = 42%]. This finding is supported by TSA using RRR of 30% and 25%, and sensitivity analysis using fixed-effect meta-analysis. However, the certainty of our mortality finding was rated low using GRADE due to the serious risk of bias and inconsistency. In a priori subgroup analyses, we found no differences between single vs multicenter, higher (≥ 10,000 mg/day) vs lower dose and sepsis vs non-sepsis trials. *Post-hoc*, we found no differences in subgroup analysis of earlier (< 24 h) vs delayed treatment, longer (> 4 days) vs shorter treatment duration, and low vs other risk of bias studies. IVVC may have the greatest benefit in trials that enrolled patients above (i.e., > 37.5%; RR 0.65, 95% CI 0.54–0.79) vs below (i.e., ≤ 37.5%; RR 0.89, 95% CI 0.68–1.16) median control group mortality (test for subgroup differences: *p* = 0.06), and TSA supported this.

**Conclusions:**

IVVC monotherapy may be associated with mortality benefits in critically ill patients, particularly in patients with a high risk of dying. Given the low certainty of evidence, this potentially life-saving therapy warrants further studies to identify the optimal timing, dosage, treatment duration, and patient population that will benefit most from IVVC monotherapy.

*PROSPERO Registration ID*: CRD42022323880. Registered 7th May 2022.

**Supplementary Information:**

The online version contains supplementary material available at 10.1186/s13613-023-01116-x.

## Introduction

Vitamin C is an essential micronutrient with pleiotropic functions and can serve as an antioxidant [1]. In critically ill patients, vitamin C level is depleted [2, 3] despite receipt of enteral and/or parenteral nutrition sources [4] and lower levels have been associated with worse clinical outcomes [3, 4].

In the last decade, metabolic resuscitation using high-dose intravenous vitamin C (IVVC) has been tested as a pharmacotherapeutic agent to modify the inflammatory cascade and improve clinical outcomes in critical illness [5]. Most trials have evaluated high-dose IVVC monotherapy or in combination with intravenous hydrocortisone and thiamine [5]. Previously, several systematic reviews and meta-analyses (SRMAs) of randomized controlled trials (RCTs) were performed to evaluate the effects of IVVC in critical illness [6–9]. Overall, no significant effect of IVVC on mortality was found. However, in a subgroup analysis, we previously demonstrated that IVVC monotherapy confers a greater benefit [relative risk (RR) 0.64, 95% confidence interval (CI) 0.49 to 0.83; *p* = 0.0006], compared to combination therapy (RR 1.00, 95% CI 0.85 to 1.18; *p* = 0.99), and the test for subgroup differences was significant (*p* = 0.004) [8, 10], further enhance the signal of mortality benefit of IVVC monotherapy.

The Lessening Organ Dysfunction With VITamin C (LOVIT) study, a recent large, multicenter trial compared high-dose (200 mg/kg/day body weight for 96 h) IVVC monotherapy to placebo in patients with septic shock found IVVC monotherapy increased the risk of a composite endpoint of death or persistent organ dysfunction at day 28 (RR 1.21, 95% CI 1.05 to 1.40; *p* = 0.01) [11]. However, an SRMA that included the results of LOVIT (published at the same time) found that IVVC monotherapy was still associated with a significant mortality benefit (RR 0.65, 95% CI 0.50–0.86; *p* = 0.002), compared to combination therapy (RR 0.94, 95% CI 0.74–1.19; *p* = 0.58), and there was still evidence for a significant test for subgroup differences (*p* = 0.04) [12]. However, this SRMA included several studies with inappropriate study design or patient population: a quasi-trial [13], a study that excluded intubated patients [14], and a study that was published in abstract form [15], which may limit the confidence of the reported results.

The disparate findings from SRMAs of RCTs and a well-conducted landmark RCT of IVVC monotherapy are difficult to reconcile. It is possible that type-1 errors may have occurred in the IVVC monotherapy subgroup analyses of previous SRMAs, because each SRMA update entails repeated statistical testing which would increase the risk of random error. A statistical technique, Trial Sequential Analysis (TSA), that is analogous to interim analyses in RCTs can be employed to control for type-I error, because it sets a more stringent statistical significance threshold for the initial trials and gradually reduces the threshold with each subsequent trial [16]. Another shortfall of previous SRMAs is that certain important effect modifiers are not robustly explored [12]. Therefore, we aim to perform an updated SRMA of RCTs specifically in critically ill patients and include TSA to robustly examine if the effects of IVVC monotherapy are modified by dose, timing, treatment duration, the included patient population, or the trial quality.

## Methodology

We conducted this SRMA according to the PRISMA 2020 guidelines [17]. The PRISMA 2020 checklist is shown in the Additional file 1. The study protocol was registered in PROSPERO (CRD42022323880).

### Eligibility criteria

RCTs among critically ill patients comparing IVVC monotherapy with placebo or usual care and reported at least one clinical outcome (mortality, infectious complications, duration of mechanical ventilation, and intensive care unit [ICU] or hospital length of stay) were included. Quasi-randomized trials, studies conducted among elective surgical or non-critically ill patients, studies that used any combination therapy, and studies published in abstract form were excluded.

### Information source and search strategies

MEDLINE, EMBASE, CENTRAL and CINAHL were searched with relevant subject headings and keywords from database inception to 22 June 2022 without language restrictions. Personal files and the reference list of previous SRMAs were reviewed. Additional file 1: Table S1 shows the search strategies.

### Study selection process

One author screened the title and abstract for potential eligible studies (ZYL). The potential studies were retrieved, and the full text were evaluated independently by two authors (ZYL, LOR). Disagreements were discussed with the senior author (DKH).

### Data collection process and data items

Data items were collected independently by two authors (ZYL, LOR) in a standardized data abstraction form (Additional file 1). Two studies published in Chinese were abstracted by a single author that understands Chinese (ZYL) [18, 19]. Where needed, authors were contacted (up to two times) to obtain additional data.

### Study quality and risk-of-bias assessment

The quality of the included trials was evaluated independently by two authors (ZYL, LOR) using the Canadian Critical Care Nutrition (CCN) Methodological Quality System [8], and the Cochrane Risk of Bias version 2 (ROB2) [20]. The use of CCN Methodological Quality System allow us to compare critical care nutrition trials across time and topics. Any disagreements were resolved by the senior author (DKH).

### Statistical analysis and data handling

The primary outcome was overall mortality. The mortality timepoint was chosen in the following order: 28-day mortality, hospital mortality, ICU mortality, other mortality—if a study reported multiple mortality timepoints. Secondary outcomes were: 28-day mortality, longer term mortality (≥ 60 days and the longest follow-up reported), duration of mechanical ventilation, ICU and hospital length of stay, incidences of acute kidney injury and renal replacement therapy, SOFA score at 96 h, dose and duration of vasopressor used, and adverse events (as reported by the original manuscript).

Dichotomous outcomes were presented as risk ratio (RR), while continuous outcomes were presented as mean difference (MD) or standardized mean difference (SMD). The DerSimonian–Laird random-effect model was used to account for the different patients’ characteristics, dosing, duration, and starting time of the IVVC [21]. For the analysis of continuous outcome, authors of the primary publication were contacted to obtain the mean and standard deviation (SD) if this information was not reported. Median and range are not converted to mean and SD for meta-analysis.

The following subgroup analyses were planned a priori: single vs multicenter center trial, higher (administering ≥ 10,000 mg/day based on a 70 kg adult) vs lower dose of vitamin C [8, 22], and sepsis vs non-sepsis trial.

For studies that had > 1 group of IVVC intervention with different dosages, the results of the two IVVC group were combined and compared with the control group. For subgroup analysis of higher vs lower doses of Vitamin C, the control group sample size was distributed proportionally to the two intervention groups [23]. For subgroup analysis of ≤  vs > 24 h, it is assumed that the intervention was initiated > 24 h for 4 studies with unclear reporting of this information [24–27].

*Post-hoc*—since LOVIT was a negative trial, which is in the opposite direction from other trials, [28] we performed a sensitivity analysis for our primary outcomes using the fixed-effect model (Mantel–Haenszel method), because this model assigns more weight to larger trials [28]. We also performed a sensitivity analysis in trials, where the patients’ mean, or median baseline vitamin C level was reported to be deficit (≤ 21 μmol/L [22]). For subgroup analysis, we added six additional post-hoc analyses to further explore the source of heterogeneity in included trials: studies that enrolled patients above vs below the median overall control group mortality, >  vs ≤ median CCN score, low vs other risk of bias, duration of treatment > or ≤ 4 days, commencement of the intervention ≤  vs > 24 h of an event, and bolus vs continuous infusion.

Heterogeneity was quantified by the *I*^2^ measure. Publication bias was visualized by the funnel plot. Egger's test was conducted for meta-analyses that included > 10 studies [29], using STATA 16.1 (StataCorp LLC, Texas). All meta-analysis and test for subgroup differences were conducted using Revman 5.4 (Cochrane IMS, Oxford, UK). A two-sided *p* value of < 0.05 was considered statistically significant, and a *p* value of < 0.10 was considered a trend [30].

### Trial sequential analysis

To control for type-I [31] and type-II errors and further confirming the results of our meta-analysis, TSA for overall mortality was performed with the following parameters [32]: alpha 5%, beta 10%, and the DerSimonian–Laird random effect model. Between-trial heterogeneity was adjusted by the diversity-estimate (*D*^2^). The control group mortality was the observed mortality in this current meta-analysis (i.e., 35%), and the effect size [relative risk reduction (RRR)] of 30% was used based on the subgroup analysis of IVVC monotherapy in previous meta-analyses [8, 12], with sensitivity analyses for RRR of 25% and 20%. We also performed a sensitivity analysis using the Biggerstaff–Tweedie (BT) random-effect model as it attributes more weight to larger trials and less weight to smaller trials [16]. All TSA was performed using the TSA software (0.9.5.10 Beta, The Copenhagen Trial Unit, Denmark). (See Additional file 1 for detailed TSA explanation),

#### Certainty of the evidence

The Grading of Recommendations Assessment, Development, and Evaluation (GRADE) system was used to rate the certainty of evidence for only the primary outcome [33]. Secondary outcomes were not evaluated, since most of the continuous outcomes were reported as medians (e.g., length of stay and SOFA score) and could not be statistically aggregated or were reported only in a few studies [e.g., incidences of acute kidney injury (AKI) or renal replacement therapy (RRT)]. The quality of the evidence was rated as high, moderate, low, and very low by considering the risk of bias, inconsistency, indirectness, imprecision, and publication bias. GRADEpro was used to prepare the GRADE evidence profile table.

## Results

### Study selection

A total of 1361 records were found, and 66 full texts were assessed after the removal of duplicates and title/abstract screening. In addition, 451 articles were screened from citation searching and personal files, and 30 were retrieved. Therefore, a total of 96 articles were assessed for full-text. Finally, 16 RCTs (*n* = 2130) were included (Additional file 1: Figure S1) [11, 18, 19, 24–27, 34–42]. Additional file 1: Table S2 lists the excluded studies.

### Study characteristics

The study characteristics are summarized in Table 1. Ten (62.5%) studies enrolled patients with sepsis, and seven (43.8%) were multicenter trials. The mean/median age ranged from 29.4 to 73, where 11 (68.8%) of the studies had a mean/median age of > 55 years. Ten studies reported the acute physiology and chronic health evaluation II (APACHE II) score (range: 13.5–24.5), and 11 studies reported the sequential organ failure assessment (SOFA) score (range: 5.9–13.3). Seven studies reported baseline plasma vitamin C levels (range: 3.4–129.3 μmol/L) in which patients in six studies were considered vitamin C deficient (range: 10–22 μmol/L) [11, 19, 27, 36, 41], one study had a very borderline vitamin C level (median: 22 μmol/L) and we also considered the patients were deficit in vitamin C [38], and one study had normal baseline vitamin C levels [26].

**Table 1:** Study and baseline characteristics

Author, year (Country)	Population	Number of centers	*n* (Analyzed)	Age	M/F	APACHE II	SOFA	Baseline Vit C level (umol/L)	Vit C level after intervention (umol/L)	Weight in kg
1. Ferron-Celma 2008 (Spain)	Abdominal surgery with postop mortality risk > 30%	1	20	67.8 ± 4.5 vs 65.1 ± 3.6	11/9	NR	NR	NR	NR	NR
2. Razmkon 2011 (Iran)	Age ≥ 16, severe head injury (GCS ≤ 8). Exclude: G6PD, severe liver or renal disease	2	76 (Group A, B and D)	31.1 (16–67) vs 29.5 (19–75) vs 29.4 (16–68)	63/13	NR	NR	NR	NR	NR
3. Fowler 2014 (USA)	Severe sepsis with organ dysfunction	1	24	30–70 vs 49–92 vs 54–68	13/11	20.4 (12–23) vs 24.0 (12–33) vs 20.4 (15–29)	10.1 ± 2.0 vs 10.8 ± 4.4 vs 13.3 ± 2.9	All: 17.9 ± 2.4 20.2 (11–45) vs 16.7 (14–28) vs 17.0 (11–50)	Day 4: 331 (110–806) vs 3082 (1592–5722) vs 15.6 (7–27)	NR
4. Zabet 2016 (Iran)	Surgical ICU patients with septic shock requiring vasopressors	1	28	64.14 ± 15.98 vs 63.71 ± 13.84	21/7	19.07 ± 5.18 vs 23 ± 5.61	11.78 ± 2.22 vs 12.35 ± 3.00	NR	NR	NR
5. Chen 2019 (China)	Age ≥ 18, sepsis, ICU stay ≥ 96 h Exclude: G6PD, severe arrhythmia	2	122	69 ± 13.5 vs 67.4 ± 17.3 vs 72 ± 16.6	88/31	18.5 ± 5.1 vs 19.6 ± 6.0 vs 18.1 ± 5.7	6.3 ± 2.5 vs 7.0 ± 2.7 vs 6.3 ± 2.8	3.5 ± 2.0 vs 4.0 ± 3.2 vs 3.4 ± 2.5	48 h: 26.65 ± 18.94 vs 48.88 ± 31.43 vs 2.80 ± 2.91 96 h: 52.40 ± 56.03 vs 111.62 ± 64 vs 4.26 ± 3.22	64.8 ± 7.0 vs 65.5 ± 7.6 vs 65.3 ± 6.2
6. Fowler 2019 (USA)	Age ≥ 18, sepsis, mechanically ventilated with PF ratio < 300. Exclude: active kidney stone, DKA, interstitial lung disease	7	166	53 (39–67) vs 57 (44–70)	90/77	NR	mSOFA: 9.8 ± 3.2 vs 10.3 ± 3.1	22 (8–39) vs 22 (11–37)	48 h: 166 (88–376) vs 23 (9–37) 96 h: 169 (87–412) vs 26 (9–41) 168 h: 46 (19–66) vs 29 (12–39)	NR
7. Niu 2019 (China)	Age 18–75, sepsis in ICU. Exclude: immunocompromised, metastatic cancer	1	234	58.2 ± 14.1 ± 60.1 ± 14.2	116/118	22.0 (18.0–29.0) vs 24.0 (19.0–29.0)	8.3 ± 2.7 vs 8.7 ± 2.9	NR	NR	NR
8. Lv 2020 (China)	Age 18–75, ICU patients with sepsis. Exclude: immunocompromised	1	117	58.7 ± 14.2 vs 60.2 ± 14.1	59/58	21.0 (19.0–28.0) vs 23.0 (20.0–29.0)	8.6 ± 2.9 vs 8.9 ± 3.1	NR	NR	NR
9. Jamali Moghadam Siahkali 2021 (Iran)	Age ≥ 18, COVID-19 with clinical manifestation of ARDS or myocarditis Exclude: G6PD, ESRF	1	60	57.53 ± 18.27 vs 61.00 ± 15.90	30/30	NR	NR	NR	NR	NR
10. Kassem 2021 (Egypt)	Age 18–64, TRALI ≤ 6 h of transfusion. Exclude: ever had kidney stone, G6PD, AKI, immunocompromised	2	80	52.6 ± 11.6 vs 47.2 ± 13.7	45/35	18.7 ± 8.1 vs 21.1 ± 7.1	6.7 ± 2.9 vs 5.9 ± 2.8	NR	NR	NR
11. Mahmoodpoor 2021 (Iran)	Age: 18–80, severe pneumonia in ICU with CURB-65 score > 3. Exclude: vitamin C allergy, end-stage lung disease/malignancy, G6PD, DKA, active kidney stone	1	80	56.9 ± 12.3 vs 58.3 ± 13.1	46/34	24.5 ± 5.4 vs 22.7 ± 4.3	12.5 ± 2.7 vs 10.7 ± 2.7	117.1 ± 72.34 vs 129.3 ± 77.0	96 h: 449.7 ± 150.0 vs 93.0 ± 58.7	NR
12. Zhang 2021 (China)	Age ≥ 18 and < 80, COVID-19, PF ratio < 300. Exclude: G6PD, end-stage pulmonary disease	3	56	66.3 ± 11.2 vs 67.0 ± 14.3	36/20	13.5 (10.3–15.8) vs 14.0 (11.0–16.0)	NR	NR	NR	59.7 ± 11.2 vs 64.4 ± 9.3
13. Ap 2022 (India)	Sepsis defined by sepsis 3. Exclude: chronic kidney disease, chronic alcoholics, immunocompromised	1	40 (Group 1 and 4)	All: 52.4 ± 13.7	28/12	NR	NR	21.7 ± 10.0 vs 17.5 ± 7.9	Day 6: 22.8 ± 12.0 vs NR	NR
14. Rosengrave 2022 (New Zealand)	Septic shock with organ failure. Exclude: G6PD	1	40	69 (64–76) vs 66 (57–71)	27/13	SAPSII: 50 (41–49) vs 49 (42–58)	8.5 (6.8–11) vs 9.0 (7.8–10)	10 (4–13) vs 8.2 (4.7–11)	72 h: 408 (227–560) vs 4.4 (3.1–8.8)	Median weight: 80 kg
15. Wacker 2022 (USA)	Age ≥ 18, septic shock (≤ 24 h of diagnosis). Exclude: cardiac arrest, cardiac surgery 48 h, history of kidney stone, G6PD, ESRF	5	124	68.9 (60.1–79.9) vs 73.0 (60.9–80.0)	63/61	22 (16.5–28) vs 23 (17–28.5)	10 (7–11) vs 9 (7–12)	NR	NR	NR
16. Lamontagne 2022 (International)	Proven or suspected infection and receiving vasopressors ≤ 24 h of ICU admission. Exclude: known kidney stone within past 1 year	35	863	65.0 ± 14.0 vs 65.2 ± 13.8	538/324	24.2 ± 7.4 vs 24.1 ± 7.9	10.2 ± 3.4 vs 10.1 ± 3.7	20.6 ± 70.6 vs 19.1 ± 39.7	NR	84.9 ± 22.6 vs 85.6 ± 26.6

The results are presented in this sequence: intervention (low-dose vs high dose) vs control. Fowler 2019 reported mSOFA: no bilirubin score because of many missing values. To convert vitamin C from umol/L to mg/dL, divide values by 56.78

*AKI* acute kidney injury, *DKA* diabetic ketoacidosis, *ESR*F end-stage renal failure, *F* female, *G6PD* glucose-6-phosphate dehydrogenase deficiency, *M* male, *NR* not reported, *TRALI* transfusion-related acute lung injury

### Study quality assessments

**Fig. 1:**
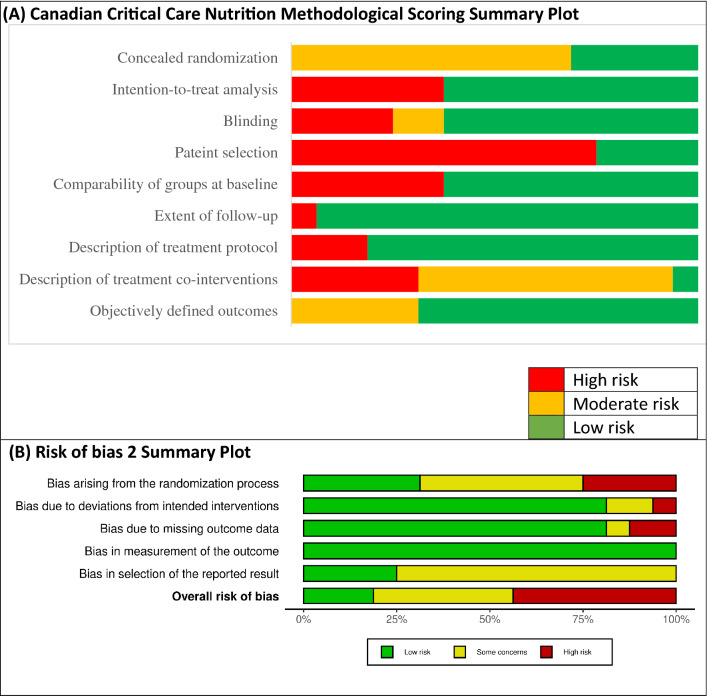
Summary of **A** Canadian critical care nutrition methodological scoring and **B** risk of bias 2

The CCN score of the studies ranged from 5 to 12, with a median score of 9. Additional file 1: Table S3 shows the detailed CCN score for all the included trials. In general, studies had low risk-of-bias in the extent of follow-up, treatment protocol, and outcome measurements; moderate risk-of-bias in randomization, baseline characteristics, analytical methods, and blinding; and high risk-of-bias in patient selection and co-interventions (Fig. 1A). Additional file 1: Figure S2 shows the ROB2 traffic light plot for each study. Overall, 3 (18.8%) studies had low risk of bias, 6 (37.5%) had some concerns, and 7 (43.8%) had high risk of bias. Most studies had risk of bias arising from the randomization process and in selection of the reported result (Fig. 1B).

### Study intervention

Additional file 1: Table S4 summarizes the study interventions. Three studies had more than one group of vitamin C intervention [19, 35, 36] in which a lower and a higher dose of vitamin C were administered to different groups of patients. The events before IVVC commencement were different in most studies (e.g., ICU admission/randomization/pressor initiation/post-abdominal surgery/head trauma/diagnosis of acute respiratory distress syndrome), and 7 studies started IVVC < 24 h of an event, whereas 9 studies started IVVC > 24 to ≤ 96 h after an event. The duration of intervention ranged from 3 to 7 days, with 11 studies administering IVVC for ≤ 4 days. The total vitamin C received per day ranged from 450 to 24,000 mg, with 7 (43.8%) studies administered high-dose IVVC (≥ 10,000 mg/day). Three studies administered IVVC through continuous infusion [26, 40, 42].

Additional file 1: Tables S5 and S6 summarize the clinical outcomes and adverse events, respectively.

### Results of the primary outcome

**Fig. 2:**
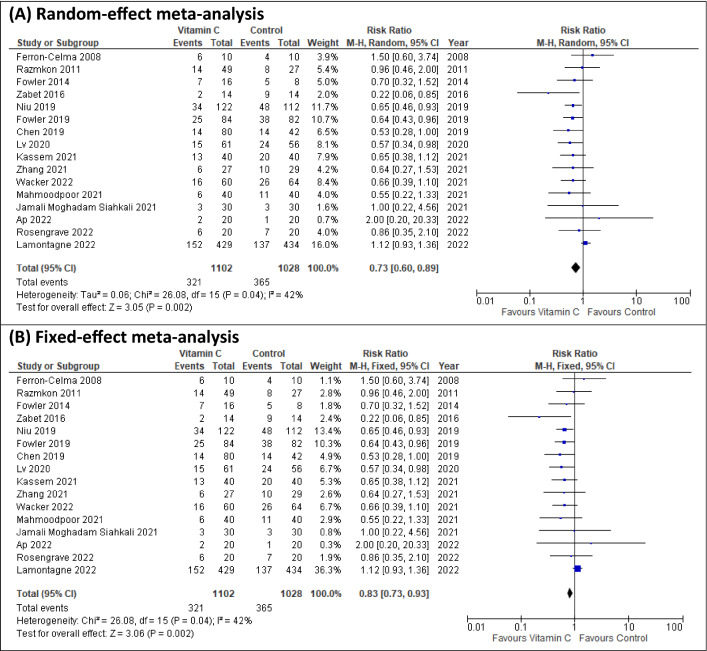
Overall mortality. **A** Random-effect meta-analysis, and **B** sensitivity analysis with fixed-effect meta-analysis

**Fig. 3:**
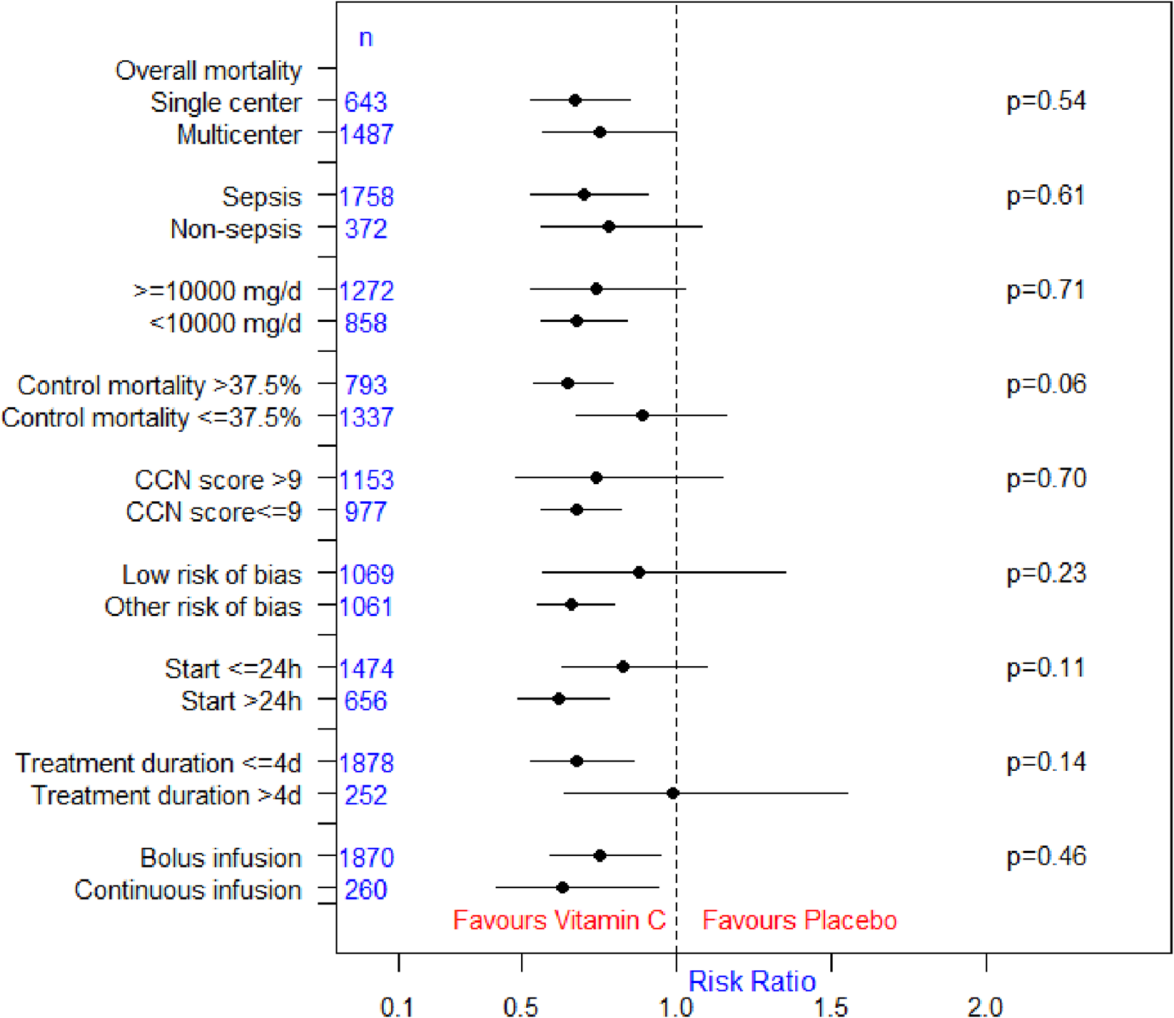
Subgroup analysis for overall mortality. *CCN* critical care nutrition

In the aggregated estimate, we found evidence for a reduction in overall mortality (RR 0.73, 95% CI 0.60–0.89; *p* = 0.002; *I*^2^ = 42%; 16 studies; Fig. 2A) associated with IVCC. In sensitivity analyses, there was still evidence for a reduction in overall mortality when fixed-effect model was used (RR 0.83, 95% CI 0.73–0.93; *p* = 0.002; Fig. 2B). We found no evidence of subgroup differences in our predefined subgroups of single vs multicenter studies, sepsis vs non-sepsis patients, and higher vs lower dose of IVVC (Fig. 3 and Additional file 1: Figures S3–S5). In our *post-hoc* subgroup analyses we found no evidence of subgroup differences between subgroups of studies with >  vs ≤ 9 CCN score, low vs other risk of bias, intervention commenced ≤  vs > 24 h, duration of treatment of >  vs ≤ 4 days, and bolus vs continuous infusion (Fig. 3 and Additional file 1: Figures S7–S11). In the subgroup analysis of above vs below median control group mortality, we observed a trend towards significant subgroup differences (*p* = 0.06). That is, IVVC monotherapy may benefit sicker (RR 0.65, 95% CI 0.54–0.79; *p* < 0.00001; *I*^2^ = 0%; 8 studies) but not the less sick patients (RR 0.89, 95% CI 0.68–1.16; *p* = 0.38; *I*^2^ = 18%; 8 studies) (Additional file 1: Figure S6). In studies that reported baseline vitamin C deficit, we found no evidence of a treatment effect (Additional file 1: Figure S12). No evidence of funnel plot asymmetry was detected in the overall mortality analysis (*p* = 0.54; Additional file 1: Figure S33).

### Trial sequential analysis

**Fig. 4:**
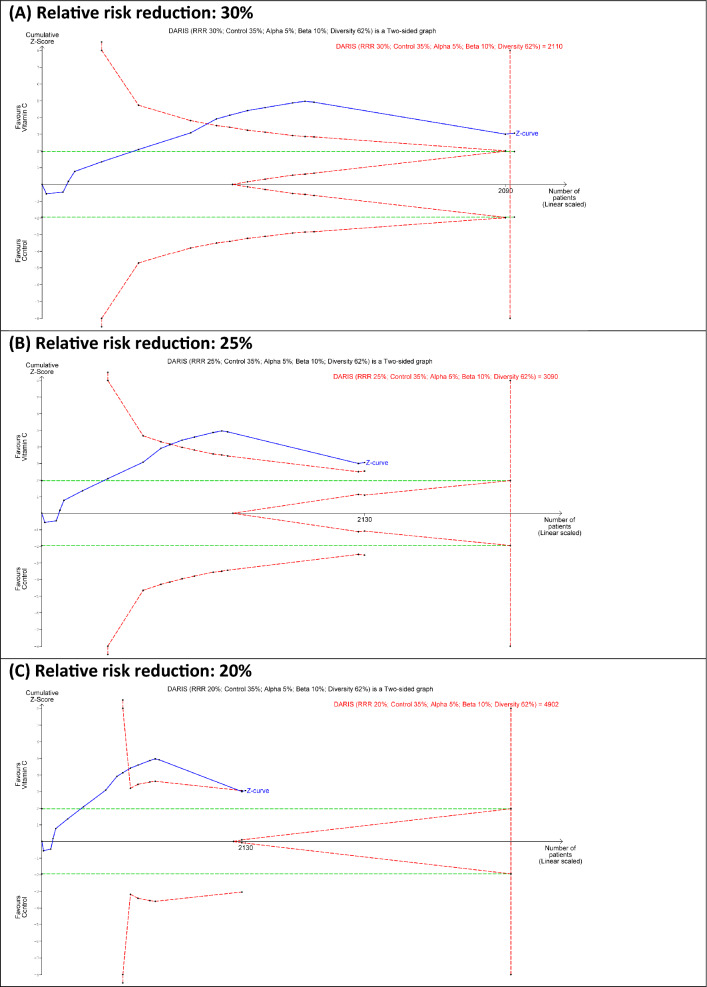
Trial sequential analysis for overall mortality. DARIS: diversity-adjusted required information size; RRR: relative risk reduction. The *Z* curve in blue measures the treatment effect (pooled relative risk). The green parallel lines are the boundaries of conventional naïve alpha of 5%, and the TSA boundaries are in red. A treatment effect outside the TSA boundaries of benefit or harm indicates that there is reliable evidence for a treatment effect, and a treatment effect within the futility zone (the triangle between the green parallel lines) indicates that there is reliable evidence of no treatment effect

The TSA graphs are presented in Fig. 4 and Additional file 1: Figures S34–S39 and summarized in Table 2. TSA confirmed the mortality benefits of IVVC monotherapy with high certainty for treatment effects of 30% and 25%. Although a larger sample size is required for a treatment effect of 20%, there was a trend towards significant mortality risk reduction (TSA adjusted RR 0.731, 95% CI 0.532–1.003). Sensitivity analysis using the BT model (Additional file 1: Table S7) confirmed the mortality benefits of IVVC monotherapy with high certainty for treatment effects of 30%, 25% and 20%.

**Table 2:** Trial sequential analysis for overall mortality

Relative Risk reduction, %	Control group event rate, %	*I*^2^, %	*D*^2^, %	RIS	RIS achieved	TSA adjusted RR (95% CI)	Results
Overall analysis (16 studies; *n* = 2130)							
30%	35%	42	62	2110	-	0.731 (0.595–0.897)	Sig risk reduction
25%	35%	42	62	3090	-	0.731 (0.563–0.949)	Sig risk reduction
20%	35%	42	62	4902	43.5%	0.731 (0.532–1.003)	Trend to sig reduction
Subgroup with lower control group mortality (8 studies; *n* = 1337)							
30%	30%	18	60	2489	53.7%	0.889 (0.605–1.307)	Uncertain
25%	30%	18	60	3653	36.6%	0.889 (0.560–1.412)	Uncertain
20%	30%	18	60	5811	23.0%	0.889 (0.496–1.596)	Uncertain
Subgroup with higher control group mortality (8 studies; *n* = 793)							
30%	45%	0	0	545	–	0.651 (0.530–0.801)	Sig risk reduction
25%	45%	0	0	793	–	0.651 (0.535–0.793)	Sig risk reduction
20%	45%	0	0	1251	–	0.651 (0.506–0.839)	Sig risk reduction

DerSimonian–Laird Random effect model, Alpha 5%, beta 10%

*RIS* required information size, *TSA* Trial sequential analysis

Similarly, TSA confirmed the mortality benefits of IVVC monotherapy with high certainty in the subgroup of studies that recruited patients with higher control group mortality for treatment effects of 30%, 25% and 20%, and similar results were shown in the sensitivity analysis using the BT model. In contrast, in the subgroup of studies that recruited patients with lower control group mortality, larger sample sizes are required for treatment effects of 30%, 25% and 20%. While the BT model showed that further trials are futile for treatment effect of 30% and 25%, and larger sample size is required for treatment effect of 20%

### Result of the secondary outcomes

IVVC monotherapy is associated with significant reduction in 28-day mortality (RR 0.71, 95% CI 0.53, 0.95; *p* = 0.02; *I*^2^ = 55%; 9 studies; Additional file 1: Figure S13). Similar to the main analysis, we found no evidence of subgroup differences for 28-day mortality for all the (Additional file 1: Figures S13–S21) subgroups analyses except for the subgroup analysis based on median control group mortality (Additional file 1: Figure S16). There was a significant subgroup differences for above vs below median control group mortality (*p* = 0.0003) in which IVVC monotherapy was associated with significant mortality reduction in trials that enrolled sicker but not the less sick patients (Additional file 1: Figure S16). Additional file 1: Figure S22 shows the summary of the subgroup analysis for 28-day mortality.

We found no evidence that IVVC impacted longer term mortality (RR 0.98, 95% CI 0.82, 1.19; *p* = 0.86; 4 studies) [11, 35, 38, 41], duration of mechanical ventilation (MD − 1.71, 95% CI − 5.94, 2.52; *p* = 0.43; *I*^2^ = 95%; 3 studies), ICU (MD − 0.63, 95% CI − 2.01, 0.75; *p* = 0.37; *I*^2^ = 35%; 7 studies) and hospital (MD − 0.55, 95% CI − 3.0.98, 2.88; *p* = 0.75; *I*^2^ = 0%; 4 studies) length of stays. No significant differences were found for incidences of AKI (RR 0.95, 95% CI 0.81–1.11; *p* = 0.52; *I*^2^ = 0%; 4 studies) [11, 25, 26, 40] and RRT (RR 2.00, 95% CI 0.59–6.77; *p* = 0.27; *I*^2^ = 65%; 3 studies) [11, 40, 42]. (Additional file 1: Figures S23–S28).

In studies that reported the SOFA score, we observed a trend towards lower SOFA scores at the 96th hour for the IVVC group (MD − 0.82, 95% CI -1.77, 0.14; *p* = 0.09; *I*^2^ = 63%; Additional file 1: Figure S29) [11, 19, 26, 41]. Four studies reported the dose of vasopressors used. The studies reported either the total vasopressors usage in 72 [37] or 96 [19]h in μg/min, or the mean vasopressor usage in μg/min [26] or units/min [41]. When pooled, we found no difference in vasopressor usage between groups (SMD − 0.26, 95% CI − 0.83, 0.31; *p* = 0.37; Additional file 1: Figure S30). Ten studies reported the duration of vasopressors but mean and SD were available in 3 studies [19, 26, 37]. When pooled, IVVC seemed to be associated with a reduced duration of vasopressors (MD − 0.79 days, 95% CI − 1.24, − 0.34; *p* = 0.0006; Additional file 1: Figure S31). Additional descriptions of these results are available in the Additional file 1.

### Adverse events

The adverse event rate was similar between groups (RR 1.04, 95% CI 0.90, 1.20; *p* = 0.59; *I*^2^ = 0%; 12 studies; Additional file 1: Figure S32).

#### Certainty of the evidence

The overall certainty of the evidence using GRADE was rated as low (Table 3). The certainty of the evidence was downgraded due to serious risk of bias (only 3/16 of the included trials are of low risk of bias) and inconsistency (heterogeneity *I*^2^ is 42%)

**Table 3:** GRADE certainty assessment and summary of findings table for overall mortality

Certainty assessment							Summary of findings				
Participants (studies) follow-up	Risk of bias	Inconsistency	Indirectness	Imprecision	Publication bias	Overall certainty of evidence	Study event rates (%)		Relative effect (95% CI)	Anticipated absolute effects	
							With placebo	With Intravenous Vitamin C Monotherapy		Risk with placebo	Risk difference with Intravenous Vitamin C Monotherapy
Overall mortality											
2130 (16 RCTs)	Serious^a^	Serious^b^	Not serious	Not serious^c^	none	⨁⨁◯◯ Low	365/1028 (35.5%)	321/1102 (29.1%)	RR 0.73 (0.60 to 0.89)	355 per 1,000	96 Fewer per 1000 (from 142 to 39 fewer)

*CI* confidence interval, *MD* mean difference, *RR* risk ratio, *SMD* standardised mean difference

^a^3/16 of the included trials are of low risk of bias, accounting for 30.7% of the weight in random-effect meta-analysis, while 7/16 of the included trials are of high risk of bias, accounting for 40.6% of the weight in random-effect meta-analysis. However, result of the sensitivity analysis using fixed-effect meta-analysis (low risk of bias studies account for 48.5% of the weight) is consistent with the result of random-effect meta-analysis. Therefore, the result is downgraded by one instead of two levels

^b^The overall heterogeneity *I*^2^ is 42%

^c^Both sensitivity analysis using the fixed-effect model meta-analysis, and the result of trial sequential analysis are similar to the main finding

## Discussion

### Summary of main findings

This SRMA of RCTs demonstrated that IVVC monotherapy is associated with a significant reduction in overall mortality. This finding is consistent with TSA and sensitivity analysis using the fixed-effect model meta-analysis. However, the certainty of evidence is low due to the serious risk of bias and inconsistency. On the other hand, based on limited analyzable studies, IVVC monotherapy did not affect the duration of mechanical ventilation, length of ICU and hospital stay, organ failure score at 96 h, vasopressors dose and duration, and incidences of AKI and RRT. The signal of mortality reduction is not modified by the vitamin C dose administered, whether septic patients were included exclusively, single/multicenter trial, the trial quality based on median CCN score or ROB2, timing of commencement, or duration of treatment. However, in sicker patients (higher median control group mortality rate), IVVC monotherapy seemed to be associated with a significant reduction in overall mortality. IVVC did not specifically reduce mortality in the few available studies that reported patients with baseline vitamin C deficiency. No evidence of adverse events or safety issues was found.

### Interpretation of the results in the context of other evidence

Our SRMA is the first to robustly explore the effects of IVVC monotherapy in critically ill patients through various subgroup analyses and TSA. In contrast, previous SRMA included trials that administered both IVVC monotherapy and combination therapy and found no significant effect of IVVC on 28‐day to 1‐year mortality [10]. The overall analysis of the most recent SRMA, among patients with severe infection, found a significant mortality reduction at hospital discharge or 30 days [12]. However, they concluded with moderate certainty that IVVC increased 90-day mortality (a non-statistical significant finding, RR 1.07, 95% CI 0.94–1.21) based on a meta-analysis of 5 trials with low risk of bias. Notably, 3 of these 5 trials investigated IVVC combination therapy [43–45]. They further compared IVVC monotherapy and combination therapy and found a significant test of subgroup differences in which IVVC monotherapy (but not combination therapy) had mortality benefits—a similar finding in our previous SRMA [8, 12]. Statistically significant finding in meta-analysis may be a type-1 error due to low quality or inadequately powered trials, publication bias, and/or repeated significant testing [46].The TSAs and sensitivity analysis in our SRMA indicated that the risk of type-1 error in finding a significant mortality benefits of IVVC monotherapy in our meta-analysis is very low.

Our SRMA included the most recent multicenter LOVIT trial [11] which found a higher incidence of a composite outcome of persistent organ dysfunction (need for vasopressor, renal replacement therapy and/or mechanical ventilation) plus death on trial day 28. The LOVIT trial did not demonstrate increased mortality at 28 days or 6 months, but when they combined 28-day mortality with persistent organ failure, they achieved a significant result on the composite outcome. It is worth pointing out that the authors acknowledged that the analysis of the primary outcome was very fragile; statistical significance was lost when analyzed using different adjustment techniques or when analyzed in unadjusted fashion. Nevertheless, when we examined the whole corpus of evidence including the LOVIT trial results, we found mortality benefits of IVVC monotherapy, albeit with low certainty. Furthermore, in this corpus of evidence, we found no evidence of more or persistent organ dysfunction, since we observed no differences in SOFA score at 96 h, dose of vasopressors, incidences of AKI and RRT, duration of mechanical ventilation, length of ICU and hospital stays, and longer term mortality. In addition, the days on vasopressors were significantly shorter. However, these secondary outcomes were from limited analyzable studies and these findings may be impacted by survivorship bias [47]. Of note, the LOVIT investigators were unable to explain a putative mechanism of harm from their a priori defined biomarkers of tissue dysoxia, inflammation, and endothelial injury, which was measured up to day 7. Likely, the LOVIT trial did not harm patients but definitively showed a lack of treatment benefit.

One may argue that our findings could be influenced mainly by smaller studies. Indeed, 1 in 3 large RCTs did not agree with meta-analysis of previous smaller studies due to small study effects arising from publication bias or poor study conduct, leading to the exaggeration of the intervention benefit. However, this may not the case for our review, since: (1) there was no subgroup differences between studies with higher vs lower quality score or low vs other risk-of-bias, (2) TSA showed very low risk of type-1 error, and (3) sensitivity analysis using the fixed effect model still showed the mortality benefit of IVVC monotherapy. That said, neither the analysis of the LOVIT trial nor our SRMA can explain the inconsistent findings of IVVC mechanistically, or from the perspective of trial or study intervention characteristics. One possible explanation is that LOVIT enrolled patients with a relatively lower risk of mortality, which is further discussed below.

### Subgroup and sensitivity analyses

We were unable to demonstrate different treatment effects based on dose, timing, duration of treatment or method of IVVC infusion. Although some may dispute that our subgroup analysis of < 24 h represents ‘early’ treatment within the pathophysiology of critical illness, as it was shown from a retrospective analysis that very early (< 6 h of onset of septic shock) administration of IVVC may confer the greatest benefit [48], we were not able to perform such subgroup analysis as almost all of the included trials started IVVC therapy at least 12 h after ICU admission (Additional file 1: Table S4). We found that IVVC monotherapy is associated with a significant reduction in mortality in patients with higher baseline mortality risk, and TSA supported this finding. In contrast, this mortality benefit is not found in patients with lower baseline mortality risk and TSA suggested more studies are needed to reach the required information size for a more definitive conclusion. It is unfortunate that ongoing randomized trials of IVCC were stopped prematurely, presumably on the basis of LOVIT results [49–51].

The direction of the above results (based on baseline mortality risk) is similar to the subgroup analyses of the LOVIT trial in which patients with a lower (the lower 2 quartiles) predicted risk of death were found to have a higher rate of mortality or persistent organ dysfunction if they received IVVC, whereas patients with a higher predicted risk of death were unaffected. Since our SRMA had greater power, we were able to demonstrate the mortality benefits of IVVC monotherapy in the high-mortality control group trial. That said, we did not have enough power to confirm the treatment effect of IVVC monotherapy in patients with lower baseline mortality.

It remains a compelling concept that only patients with documented vitamin C deficiency would benefit from IVVC supplementation. However, we could not demonstrate a beneficial effect of IVVC therapy in patients with reported baseline vitamin C deficiency, probably due to lack of power, since only a few studies reported the patients’ baseline vitamin C level.

It must be noted that the studies including in this SRMA are investigating the administration of Vitamin C at supraphysiological dose (range: 3000–24,000 mg/day for a 70 kg adult, except for one study that administered a relatively lower dose of 450 mg/day [34]; Additional file 1: Table S4) through the intravenous route. Beyond acting as an essential nutrient, such high dose will exert a ‘drug-like’ effect to the body, this is known as pharmaconutrition. The mortality benefit observed may be due to the stronger effect of the anti-inflammation, immune-enhancing and wound healing functions, among others, exerted by the pharmaconutrition dose of Vitamin C [1, 52]; however, the exact underlying mechanism remained to be investigated. On the other hand, pharmaconutrition is not without any risk, which have to be considered carefully. In our analysis, however, we did not find an increased risk of adverse event, and this is further discussed below.

### Adverse events

The lack of adverse events is consistent with the findings of a recent scoping review of 74 studies [53]. The scoping review included all studies that administered high-dose (6 g/d, 75 mg/kg/d or 3 g/m^2^/d) IVVC in adult patients. The specific adverse events attributed to high-dose IVVC found were oxalate nephropathy, hypernatremia, hemolysis in patients with glucose-6-phosphate dehydrogenase (G6PD) deficiency, glucometer error, and kidney stones. However, the authors found no evidence that IVVC is more harmful than placebo in double-blind RCTs. The authors recommended avoiding high-dose IVVC in patients with known or suspected G6PD deficiency. Our review identified 8/16 (50%) of the trials explicitly stated that patients with G6PD deficiency were excluded, and it is unclear if other studies exclude this group of patients. Due to a lack of data, the scoping review did not endorse the use of IVVC in critically ill patients, whereas we found no evidence of increased adverse events in our review of a large group of critically ill patients from RCTs.

### Strengths and limitations

Our SRMA has several limitations. Although we did not detect a higher incidences of organ dysfunction associated with IVVC, we are unable to confirm whether organ dysfunction persisted for a longer period (i.e., 28 days) in patients with preexisting organ dysfunction as the follow-up period for organ failure was short (≤ 7 days) in most of the included trials. The limited number of low risk of bias studies and the high heterogeneity (*I*^2^ = 42% and *D*^2^ = 62%) weaken the inferences we can make from our overall findings. In addition, TSA and several of our subgroup and sensitivity analyses were conducted *post-hoc*, and we did not adjust for multiplicity of testing (Additional file 1: Table S8). Accordingly, the results of such analysis should be considered hypothesis-generating. Furthermore, secondary outcomes are reported in smaller number of studies and not evaluated by TSA and GRADE. Therefore, their findings should not be overinterpreted.

The strength of our SRMA is demonstrated with the extensive subgroup and sensitivity analysis to explore the treatment effect of IVVC monotherapy. We also employed TSA to reduce the risk of type-1 or type-2 error in our findings.

## Conclusion

Our SRMA found that IVVC monotherapy may be associated with reduced mortality in critically ill patients, a finding that is supported by TSA. However, the certainty of the evidence is low due to serious risk of bias and inconsistency of trial results. The use of IVVC monotherapy appears to be safe with no higher incidences of adverse events observed in these randomized trials. We found no evidence that IVVC monotherapy is associated with higher incidences of organ dysfunction in the short-term; its effect on long-term organ dysfunction remains to be fully investigated. Sicker patients may benefit the most from this therapy; however, this finding is considered hypothesis-generating. We suggest that the quest to search for the optimal dosage, timing, treatment duration as well as which critically ill patient population that may benefit the most from this therapy should be continued within the boundaries of well-designed RCTs.

## Supplementary Information

**Additional file 1: **Methodology. PRISMA 2020 checklist. Results. **Table S1** Search strategy. **Table S2** List of excluded studies. **Table S3** Critical care nutrition methodological system. **Table S4** Intervention. **Table S5** Outcomes summary. **Table S6** Summary of adverse events. **Table S7** Trial sequential analysis for overall mortality (sensitivity analysis). **Table S8** Differences between protocol and review. **Figure S1** PRISMA flowchart. **Figure S2** Risk of bias 2 traffic light plot. **Figure S3** Overall mortality (single vs multicenter trials). **Figure S4** Overall mortality (sepsis vs non-sepsis). **Figure S5** Overall mortality (higher ≥10000 mg/day vs lower dose). **Figure S6** Overall mortality (median control group mortality > vs ≤ 37.5%). **Figure S7** Overall mortality (median CCN score >9 vs ≤9). **Figure S8** Overall mortality (low vs other risk of bias). **Figure S9** Overall mortality (start of intervention ≤ vs >24 h of ICU admission/septic shock/pressor initiation etc.). **Figure S10** Overall mortality (duration of treatment > vs ≤4 days). **Figure S11** Overall mortality (bolus vs continuous infusion). **Figure S12** Overall mortality sensitivity analysis studies that measured and reported baseline vitamin C deficit. **Figure S13** 28-day mortality (single vs multicenter). **Figure S14** 28-day mortality (sepsis vs non-sepsis). **Figure S15** 28-day mortality (higher dose ≥10000 mg/day vs lower dose). **Figure S16** 28-day mortality (median control group mortality > vs ≤ 37.5%). **Figure S17** 28-day mortality (median CCN score >9 vs ≤9). **Figure S18** 28-day mortality (low vs other risk of bias). **Figure S19** 28-day mortality (start of intervention ≤ vs >24h of ICU admission/septic shock/pressor initiation etc.). **Figure S20** 28-day mortality (Duration of treatment > vs ≤4 days). **Figure S21** 28-day mortality (bolus vs continuous infusion). **Figure S22** Summary of subgroup analysis for 28-day mortality. **Figure S23** Longer term mortality (≥60 days). **Figure S24** Duration of mechanical ventilation. **Figure S25** ICU length of stay. **Figure S26** Hospital length of stay. **Figure S27** Incidence of acute kidney injury. **Figure S28** Incidence of renal replacement therapy. **Figure S29** Sequential organ failure assessment (SOFA) score at 96h. **Figure S30** Dose of vasopressors. **Figure S31** Days on vasopressors. **Figure S32** (a) Adverse events. (b) Adverse events (with continuity correction by adding 0.01 to cells with zero events). **Figure S33** Funnel plot for overall mortality. **Figure S34** TSA for overall mortality—subgroup analysis in trials with below median control group mortality—relative risk reduction 30%. **Figure S35** TSA for overall mortality—subgroup analysis in trials below median control group mortality—relative risk reduction 25%. **Figure S36** TSA for overall mortality—subgroup analysis in trials below median control group mortality (≤37.5%)—Relative risk reduction 20%. **Figure S37** TSA for overall mortality—subgroup analysis in trials above (>37.5%) median control group mortality—relative risk reduction 30%. **Figure S38** TSA for overall mortality—subgroup analysis in trials above (>37.5%) median control group mortality—relative risk reduction 25%. **Figure S39** TSA for overall mortality—subgroup analysis in trials above (>37.5%) median control group mortality—relative risk reduction 20%.

## Data Availability

All data generated and/or analyzed during the current study are included within the published article and its additional files. The standardized data abstraction form and the critical care nutrition methodology scoring system is available at www.criticalcarenutrition.com

## Abbreviations

AKI: Acute kidney injury
APACHE II: Acute physiology and chronic health evaluation II
CCN: Critical Care Nutrition
CI: Confidence interval
G6PD: Glucose-6-phosphate dehydrogenase
GRADE: Grading of Recommendations Assessment, Development, and Evaluation
ICU: Intensive care unit
IVVC: Intravenous vitamin C
LOVIT: Lessening organ dysfunction with VITamin C trial
MD: Mean difference
RCT: Randomized controlled trial
RIS: Required information size
ROB: Risk of bias
RR: Relative risk or risk ratio
RRR: Relative risk reduction
RRT: Renal replacement therapy
SD: Standard deviation
SMD: Standardized mean difference
SRMA: Systematic review and meta-analysis
SOFA: Sequential organ failure assessment
TSA: Trial sequential analysis
