# Supplementary material for: Intravenous vitamin C monotherapy in critically ill patients: a systematic review and meta-analysis of randomized controlled trials with trial sequential analysis
Source: Ann Intensive Care. 2023 Mar 7;13:14. doi: 10.1186/s13613-023-01116-x (PMC9990974; doi:10.1186/s13613-023-01116-x)

**Additional File**

**Intravenous Vitamin C Monotherapy in critically ill patients: a systematic review and meta-analysis of randomized controlled trials with trial sequential analysis**

**Zheng-Yii Lee, PhD**

Department of Anaesthesiology, Faculty of Medicine, University of Malaya, 50603 Kuala Lumpur, Malaysia.

ORCID: 0000-0003-4505-7476

Email: zheng_yii@hotmail.com

**Luis Ortiz-Reyes, MSc**

Clinical Evaluation Research Unit, Department of Critical Care Medicine, Queen’s University, Kingston, ON, K7L 3N6, Canada

Email: laor@queensu.ca

**Charles Chin Han Lew, PhD**

Department of Dietetics & Nutrition, Ng Teng Fong General Hospital, Singapore, Singapore

1 Jurong East Street 21, Singapore 609606

ORCID: 0000-0001-6410-3859

Email: Charles_lew@nuhs.edu.sg

**M. Shahnaz Hasan, MBBS, MMed**

Department of Anesthesiology, Faculty of Medicine, University of Malaya, 50603 Kuala Lumpur, Malaysia.

Email: shahnaz@ummc.edu.my

**Lu Ke, PhD**

Department of Critical Care Medicine, Jinling Hospital, Medical School of Nanjing University, No. 305 Zhongshan East Road, Nanjing, 210000, Jiangsu Province, China

National Institute of Healthcare Data Science, Nanjing University, Nanjing, China

Email: kkb9832@gmail.com

**Jayshil J Patel, MD**

Associate Professor of Medicine

Division of Pulmonary & Critical Care Medicine, Medical College of Wisconsin, Milwaukee, Wisconsin, United States

ORCID: 0000-0003-0663-7670

Email: jpatel2@mcw.edu

**Christian Stoppe, MD**

Department of Anesthesiology, Intensive Care, Emergency and Pain Medicine, University Hospital Wuerzburg, DHZB Charité Berlin

Email: christian.stoppe@gmail.com

**Daren K Heyland, MSc**

Clinical Evaluation Research Unit, Department of Critical Care Medicine, Queen’s University, Kingston, ON, K7L 3N6, Canada

Email: dkh2@queensu.ca

**Correspondence to**

**Daren K Heyland, MSc**

Clinical Evaluation Research Unit, Department of Critical Care Medicine, Queen’s University, Kingston, ON, K7L 3N6, Canada

Email: [dkh2@queensu.ca](mailto:dkh2@queensu.ca)

**Table of Contents**

[Methodology (additional descriptions) 4](#_Toc124250886)

[PRISMA 2020 Checklist 5](#_Toc124250887)

[Results (additional descriptions) 8](#_Toc124250888)

[Table S1: Search Strategy 9](#_Toc124250889)

[Table S2: List of excluded studies 17](#_Toc124250890)

[Table S3: Critical Care Nutrition Methodological System 22](#_Toc124250891)

[Table S4: The Intervention 23](#_Toc124250892)

[Table S5: Outcomes summary 25](#_Toc124250893)

[Table S6: Summary of Adverse Events 28](#_Toc124250894)

[Table S7: Trial sequential analysis for overall mortality (sensitivity analysis) 29](#_Toc124250895)

[Table S8: Differences between protocol and review 30](#_Toc124250896)

[Figure S1: PRISMA flowchart 31](#_Toc124250897)

[Figure S2: Risk of bias 2 traffic light plot for Overall Mortality 32](#_Toc124250898)

[Figure S3: Overall mortality (single vs multicenter trials) 33](#_Toc124250899)

[Figure S4: Overall mortality (sepsis vs non-sepsis) 34](#_Toc124250900)

[Figure S5: Overall mortality (higher ≥10000 mg/day vs lower dose) 35](#_Toc124250901)

[Figure S6: Overall mortality (median control group mortality > vs ≤ 37.5%) 36](#_Toc124250902)

[Figure S7: Overall mortality (median CCN score >9 vs ≤9) 37](#_Toc124250903)

[Figure S8: Overall mortality (Low vs other risk of bias) 38](#_Toc124250904)

[Figure S9: Overall mortality (start of intervention ≤ vs >24h of ICU admission/septic shock/pressor initiation etc) 39](#_Toc124250905)

[Figure S10: Overall mortality (Duration of treatment > vs ≤4 days) 40](#_Toc124250906)

[Figure S11: Overall mortality (Bolus vs continuous infusion) 41](#_Toc124250907)

[Figure S12: Overall mortality: Sensitivity analysis: studies that measured and reported baseline vitamin C deficit 42](#_Toc124250908)

[Figure S13: 28-day mortality (single vs multicenter) 43](#_Toc124250909)

[Figure S14: 28-day mortality (sepsis vs non-sepsis) 44](#_Toc124250910)

[Figure S15: 28-day mortality (higher dose ≥10000 mg/day vs lower dose) 45](#_Toc124250911)

[Figure S16: 28-day mortality (median control group mortality > vs ≤ 37.5%) 46](#_Toc124250912)

[Figure S17: 28-day mortality (median CCN score >9 vs ≤9) 47](#_Toc124250913)

[Figure S18: 28-day mortality (low vs other risk of bias) 48](#_Toc124250914)

[Figure S19: 28-day mortality (start of intervention ≤ vs >24h of ICU admission/septic shock/pressor initiation etc.) 49](#_Toc124250915)

[Figure S20: 28-day mortality (Duration of treatment > vs ≤4 days) 50](#_Toc124250916)

[Figure S21: 28-day mortality (Bolus vs continuous infusion) 51](#_Toc124250917)

[Figure S22: Summary of subgroup analysis for 28-day mortality 52](#_Toc124250918)

[Figure S23: Longer-term mortality (≥60 days) 53](#_Toc124250919)

[Figure S24: Duration of mechanical ventilation 54](#_Toc124250920)

[Figure S25: ICU length of stay 54](#_Toc124250921)

[Figure S26: Hospital length of stay 55](#_Toc124250922)

[Figure S27: Incidence of acute kidney injury 55](#_Toc124250923)

[Figure S28: Incidence of renal replacement therapy 56](#_Toc124250924)

[Figure S29: Sequential organ failure assessment (SOFA) score at 96h 56](#_Toc124250925)

[Figure S30: Dose of vasopressors 57](#_Toc124250926)

[Figure S31: Days on vasopressors 57](#_Toc124250927)

[Figure S32: (a) Adverse events 58](#_Toc124250928)

[Figure S32: (b) Adverse events (with continuity correction by adding 0.01 to cells with zero events) 59](#_Toc124250929)

[Figure S33: Funnel plot for overall mortality 60](#_Toc124250930)

[Figure S34: TSA for Overall mortality - subgroup analysis in trials with below median control group mortality - Relative risk reduction 30% 61](#_Toc124250931)

[Figure S35: TSA for Overall mortality - subgroup analysis in trials below median control group mortality - Relative risk reduction 25% 62](#_Toc124250932)

[Figure S36: TSA for Overall mortality - subgroup analysis in trials below median control group mortality (≤37.5%) - Relative risk reduction 20% 63](#_Toc124250933)

[Figure S37: TSA for Overall mortality - subgroup analysis in trials above (>37.5%) median control group mortality - Relative risk reduction 30% 64](#_Toc124250934)

[Figure S38: TSA for Overall mortality - subgroup analysis in trials above (>37.5%) median control group mortality - Relative risk reduction 25% 65](#_Toc124250935)

[Figure S39: TSA for Overall mortality - subgroup analysis in trials above (>37.5%) median control group mortality - Relative risk reduction 20% 66](#_Toc124250936)

## Methodology (additional descriptions)

**Data Items**

First author, publication year, country, single/multicenter trial, sample size, description of the characteristics of the included patients, the timing, dose, and duration of the intervention.

Clinical outcomes including overall mortality, infectious complications, duration of mechanical ventilation, and ICU or hospital length of stay, the absolute value or change in sequential organ failure assessment (SOFA) score after the intervention, duration and dose of vasopressors, incidence of acute kidney injury (AKI) and renal replacement therapy (RRT), and adverse events.

**Trial sequential analysis**

SRMAs are consistently updated with data from new trials, and statistical significance is set at p<0.05 regardless of the number of multiple testing (due to trial addition) and total accrued sample size, which increases the risk of type-I error (Imberger et al. BMJ Open. 2016;6(8):e011890. doi:10.1136/bmjopen-2016-011890). Such statistical error can be detected via TSA. In contrast to conventional SRMA, TSA uses a set of pre-specified parameters (i.e., alpha, beta, estimated relative risk reduction, and between-trial heterogeneity) to calculate the required sample size (also known as required information size [RIS]) to detect a significant difference (if there is any). In controlling for type-I error, TSA is analogous to conducting a large RCT with multiple interim analyses in which statistical significance is most restrictive in the earlier trials (p-value much smaller than 0.05) and less restrictive as the sample size increases with each addition of a trial. TSA boundaries are constructed for this purpose and crossing the TSA boundaries indicates high certainty of either a positive or negative effect. Additionally, TSA controls type-II error by constructing futility boundaries. The Z-curve crossing the futility boundaries indicates evidence of an absence of effect, whereas being outside of the futility and TSA boundaries indicates the absence of evidence, and more trials are needed to confirm the presence or absence of an effect. (Thorlund et al. *User Manual for Trial Sequential Analysis (TSA)*. 2nd ed.; 2017).

The choice of relative risk reduction is based on the following:

In the IV Vitamin C monotherapy subgroup analysis: Agarwal et al (DOI: 10.1056/EVIDoa2200105) found an relative risk (RR) of 0.67 (95% CI 0.55-0.82) [See Figure S18 by Agarwal et al], while Patel et al (DOI: 10.1097/CCM.0000000000005320) found an RR of 0.64 (95% CI 0.49, 0.83). These are translated to a relative risk reduction of 33% (18%-45%) and 36% (17%-51%). Therefore, we chose an RRR of 30% based on these findings and conducted sensitivity analyses with RRR 25% and 20%.

## PRISMA 2020 Checklist

| **Section and Topic** | **Item #** | **Checklist item** | **Location where item is reported** |
| --- | --- | --- | --- |
| **TITLE** | | |  |
| Title | 1 | Identify the report as a systematic review. | Title page |
| **ABSTRACT** | | |  |
| Abstract | 2 | See the PRISMA 2020 for Abstracts checklist. | Page 4 |
| **INTRODUCTION** | | |  |
| Rationale | 3 | Describe the rationale for the review in the context of existing knowledge. | Page 6-7 |
| Objectives | 4 | Provide an explicit statement of the objective(s) or question(s) the review addresses. | Page 7 |
| **METHODS** | | |  |
| Eligibility criteria | 5 | Specify the inclusion and exclusion criteria for the review and how studies were grouped for the syntheses. | Page 8 |
| Information sources | 6 | Specify all databases, registers, websites, organisations, reference lists and other sources searched or consulted to identify studies. Specify the date when each source was last searched or consulted. | Page 8 |
| Search strategy | 7 | Present the full search strategies for all databases, registers and websites, including any filters and limits used. | Table S1 |
| Selection process | 8 | Specify the methods used to decide whether a study met the inclusion criteria of the review, including how many reviewers screened each record and each report retrieved, whether they worked independently, and if applicable, details of automation tools used in the process. | Page 8 |
| Data collection process | 9 | Specify the methods used to collect data from reports, including how many reviewers collected data from each report, whether they worked independently, any processes for obtaining or confirming data from study investigators, and if applicable, details of automation tools used in the process. | Page 8-9 |
| Data items | 10a | List and define all outcomes for which data were sought. Specify whether all results that were compatible with each outcome domain in each study were sought (e.g. for all measures, time points, analyses), and if not, the methods used to decide which results to collect. | Page 9 |
|  | 10b | List and define all other variables for which data were sought (e.g. participant and intervention characteristics, funding sources). Describe any assumptions made about any missing or unclear information. | Page 9 and Additional file page 4 |
| Study risk of bias assessment | 11 | Specify the methods used to assess risk of bias in the included studies, including details of the tool(s) used, how many reviewers assessed each study and whether they worked independently, and if applicable, details of automation tools used in the process. | Page 9 |
| Effect measures | 12 | Specify for each outcome the effect measure(s) (e.g. risk ratio, mean difference) used in the synthesis or presentation of results. | Page 9 |
| Synthesis methods | 13a | Describe the processes used to decide which studies were eligible for each synthesis (e.g. tabulating the study intervention characteristics and comparing against the planned groups for each synthesis (item #5)). | Page 9-10 |
|  | 13b | Describe any methods required to prepare the data for presentation or synthesis, such as handling of missing summary statistics, or data conversions. | Page 9-10 |
|  | 13c | Describe any methods used to tabulate or visually display results of individual studies and syntheses. | Page 10-12 |
|  | 13d | Describe any methods used to synthesize results and provide a rationale for the choice(s). If meta-analysis was performed, describe the model(s), method(s) to identify the presence and extent of statistical heterogeneity, and software package(s) used. | Page 10 |
|  | 13e | Describe any methods used to explore possible causes of heterogeneity among study results (e.g. subgroup analysis, meta-regression). | Page 10 |
|  | 13f | Describe any sensitivity analyses conducted to assess robustness of the synthesized results. | Page 10 |
| Reporting bias assessment | 14 | Describe any methods used to assess risk of bias due to missing results in a synthesis (arising from reporting biases). | Page 9 |
| Certainty assessment | 15 | Describe any methods used to assess certainty (or confidence) in the body of evidence for an outcome. | Page 12 |
| **RESULTS** | | |  |
| Study selection | 16a | Describe the results of the search and selection process, from the number of records identified in the search to the number of studies included in the review, ideally using a flow diagram. | Page 13, Fig S1 |
|  | 16b | Cite studies that might appear to meet the inclusion criteria, but which were excluded, and explain why they were excluded. | Table S2 |
| Study characteristics | 17 | Cite each included study and present its characteristics. | Table 1, Table S4-S6 |
| Risk of bias in studies | 18 | Present assessments of risk of bias for each included study. | Table S3, Fig 1, Fig S2 |
| Results of individual studies | 19 | For all outcomes, present, for each study: (a) summary statistics for each group (where appropriate) and (b) an effect estimate and its precision (e.g. confidence/credible interval), ideally using structured tables or plots. | Page 14-17; Fig 2, FigS3-21 |
| Results of syntheses | 20a | For each synthesis, briefly summarise the characteristics and risk of bias among contributing studies. | Page 13-14, Table S3, Fig 1, Fig S2 |
|  | 20b | Present results of all statistical syntheses conducted. If meta-analysis was done, present for each the summary estimate and its precision (e.g. confidence/credible interval) and measures of statistical heterogeneity. If comparing groups, describe the direction of the effect. | Page 14-17; Fig 2, FigS3-21 |
|  | 20c | Present results of all investigations of possible causes of heterogeneity among study results. | Page 15-17, FigS3-21 |
|  | 20d | Present results of all sensitivity analyses conducted to assess the robustness of the synthesized results. | Page 14, Fig 2; Page 15-16, Fig 4, Fig S34-39, Table 2 |
| Reporting biases | 21 | Present assessments of risk of bias due to missing results (arising from reporting biases) for each synthesis assessed. | Table S3, Fig 1, Fig S2 |
| Certainty of evidence | 22 | Present assessments of certainty (or confidence) in the body of evidence for each outcome assessed. | Page 17, Table 3 |
| **DISCUSSION** | | |  |
| Discussion | 23a | Provide a general interpretation of the results in the context of other evidence. | Page 18-19 |
|  | 23b | Discuss any limitations of the evidence included in the review. | Page 22 |
|  | 23c | Discuss any limitations of the review processes used. | Page 22, Table S8 |
|  | 23d | Discuss implications of the results for practice, policy, and future research. | Page 20, 21, 23 |
| **OTHER INFORMATION** | | |  |
| Registration and protocol | 24a | Provide registration information for the review, including register name and registration number, or state that the review was not registered. | Page 5 |
|  | 24b | Indicate where the review protocol can be accessed, or state that a protocol was not prepared. | Page 5 |
|  | 24c | Describe and explain any amendments to information provided at registration or in the protocol. | Table S8 |
| Support | 25 | Describe sources of financial or non-financial support for the review, and the role of the funders or sponsors in the review. | Page 33 |
| Competing interests | 26 | Declare any competing interests of review authors. | Page 33 |
| Availability of data, code and other materials | 27 | Report which of the following are publicly available and where they can be found: template data collection forms; data extracted from included studies; data used for all analyses; analytic code; any other materials used in the review. | Page 33 |

*From:*  Page MJ, McKenzie JE, Bossuyt PM, Boutron I, Hoffmann TC, Mulrow CD, et al. The PRISMA 2020 statement: an updated guideline for reporting systematic reviews. BMJ 2021;372:n71. doi: 10.1136/bmj.n71

## Results (additional descriptions)

**SOFA score**

Eight studies reported the change in SOFA score at 72 and 96 hours or day 6 and 7 after the intervention; these were not meta-analyzed as the reported summary measures are mostly in median. Among them, 4 studies reported a more favorable change in SOFA in the IVVC group ^18,19,37,38^, while 3 studies reported no difference in the change of SOFA score between groups ^37,39,40^. One study did not report the significance testing for the change in SOFA score ^26^.

**Duration of vasopressors**

Among studies that were not meta-analyzed, three studies found significantly shorter duration of vasopressors ^18,24,38^ and three studies no differences between groups ^11,40,41^. One study did not report the significance of testing between groups ^35^.

Note: The reference numbers are based on the reference of the main manuscript

## Table S1: Search Strategy

**a) Ovid MEDLINE(R) ALL <1946 to June 21, 2022>**

| **#** | **Query** | **Results from 22 Jun 2022** |
| --- | --- | --- |
| 1 | randomized controlled trial.pt. | 571,413 |
| 2 | controlled clinical trial.pt. | 94,916 |
| 3 | randomized.ab. | 565,827 |
| 4 | placebo.ab. | 229,309 |
| 5 | drug therapy.fs. | 2,503,682 |
| 6 | randomly.ab. | 385,128 |
| 7 | trial.ab. | 605,217 |
| 8 | groups.ab. | 2,368,843 |
| 9 | or/1-8 | 5,387,574 |
| 10 | (exp adolescent/ or exp child/ or exp infant/ or (infant disease* or childhood disease*).ti,ab,kf. or (adolescen* or babies or baby or boy? or boyfriend or boyhood or girlfriend or girlhood or child* or girl? or infan* or juvenil* or kid? or minors or minors* or neonat* or neo-nat* or newborn* or new-born* or paediatric* or peadiatric* or pediatric* or perinat* or preschool* or puber* or pubescen* or school* or teen* or toddler? or underage? or under-age? or youth*).ti,ab,kf. or (pediatric* or paediatric* or infan* or child* or adolescen* or young).jn,jw. or (pediatric* or paediatric* or infan* or child* or adolescen* or young).in.) not exp adult/ | 3,384,244 |
| 11 | 9 not 10 | 4,774,456 |
| 12 | (Animals/ or Models, Animal/ or Disease Models, Animal/) not Humans/ | 4,987,117 |
| 13 | ((animal or animals or canine* or dog or dogs or feline or hamster* or lamb or lambs or mice or monkey or monkeys or mouse or murine or pig or pigs or piglet* or porcine or primate* or rabbit* or rats or rat or rodent* or sheep* or veterinar*) not (human* or patient*)).ti,kf,jw. | 2,470,962 |
| 14 | 12 or 13 | 5,422,480 |
| 15 | 11 not 14 | 4,058,504 |
| 16 | Critical care/ | 58,431 |
| 17 | Critical illness/ | 36,239 |
| 18 | critical care.mp. | 83,733 |
| 19 | intensive care.mp. | 210,953 |
| 20 | critical illness.mp. | 42,131 |
| 21 | critically ill.mp. | 54,923 |
| 22 | Intensive care units/ | 66,377 |
| 23 | exp shock/ | 83,199 |
| 24 | sepsis/ | 67,423 |
| 25 | Shock.ti,kw. | 75,686 |
| 26 | Systemic inflammatory response syndrome.mp. | 10,374 |
| 27 | sepsis.mp. | 141,292 |
| 28 | septic shock.mp. | 25,799 |
| 29 | multiple organ dysfunction syndrome.mp. | 2,554 |
| 30 | multiple organ failure.mp. | 17,240 |
| 31 | cytokine release syndrome.ti,kw. | 717 |
| 32 | Respiratory Distress Syndrome/ | 23,190 |
| 33 | respiratory distress syndrome.mp. | 52,365 |
| 34 | acute lung injury.mp. | 17,401 |
| 35 | Burns/ | 47,393 |
| 36 | (burn$ adj3 patient$).ti,kw. | 7,276 |
| 37 | or/16-36 | 598,880 |
| 38 | exp Ascorbic Acid/ | 44,341 |
| 39 | (ascor* or l-ascor* or acidylina* or adenex* or afj c or agrumina* or alle?corb* or antiscorb* or arcavit* c or arkovital* c or ascelat* or ascofar* or ascomed* or asconvita* or askorbin* or austrovit* c or bentavit* c or c crivit* or c ine or c level or c lisa or c long or c monovit* or c prana or c rivitin* or c sol or c tamin* or c tonic or c tron or c vescent or c vicotrat* or c?vimin* or c vit* or c-will or cantan or cantaxin or catavin c or ce arom or ce major or ce quin* or ce?vi?sol or ce vit* or cebetate or cebicure or cebion* or cecap or cecon* or cecorb* or cecrisina or cedon* or cedoxon* or cee-500 or ceevifil or cegiolan or celaskon* or celin* or cenetone or cenol* or cequinyl or cereon or cergona or cescorbat or cetami* or cetebe or ceterapion* or cetrinets or ceva?in* or cevex or cevibram or cevig* or cevilat or cevimin* or cevisol or cevit* or cewin or chewcee or chivibit c or ci drol or ciamin* or ciergin or cifilina or cipca or cisir or cital or citamino or cith or citoascorbina or citoxyl or citran or citravite or citritabs or citrovitamina or civigor or civitin* or co biagini or concemin or cortalex).mp. | 77,780 |
| 40 | (xylo?ascorb* or dagrascorb* or dagravit c or dancimin c or davitamon c or dayvital or delo c or difvitamin c or dumovit* c or erftamin* c or esuron or esurvit* or flavettes or godabion c or gregovite c or hicee or hybrin* or ido c or ikacee or inovitan c or irocevit or irocevite or jarexin* or keto hexuronic acid lactone or lacivit* or laroscorbine or leder?c or lemascorb or limcee or limo ce or liqui cee or magnorbin* or mega-c?a or myascorbin or natrascorb or nybadol or paa 500 or parkovit c or pharmascorb* or pharmatovit* c or planavit* c or plivit* c or pro-c or proscorbin* or redoxon or ribena or scorbacid* or scorbettes or scorbex or scorbin c or scorbitol or scorbumine or scottavit* c or secorbate or sevalin or sigmavit* c or sodascorb* or sweetcee or synum c or take-c or tanvimil-c or testascorb* or ucemine c or upsa?c or vi?ci sin or vi dom c or vi-c 500 or vicef or vicelat or vicetrin or vici monico or viciman or vicin or vicitina or vicon or viforcit* or viscorin* or vita-cedol orange or vitac or vitace or vitacee or vitaci* or vitamin* C or vitaplex c or vitapric or vitapur c or vitasan c or vitascorb* or vitelix c or vitocee or vorange or wandervit* c or witamina c or xitix or xon-ce).mp. | 29,171 |
| 41 | or/38-40 | 88,840 |
| 42 | 15 and 37 and 41 | 415 |

**b) Embase Classic+Embase <1947 to 2022 June 21>**

| **#** | **Query** | **Results from 22 Jun 2022** |
| --- | --- | --- |
| 1 | Randomized controlled trial/ | 715,871 |
| 2 | Controlled clinical study/ | 466,193 |
| 3 | random$.ti,ab. | 1,812,832 |
| 4 | randomization/ | 94,206 |
| 5 | intermethod comparison/ | 284,460 |
| 6 | placebo.ti,ab. | 347,161 |
| 7 | (compare or compared or comparison).ti. | 598,183 |
| 8 | ((evaluated or evaluate or evaluating or assessed or assess) and (compare or compared or comparing or comparison)).ab. | 2,522,582 |
| 9 | (open adj label).ti,ab. | 97,397 |
| 10 | ((double or single or doubly or singly) adj (blind or blinded or blindly)).ti,ab. | 263,035 |
| 11 | double blind procedure/ | 198,515 |
| 12 | parallel group$1.ti,ab. | 29,553 |
| 13 | (crossover or cross over).ti,ab. | 118,179 |
| 14 | ((assign$ or match or matched or allocation) adj5 (alternate or group$1 or intervention$1 or patient$1 or subject$1 or participant$1)).ti,ab. | 383,905 |
| 15 | (assigned or allocated).ti,ab. | 452,765 |
| 16 | (controlled adj7 (study or design or trial)).ti,ab. | 413,894 |
| 17 | (volunteer or volunteers).ti,ab. | 274,665 |
| 18 | human experiment/ | 580,362 |
| 19 | trial.ti. | 369,086 |
| 20 | or/1-19 | 5,877,520 |
| 21 | (random$ adj sampl$ adj7 (cross section$ or questionnaire$1 or survey$ or database$1)).ti,ab. not (comparative study/ or controlled study/ or randomi?ed controlled.ti,ab. or randomly assigned.ti,ab.) | 9,140 |
| 22 | Cross-sectional study/ not (randomized controlled trial/ or controlled clinical study/ or controlled study/ or randomi?ed controlled.ti,ab. or control group$1.ti,ab.) | 313,352 |
| 23 | (((case adj control$) and random$) not randomi?ed controlled).ti,ab. | 19,912 |
| 24 | (Systematic review not (trial or study)).ti. | 212,682 |
| 25 | (nonrandom$ not random$).ti,ab. | 17,960 |
| 26 | Random field$.ti,ab. | 2,729 |
| 27 | (random cluster adj3 sampl$).ti,ab. | 1,444 |
| 28 | (review.ab. and review.pt.) not trial.ti. | 1,002,820 |
| 29 | we searched.ab. and (review.ti. or review.pt.) | 42,333 |
| 30 | update review.ab. | 122 |
| 31 | (databases adj4 searched).ab. | 51,405 |
| 32 | (rat or rats or mouse or mice or swine or porcine or murine or sheep or lambs or pigs or piglets or rabbit or rabbits or cat or cats or dog or dogs or cattle or bovine or monkey or monkeys or trout or marmoset$1).ti. and animal experiment/ | 1,157,204 |
| 33 | Animal experiment/ not (human experiment/ or human/) | 2,431,498 |
| 34 | or/21-33 | 3,995,946 |
| 35 | 20 not 34 | 5,211,936 |
| 36 | (exp adolescence/ or exp adolescent/ or exp child/ or exp childhood disease/ or exp infant disease/ or (adolescen* or babies or baby or boy? or boyfriend or boyhood or girlfriend or girlhood or child* or girl? or infan* or juvenil* or juvenile* or kid? or minors or minors* or neonat* or neo-nat* or neo-nat* or newborn* or new-born* or paediatric* or peadiatric* or pediatric* or perinat* or preschool* or puber* or pubescen* or school or school child* or school* or schoolchild* or schoolchild*).ti,ab,kw. or (pediatric* or paediatric* or infan* or child* or adolescen* or young).jn,jw. or (pediatric* or paediatric* or infan* or child* or adolescen* or young).in. or (teen* or toddler? or underage? or under-age? or youth*).ti,ab,kw.) not exp adult/ | 4,890,849 |
| 37 | 35 not 36 | 4,674,385 |
| 38 | (animal or animals or canine* or dog or dogs or feline or hamster* or lamb or lambs or mice or monkey or monkeys or mouse or murine or pig or pigs or piglet* or porcine or primate* or rabbit* or rats or rat or rodent* or sheep* or veterinar*).ti,kw,dq,jx. not (human* or patient*).mp. | 2,487,935 |
| 39 | (exp animal/ or exp juvenile animal/ or adult animal/ or animal cell/ or animal tissue/ or nonhuman/ or animal experiment/ or animal model/) not human/ | 7,899,074 |
| 40 | 38 or 39 | 8,011,611 |
| 41 | 37 not 40 | 4,288,816 |
| 42 | Intensive care/ | 138,826 |
| 43 | critical illness/ | 33,484 |
| 44 | critically ill patient/ | 58,009 |
| 45 | critical care.mp. | 66,778 |
| 46 | intensive care.mp. | 433,832 |
| 47 | critical illness.mp. | 41,086 |
| 48 | critically ill.mp. | 95,002 |
| 49 | intensive care unit/ | 198,792 |
| 50 | medical intensive care unit/ | 3,358 |
| 51 | surgical intensive care unit/ | 2,347 |
| 52 | neurological intensive care unit/ | 2,203 |
| 53 | exp shock/ | 167,564 |
| 54 | systemic inflammatory response syndrome/ | 14,383 |
| 55 | sepsis/ | 188,060 |
| 56 | septic shock/ | 62,719 |
| 57 | septicemia/ | 23,189 |
| 58 | urosepsis/ | 3,747 |
| 59 | multiple organ failure/ | 45,538 |
| 60 | Shock.ti,kw. | 96,500 |
| 61 | Systemic inflammatory response syndrome.mp. | 17,389 |
| 62 | sepsis.mp. | 255,904 |
| 63 | septic shock.mp. | 70,802 |
| 64 | multiple organ dysfunction syndrome.mp. | 3,567 |
| 65 | multiple organ failure.mp. | 49,489 |
| 66 | cytokine release syndrome.ti,kw. | 1,028 |
| 67 | respiratory distress syndrome/ | 16,277 |
| 68 | acute lung injury/ | 16,603 |
| 69 | adult respiratory distress syndrome/ | 49,425 |
| 70 | transfusion related acute lung injury/ | 2,481 |
| 71 | respiratory distress syndrome.mp. | 86,676 |
| 72 | acute lung injury.mp. | 28,160 |
| 73 | burn/ | 68,006 |
| 74 | burn shock/ | 670 |
| 75 | (burn$ adj3 patient$).ti,kw. | 9,390 |
| 76 | or/42-75 | 1,035,892 |
| 77 | exp ascorbic acid/ | 115,245 |
| 78 | (ascor* or l-ascor* or acidylina* or adenex* or afj c or agrumina* or alle?corb* or antiscorb* or arcavit* c or arkovital* c or ascelat* or ascofar* or ascomed* or asconvita* or askorbin* or austrovit* c or bentavit* c or c crivit* or c ine or c level or c lisa or c long or c monovit* or c prana or c rivitin* or c sol or c tamin* or c tonic or c tron or c vescent or c vicotrat* or c?vimin* or c vit* or c-will or cantan or cantaxin or catavin c or ce arom or ce major or ce quin* or ce?vi?sol or ce vit* or cebetate or cebicure or cebion* or cecap or cecon* or cecorb* or cecrisina or cedon* or cedoxon* or cee-500 or ceevifil or cegiolan or celaskon* or celin* or cenetone or cenol* or cequinyl or cereon or cergona or cescorbat or cetami* or cetebe or ceterapion* or cetrinets or ceva?in* or cevex or cevibram or cevig* or cevilat or cevimin* or cevisol or cevit* or cewin or chewcee or chivibit c or ci drol or ciamin* or ciergin or cifilina or cipca or cisir or cital or citamino or cith or citoascorbina or citoxyl or citran or citravite or citritabs or citrovitamina or civigor or civitin* or co biagini or concemin or cortalex).mp. | 142,048 |
| 79 | (xylo?ascorb* or dagrascorb* or dagravit c or dancimin c or davitamon c or dayvital or delo c or difvitamin c or dumovit* c or erftamin* c or esuron or esurvit* or flavettes or godabion c or gregovite c or hicee or hybrin* or ido c or ikacee or inovitan c or irocevit or irocevite or jarexin* or keto hexuronic acid lactone or lacivit* or laroscorbine or leder?c or lemascorb or limcee or limo ce or liqui cee or magnorbin* or mega-c?a or myascorbin or natrascorb or nybadol or paa 500 or parkovit c or pharmascorb* or pharmatovit* c or planavit* c or plivit* c or pro-c or proscorbin* or redoxon or ribena or scorbacid* or scorbettes or scorbex or scorbin c or scorbitol or scorbumine or scottavit* c or secorbate or sevalin or sigmavit* c or sodascorb* or sweetcee or synum c or take-c or tanvimil-c or testascorb* or ucemine c or upsa?c or vi?ci sin or vi dom c or vi-c 500 or vicef or vicelat or vicetrin or vici monico or viciman or vicin or vicitina or vicon or viforcit* or viscorin* or vita-cedol orange or vitac or vitace or vitacee or vitaci* or vitamin* C or vitaplex c or 4 vitapric or vitapur c or vitasan c or vitascorb* or vitelix c or vitocee or vorange or wandervit* c or witamina c or xitix or xon-ce).mp. | 38,524 |
| 80 | or/77-79 | 149,486 |
| 81 | 41 and 76 and 80 | 477 |

**c) EBM Reviews - Cochrane Central Register of Controlled Trials <May 2022>**

| **#** | **Query** | **Results from 22 Jun 2022** |
| --- | --- | --- |
| 1 | Critical care/ | 1,850 |
| 2 | Critical illness/ | 2,568 |
| 3 | critical care.mp. | 4,684 |
| 4 | intensive care.mp. | 28,438 |
| 5 | critical illness.mp. | 4,120 |
| 6 | critically ill.mp. | 8,315 |
| 7 | Intensive care units/ | 2,621 |
| 8 | exp shock/ | 2,537 |
| 9 | sepsis/ | 2,257 |
| 10 | Shock.ti,kw. | 6,902 |
| 11 | Systemic inflammatory response syndrome.mp. | 1,261 |
| 12 | sepsis.mp. | 13,094 |
| 13 | septic shock.mp. | 3,632 |
| 14 | multiple organ dysfunction syndrome.mp. | 281 |
| 15 | multiple organ failure.mp. | 1,669 |
| 16 | cytokine release syndrome.ti,kw. | 204 |
| 17 | Respiratory Distress Syndrome/ | 1,488 |
| 18 | respiratory distress syndrome.mp. | 6,301 |
| 19 | acute lung injury.mp. | 1,411 |
| 20 | Burns/ | 1,564 |
| 21 | (burn$ adj3 patient$).ti,kw. | 1,046 |
| 22 | or/1-21 | 56,499 |
| 23 | exp Ascorbic Acid/ | 2,337 |
| 24 | (ascor* or l-ascor* or acidylina* or adenex* or afj c or agrumina* or alle?corb* or antiscorb* or arcavit* c or arkovital* c or ascelat* or ascofar* or ascomed* or asconvita* or askorbin* or austrovit* c or bentavit* c or c crivit* or c ine or c level or c lisa or c long or c monovit* or c prana or c rivitin* or c sol or c tamin* or c tonic or c tron or c vescent or c vicotrat* or c?vimin* or c vit* or c-will or cantan or cantaxin or catavin c or ce arom or ce major or ce quin* or ce?vi?sol or ce vit* or cebetate or cebicure or cebion* or cecap or cecon* or cecorb* or cecrisina or cedon* or cedoxon* or cee-500 or ceevifil or cegiolan or celaskon* or celin* or cenetone or cenol* or cequinyl or cereon or cergona or cescorbat or cetami* or cetebe or ceterapion* or cetrinets or ceva?in* or cevex or cevibram or cevig* or cevilat or cevimin* or cevisol or cevit* or cewin or chewcee or chivibit c or ci drol or ciamin* or ciergin or cifilina or cipca or cisir or cital or citamino or cith or citoascorbina or citoxyl or citran or citravite or citritabs or citrovitamina or civigor or civitin* or co biagini or concemin or cortalex).mp. | 6,073 |
| 25 | (xylo?ascorb* or dagrascorb* or dagravit c or dancimin c or davitamon c or dayvital or delo c or difvitamin c or dumovit* c or erftamin* c or esuron or esurvit* or flavettes or godabion c or gregovite c or hicee or hybrin* or ido c or ikacee or inovitan c or irocevit or irocevite or jarexin* or keto hexuronic acid lactone or lacivit* or laroscorbine or leder?c or lemascorb or limcee or limo ce or liqui cee or magnorbin* or mega-c?a or myascorbin or natrascorb or nybadol or paa 500 or parkovit c or pharmascorb* or pharmatovit* c or planavit* c or plivit* c or pro-c or proscorbin* or redoxon or ribena or scorbacid* or scorbettes or scorbex or scorbin c or scorbitol or scorbumine or scottavit* c or secorbate or sevalin or sigmavit* c or sodascorb* or sweetcee or synum c or take-c or tanvimil-c or testascorb* or ucemine c or upsa?c or vi?ci sin or vi dom c or vi-c 500 or vicef or vicelat or vicetrin or vici monico or viciman or vicin or vicitina or vicon or viforcit* or viscorin* or vita-cedol orange or vitac or vitace or vitacee or vitaci* or vitamin* C or vitaplex c or vitapric or vitapur c or vitasan c or vitascorb* or vitelix c or vitocee or vorange or wandervit* c or witamina c or xitix or xon-ce).mp. | 4,247 |
| 26 | or/23-25 | 8,087 |
| 27 | 22 and 26 | 364 |
| 28 | (exp adolescent/ or exp child/ or exp infant/ or (infant disease* or childhood disease*).ti,ab. or (adolescen* or babies or baby or boy? or boyfriend or boyhood or girlfriend or girlhood or child* or girl? or infan* or juvenil* or kid? or minors or minors* or neonat* or neo-nat* or newborn* or new-born* or paediatric* or peadiatric* or pediatric* or perinat* or preschool* or puber* or pubescen* or school* or teen* or toddler? or underage? or under-age? or youth*).ti,ab. or (pediatric* or paediatric* or infan* or child* or adolescen* or young).jn,jw. or (pediatric* or paediatric* or infan* or child* or adolescen* or young).in.) not exp adult/ | 233,004 |
| 29 | 27 not 28 | 327 |

**d) CINAHL**

| S53 | S50 AND S51 AND S52 |
| --- | --- |
| S52 | S42 OR S43 OR S44 OR S45 OR S46 OR S47 |
| S51 | S21 OR S22 OR S23 OR S24 OR S25 OR S26 OR S27 OR S28 OR S29 OR S30 OR S31 OR S32 OR S33 OR S34 OR S35 OR S36 OR S37 OR S38 OR S39 OR S40 OR S41 |
| S50 | S48 NOT S49 |
| S49 | S19 NOT S20 |
| S48 | S1 OR S2 OR S3 OR S4 OR S5 OR S6 OR S7 OR S8 OR S9 OR S10 OR S11 OR S12 OR S13 OR S14 OR S15 |
| S47 | Ascorb* |
| S46 | Vit* C |
| S45 | L-Ascorb* Acid |
| S44 | "Vitamin C" |
| S43 | "Ascorb* Acid" |
| S42 | (MH "Ascorbic Acid") |
| S41 | "burn* patient*" |
| S40 | (MH "Burn Units") OR (MH "Burn Patients") |
| S39 | (MH "Burns") |
| S38 | "acute lung injury" |
| S37 | ""respiratory distress syndrome"" |
| S36 | (MH "Respiratory Distress Syndrome, Acute") OR (MH "Respiratory Distress Syndrome") |
| S35 | ""cytokine release syndrome"" |
| S34 | "septic shock" |
| S33 | "sepsis" |
| S32 | "systemic inflammatory response syndrome" |
| S31 | (MH "Sepsis") OR (MH "Bacteremia") OR (MH "Fungemia") OR (MH "Shock, Septic") |
| S30 | (MH "Cytokine Release Syndrome") OR (MH "Systemic Inflammatory Response Syndrome") |
| S29 | "critical care unit*" |
| S28 | "intensive care unit*" |
| S27 | (MH "Intensive Care Units") OR (MH "Coronary Care Units") OR (MH "Post Anesthesia Care Units") OR (MH "Respiratory Care Units") OR (MH "Stroke Units") |
| S26 | "critically ill" |
| S25 | ""critical illness"" |
| S24 | (MH "Critical Illness") |
| S23 | "intensive care" |
| S22 | "critical care" |
| S21 | (MH "Critical Care") |
| S20 | MH (human) |
| S19 | S16 OR S17 OR S18 |
| S18 | TI (animal model*) |
| S17 | MH (animal studies) |
| S16 | MH animals+ |
| S15 | AB (cluster W3 RCT) |
| S14 | MH (crossover design) OR MH (comparative studies) |
| S13 | AB (control W5 group) |
| S12 | PT (randomized controlled trial) |
| S11 | MH (placebos) |
| S10 | MH (sample size) AND AB (assigned OR allocated OR control) |
| S9 | TI (trial) |
| S8 | AB (random*) |
| S7 | TI (randomised OR randomized) |
| S6 | MH cluster sample |
| S5 | MH pretest‐posttest design |
| S4 | MH random assignment |
| S3 | MH single‐blind studies |
| S2 | MH double‐blind studies |
| S1 | MH randomized controlled trials |

## Table S2: List of excluded studies

| **No** | **Reference** | **Reason** |
| --- | --- | --- |
|  | Bernardo R, Toschi M, Mathew J, Saksouk B, Awab A. 1439: Use Of Vitamin C In Patients With Mild Septic Shock: A Pilot Study. *Crit Care Med*. 2018 Jan 1;46(1):703. | Abstract only |
|  | Fowler AA, Fisher BJ, DeWilde C, Priday A, Syed A, Farthing CA, Larus TL, Knowlson S, Natarajan R. Parenteral vitamin C attenuates markers of organ injury and inflammation in severe sepsis. In A19. Novel Insights And Novel Approaches: Late Breaking Abstracts 2012 May (pp. A6718-A6718). American Thoracic Society. | Abstract only |
|  | Ap GR, Daga MK, Mawari G, Koner BC, Singh VK, Kumar N, Rohatgi I, Mishra R. Effect of Supplementation of Vitamin C and Thiamine on the Outcome in Sepsis: South East Asian Region. The Journal of the Association of Physicians of India. 2022 Mar 1;70(3):11-2. | Abstract only |
|  | Haddaden M, Darweesh M, Haddad I, Haas C. Hydrocortisone, Vitamin C, And Thiamine Supplementation Vs Placebo In Patients With Sepsis. *Chest*. 2021 Oct 1;160(4):A1025. | Abstract only |
|  | Mishra M. 31. Study of High-dose Ascorbic Acid on Vasopressor’s Requirement in Septic Shock Patients: A Surgical Intensive Care Unit Study. *Indian J Crit Care Med* 2020; 24 (Suppl 2): S11. doi: 10.5005/jp-journals-10071-23353.31 | Abstract only |
|  | Rahardjo TM, Redjeki I, Maskoen T. 1119: Effect of Vitamin C 1000 mg IV Therapy To Lactate Level, Base Deficit And SVO2 In Septic Patient. *Crit Care Med*. 2013;41(12):A283. | Abstract only |
|  | Rogobete A, Bedreag O, Cradigati C, Sarandan M, Popovici S, Sandesc D. Influence of antioxidant therapy with high dose of vitamin c on mortality rates in critically ill polytrauma patients. *Crit Care* 2018; 22(Supplement 1):P368. doi: 10.1186/s13054-018-1973-5 | Abstract only |
|  | Rosini JM, Arnold R, Schuchardt BJ, Gissendaner J. Kowalski R. Capan M. High dose intravenous ascorbic acid in severe sepsis. Acad Emerg Med 2018; 25 (Supplement 1): S108. doi: 10.1111/acem.13424 | Abstract only |
|  | Singh R, Bhattacharya S. To evaluate the efficacy of marik protocol in sepsis patient causing circulatory or respiratory compromise or both. *Indian J Crit Care Med* 2012; 25 (Suppl 1):S103. doi: 10.5005/jp-journals-10071-23711.180 | Abstract only |
|  | Fan K, Ronaghi R, Rees J, Baghdasaryan P, Tang J, Lee M, Baydur A. The effect of using vitamin C, hydrocortisone, and thiamine triple therapy in the treatment of septic shock. Chest. 2019 Oct 1;156(4):A944. | Abstract only |
|  | Emadi N, Nemati MH, Ghorbani M, Allahyari E. The Effect of High-Dose Vitamin C on Biochemical Markers of Myocardial Injury in Coronary Artery Bypass Surgery. *Braz J Cardiovasc Surg*. 2019;34(5):517-524. Published 2019 Dec 1. doi:10.21470/1678-9741-2018-0312 | Elective surgical patients |
|  | Rümelin A, Jaehde U, Kerz T, Roth W, Krämer M, Fauth U. Early postoperative substitution procedure of the antioxidant ascorbic acid. *J Nutr Biochem*. 2005;16(2):104-108. doi:10.1016/j.jnutbio.2004.10.005 | Elective surgical patients |
|  | Sadeghpour A, Alizadehasl A, Kyavar M, et al. Impact of vitamin C supplementation on post-cardiac surgery ICU and hospital length of stay. *Anesth Pain Med*. 2015;5(1):e25337. Published 2015 Feb 19. doi:10.5812/aapm.25337 | Elective surgical patients |
|  | Wang D, Wang M, Zhang H, Zhu H, Zhang N, Liu J. Effect of Intravenous Injection of Vitamin C on Postoperative Pulmonary Complications in Patients Undergoing Cardiac Surgery: A Double-Blind, Randomized Trial. *Drug Des Devel Ther*. 2020;14:3263-3270. Published 2020 Aug 11. doi:10.2147/DDDT.S254150 | Elective surgical patients |
|  | Yanase F, Bitker L, Hessels L, et al. A Pilot, Double-Blind, Randomized, Controlled Trial of High-Dose Intravenous Vitamin C for Vasoplegia After Cardiac Surgery. *J Cardiothorac Vasc Anesth*. 2020;34(2):409-416. doi:10.1053/j.jvca.2019.08.034 | Elective surgical patients |
|  | Das D, Sen C, Goswami A. Effect of Vitamin C on adrenal suppression by etomidate induction in patients undergoing cardiac surgery: A randomized controlled trial. *Ann Card Anaesth*. 2016;19(3):410-417. doi:10.4103/0971-9784.185522 | Elective surgical patients |
|  | Duffy MJ, O'Kane CM, Stevenson M, et al. A randomized clinical trial of ascorbic acid in open abdominal aortic aneurysm repair. *Intensive Care Med Exp*. 2015;3(1):50. doi:10.1186/s40635-015-0050-5 | Elective surgical patients |
|  | Rümelin A, Jaehde U, Kerz T, Roth W, Krämer M, Fauth U. Early postoperative substitution procedure of the antioxidant ascorbic acid. *J Nutr Biochem*. 2005;16(2):104-108. doi:10.1016/j.jnutbio.2004.10.005 | Elective surgical patients |
|  | Sadeghpour A, Alizadehasl A, Kyavar M, et al. Impact of vitamin C supplementation on post-cardiac surgery ICU and hospital length of stay. *Anesth Pain Med*. 2015;5(1):e25337. Published 2015 Feb 19. doi:10.5812/aapm.25337 | Elective surgical patients |
|  | Abdoulhossein D, Taheri I, Saba MA, Akbari H, Shafagh S, Zataollah A. Effect of vitamin C and vitamin E on lung contusion: A randomized clinical trial study. *Ann Med Surg (Lond)*. 2018;36:152-157. Published 2018 Nov 9. doi:10.1016/j.amsu.2018.10.026 | No clinical outcome |
|  | Rosengrave PC, Wohlrab C, Spencer E, Williman J, Shaw G, Carr AC. Effect of intravenous vitamin C on arterial blood gas analyser and ACCU-CHEK point-of-care glucose monitoring in critically ill patients. *Crit Care Resusc*. 2022 Jun 1;24(2):175-82. | No clinical outcome |
|  | Tehrani S, Yadegarynia D, Abrishami A, et al. An investigation into the Effects of Intravenous Vitamin C on Pulmonary CT Findings and Clinical Outcomes of Patients with COVID 19 Pneumonia A Randomized Clinical Trial [published online ahead of print, 2021 Nov 8]. *Urol J*. 2021;6863. doi:10.22037/uj.v18i.6863 | Not critically ill patients |
|  | Du WD, Yuan ZR, Sun J, et al. Therapeutic efficacy of high-dose vitamin C on acute pancreatitis and its potential mechanisms. *World J Gastroenterol*. 2003;9(11):2565-2569. doi:10.3748/wjg.v9.i11.2565 | Not critically ill patients |
|  | Kumari P, Dembra S, Dembra P, et al. The Role of Vitamin C as Adjuvant Therapy in COVID-19. *Cureus*. 2020;12(11):e11779. Published 2020 Nov 30. doi:10.7759/cureus.11779 | Not critically ill patients |
|  | Ried K, BinJemain T, Sali A. Therapies to Prevent Progression of COVID-19, Including Hydroxychloroquine, Azithromycin, Zinc, and Vitamin D3 With or Without Intravenous Vitamin C: An International, Multicenter, Randomized Trial. *Cureus*. 2021;13(11):e19902. Published 2021 Nov 25. doi:10.7759/cureus.19902 | Not critically ill patients |
|  | Majidi N, Rabbani F, Gholami S, et al. The Effect of Vitamin C on Pathological Parameters and Survival Duration of Critically Ill Coronavirus Disease 2019 Patients: A Randomized Clinical Trial. *Front Immunol*. 2021;12:717816. Published 2021 Dec 15. doi:10.3389/fimmu.2021.717816 | Not IV Vitamin C |
|  | Raghu D, Ramalingam D. Safety and Efficacy of Vitamin C, Vitamin B1, and Hydrocortisone in clinical outcome of septic shock receiving standard care: A quasi experimental randomized open label two arm parallel group study. *Eur J Mol Clin Med*. 2021 Feb 4;8(2):873-91. | Quasi-trial |
|  | Tanaka H, Matsuda T, Miyagantani Y, Yukioka T, Matsuda H, Shimazaki S. Reduction of resuscitation fluid volumes in severely burned patients using ascorbic acid administration: a randomized, prospective study. *Arch Surg*. 2000;135(3):326-331. doi:10.1001/archsurg.135.3.326 | Quasi-trial |
|  | Tanwar B, Uppal D, Mittal RK, Kaushal S, Garg R, Shah S, Uppal S. To evaluate the role of Vitamin C in reducing the resuscitation fluid volume requirement in burned patients. *Indian J Burns*. 2018 Jan 1;26(1):77. | Quasi-trial |
|  | Nabil Habib T, Ahmed I. Early adjuvant intravenous vitamin C treatment in septic shock may resolve the vasopressor dependence. Int J Microbiol Adv Immunol. 2017 Jul 28;5(1):77-81. | Quasi-trial |
|  | Yoo JW, Kim RB, Ju S, et al. Clinical Impact of Supplementation of Vitamins B1 and C on Patients with Sepsis-Related Acute Respiratory Distress Syndrome. *Tuberc Respir Dis (Seoul)*. 2020;83(3):248-254. doi:10.4046/trd.2020.0008 | Retrospective study |
|  | Ao G, Li J, Yuan Y, et al. Intravenous vitamin C use and risk of severity and mortality in COVID-19: A systematic review and meta-analysis. *Nutr Clin Pract*. 2022;37(2):274-281. doi:10.1002/ncp.10832 | Systematic review |
|  | Assouline B, Faivre A, Verissimo T, et al. Thiamine, Ascorbic Acid, and Hydrocortisone As a Metabolic Resuscitation Cocktail in Sepsis: A Meta-Analysis of Randomized Controlled Trials With Trial Sequential Analysis. *Crit Care Med*. 2021;49(12):2112-2120. doi:10.1097/CCM.0000000000005262 | Systematic review |
|  | Du X, Yang C, Yu X. [Effect of vitamin C on prognosis of critically ill patients: a Meta-analysis] *Zhonghua Wei Zhong Bing Ji Jiu Yi Xue*. 2019;31(8):942-948. doi:10.3760/cma.j.issn.2095-4352.2019.08.006 | Systematic review |
|  | Feng F, Yang H, Yang W, Li M, Chang X, Chen Y. Effect of vitamin C in critically ill patients with sepsis and septic shock: A meta-analysis. *Sci Prog*. 2021;104(1):36850421998175. doi:10.1177/0036850421998175 | Systematic review |
|  | Fong KM, Au SY, Ng GWY. Steroid, ascorbic acid, and thiamine in adults with sepsis and septic shock: a systematic review and component network meta-analysis. *Sci Rep*. 2021;11(1):15777. Published 2021 Aug 4. doi:10.1038/s41598-021-95386-9 | Systematic review |
|  | Fujii T, Salanti G, Belletti A, et al. Effect of adjunctive vitamin C, glucocorticoids, and vitamin B1 on longer-term mortality in adults with sepsis or septic shock: a systematic review and a component network meta-analysis. *Intensive Care Med*. 2022;48(1):16-24. doi:10.1007/s00134-021-06558-0 | Systematic review |
|  | Ge Z, Huang J, Liu Y, Xiang J, Gao Y, Walline JH, Lu X, Yu S, Zhao L, Li Y. Thiamine combined with vitamin C in sepsis or septic shock: A systematic review and meta-analysis. *Eur J Emerg Med*. 2021 Jun 1;28(3):189-95. | Systematic review |
|  | Hemilä H, Chalker E. Vitamin C may reduce the duration of mechanical ventilation in critically ill patients: a meta-regression analysis. *J Intensive Care*. 2020;8:15. Published 2020 Feb 7. doi:10.1186/s40560-020-0432-y | Systematic review |
|  | Langlois PL, Manzanares W, Adhikari NKJ, et al. Vitamin C Administration to the Critically Ill: A Systematic Review and Meta-Analysis. *JPEN J Parenter Enteral Nutr*. 2019;43(3):335-346. doi:10.1002/jpen.1471 | Systematic review |
|  | Li T, Zeng J, Li DH, et al. Efficacy of intravenous vitamin C intervention for septic patients: A systematic review and meta-analysis based on randomized controlled trials. *Am J Emerg Med*. 2021;50:242-250. doi:10.1016/j.ajem.2021.08.012 | Systematic review |
|  | Patel JJ, Ortiz-Reyes A, Dhaliwal R, et al. IV Vitamin C in Critically Ill Patients: A Systematic Review and Meta-Analysis. *Crit Care Med*. 2022;50(3):e304-e312. doi:10.1097/CCM.0000000000005320 | Systematic review |
|  | Putzu A, Daems AM, Lopez-Delgado JC, Giordano VF, Landoni G. The Effect of Vitamin C on Clinical Outcome in Critically Ill Patients: A Systematic Review With Meta-Analysis of Randomized Controlled Trials. *Crit Care Med*. 2019;47(6):774-783. doi:10.1097/CCM.0000000000003700 | Systematic review |
|  | Sato R, Hasegawa D, Prasitlumkum N, et al. Effect of IV High-Dose Vitamin C on Mortality in Patients With Sepsis: A Systematic Review and Meta-Analysis of Randomized Controlled Trials. *Crit Care Med*. 2021;49(12):2121-2130. doi:10.1097/CCM.0000000000005263 | Systematic review |
|  | Scholz SS, Borgstedt R, Ebeling N, Menzel LC, Jansen G, Rehberg S. Mortality in septic patients treated with vitamin C: a systematic meta-analysis. *Crit Care*. 2021;25(1):17. Published 2021 Jan 6. doi:10.1186/s13054-020-03438-9 | Systematic review |
|  | Shokri-Mashhadi N, Aliyari A, Hajhashemy Z, Saadat S, Rouhani MH. Is it time to reconsider the administration of thiamine alone or in combination with vitamin C in critically ill patients? A meta-analysis of clinical trial studies. *J Intensive Care*. 2022;10(1):8. Published 2022 Feb 17. doi:10.1186/s40560-022-00594-8 | Systematic review |
|  | Shrestha DB, Budhathoki P, Sedhai YR, et al. Vitamin C in Critically Ill Patients: An Updated Systematic Review and Meta-Analysis. *Nutrients*. 2021;13(10):3564. Published 2021 Oct 12. doi:10.3390/nu13103564 | Systematic review |
|  | Somagutta MKR, Pormento MKL, Khan MA, et al. The Efficacy of vitamin C, thiamine, and corticosteroid therapy in adult sepsis patients: a systematic review and meta-analysis. *Acute Crit Care*. 2021;36(3):185-200. doi:10.4266/acc.2021.00108 | Systematic review |
|  | Xing X, Xu M, Yang L, Zhang W, Niu X, Gao D. The efficacy of intravenous vitamin C in critically ill patients: A meta-analysis of randomized controlled trials. *Clin Nutr*. 2021;40(5):2630-2639. doi:10.1016/j.clnu.2021.03.007 | Systematic review |
|  | Yao R, Zhu Y, Yu Y, et al. Combination therapy of thiamine, vitamin C and hydrocortisone in treating patients with sepsis and septic shock: a meta-analysis and trial sequential analysis. *Burns Trauma*. 2021;9:tkab040. Published 2021 Dec 6. doi:10.1093/burnst/tkab040 | Systematic review |
|  | Zayed Y, Alzghoul BN, Banifadel M, et al. Vitamin C, Thiamine, and Hydrocortisone in the Treatment of Sepsis: A Meta-Analysis and Trial Sequential Analysis of Randomized Controlled Trials. *J Intensive Care Med*. 2022;37(3):327-336. doi:10.1177/0885066620987809 | Systematic review |
|  | Zhang M, Jativa DF. Vitamin C supplementation in the critically ill: A systematic review and meta-analysis. *SAGE Open Med*. 2018;6:2050312118807615. Published 2018 Oct 19. doi:10.1177/2050312118807615 | Systematic review |
|  | ﻿Agarwal A, Basmaji J, Fernando SM, et al. Parenteral Vitamin C in Patients with Severe Infection: A Systematic Review. *NEJM Evidence*. 2022. doi:10.1056/EVIDoa2200105 | Systematic review |
|  | Ammar MA, Ammar AA, Condeni MS, Bell CM. Vitamin C for Sepsis and Septic Shock. *Am J Ther*. 2021;28(6):e649-e679. Published 2021 Jul 5. doi:10.1097/MJT.0000000000001423 | Systematic review |
|  | Hemilä H, Chalker E. Vitamin C Can Shorten the Length of Stay in the ICU: A Meta-Analysis. *Nutrients*. 2019;11(4):708. Published 2019 Mar 27. doi:10.3390/nu11040708 | Systematic review |
|  | Na W, Shen H, Li Y, Qu D. Hydrocortisone, ascorbic acid, and thiamine (HAT) for sepsis and septic shock: a meta-analysis with sequential trial analysis. *J Intensive Care*. 2021;9(1):75. Published 2021 Dec 18. doi:10.1186/s40560-021-00589-x | Systematic review |
|  | Wei XB, Wang ZH, Liao XL, et al. Efficacy of vitamin C in patients with sepsis: An updated meta-analysis. *Eur J Pharmacol*. 2020;868:172889. doi:10.1016/j.ejphar.2019.172889 | Systematic review |
|  | Wu T, Hu C, Huang W, Xu Q, Hu B, Li J. Effect of Combined Hydrocortisone, Ascorbic Acid and Thiamine for Patients with Sepsis and Septic Shock: A Systematic Review and Meta-Analysis. *Shock*. 2021;56(6):880-889. doi:10.1097/SHK.0000000000001781 | Systematic review |
|  | Balakrishnan M, Gandhi H, Shah K, et al. Hydrocortisone, Vitamin C and thiamine for the treatment of sepsis and septic shock following cardiac surgery. *Indian J Anaesth*. 2018;62(12):934-939. doi:10.4103/ija.IJA_361_18 | Vitamin C combination therapy |
|  | Chang P, Liao Y, Guan J, et al. Combined Treatment With Hydrocortisone, Vitamin C, and Thiamine for Sepsis and Septic Shock: A Randomized Controlled Trial. *Chest*. 2020;158(1):174-182. doi:10.1016/j.chest.2020.02.065 | Vitamin C combination therapy |
|  | Fujii T, Luethi N, Young PJ, et al. Effect of Vitamin C, Hydrocortisone, and Thiamine vs Hydrocortisone Alone on Time Alive and Free of Vasopressor Support Among Patients With Septic Shock: The VITAMINS Randomized Clinical Trial. *JAMA*. 2020;323(5):423-431. doi:10.1001/jama.2019.22176 | Vitamin C combination therapy |
|  | Hussein AA, Sabry NA, Abdalla MS, Farid SF. A prospective, randomised clinical study comparing triple therapy regimen to hydrocortisone monotherapy in reducing mortality in septic shock patients. *Int J Clin Pract*. 2021;75(9):e14376. doi:10.1111/ijcp.14376 | Vitamin C combination therapy |
|  | Hwang SY, Ryoo SM, Park JE, et al. Combination therapy of vitamin C and thiamine for septic shock: a multi-centre, double-blinded randomized, controlled study. *Intensive Care Med*. 2020;46(11):2015-2025. doi:10.1007/s00134-020-06191-3 | Vitamin C combination therapy |
|  | Iglesias J, Vassallo AV, Patel VV, Sullivan JB, Cavanaugh J, Elbaga Y. Outcomes of Metabolic Resuscitation Using Ascorbic Acid, Thiamine, and Glucocorticoids in the Early Treatment of Sepsis: The ORANGES Trial. *Chest*. 2020;158(1):164-173. doi:10.1016/j.chest.2020.02.049 | Vitamin C combination therapy |
|  | Jamshidi MR, Zeraati MR, Forouzanfar B, Tahrekhani M, Motamed N. Effects of triple combination of hydrocortisone, thiamine, and Vitamin C on clinical outcome in patients with septic shock: A single-center randomized controlled trial. *J Res Med Sci*. 2021;26:47. Published 2021 Jul 31. doi:10.4103/jrms.JRMS_593_19 | Vitamin C combination therapy |
|  | Mohamed ZU, Prasannan P, Moni M, et al. Vitamin C Therapy for Routine Care in Septic Shock (ViCTOR) Trial: Effect of Intravenous Vitamin C, Thiamine, and Hydrocortisone Administration on Inpatient Mortality among Patients with Septic Shock. *Indian J Crit Care Med*. 2020;24(8):653-661. doi:10.5005/jp-journals-10071-23517 | Vitamin C combination therapy |
|  | Moskowitz A, Huang DT, Hou PC, et al. Effect of Ascorbic Acid, Corticosteroids, and Thiamine on Organ Injury in Septic Shock: The ACTS Randomized Clinical Trial. *JAMA*. 2020;324(7):642-650. doi:10.1001/jama.2020.11946 | Vitamin C combination therapy |
|  | Nathens AB, Neff MJ, Jurkovich GJ, et al. Randomized, prospective trial of antioxidant supplementation in critically ill surgical patients. *Ann Surg*. 2002;236(6):814-822. doi:10.1097/00000658-200212000-00014 | Vitamin C combination therapy |
|  | Reddy PR, Samavedam S, Aluru N, Yelle S, Rajyalakshmi B. Metabolic Resuscitation Using Hydrocortisone, Ascorbic Acid, and Thiamine: Do Individual Components Influence Reversal of Shock Independently?. *Indian J Crit Care Med*. 2020;24(8):649-652. doi:10.5005/jp-journals-10071-23515 | Vitamin C combination therapy |
|  | Hesham El-Sherazy N, Samir Bazan N, Mahmoud Shaheen S, A Sabri N. Impact of ascorbic acid in reducing the incidence of vancomycin associated nephrotoxicity in critically ill patients: A preliminary randomized controlled trial. *F1000Res*. 2021;10:929. Published 2021 Sep 16. doi:10.12688/f1000research.55619.1 | Vitamin C combination therapy |
|  | Sevransky JE, Rothman RE, Hager DN, et al. Effect of Vitamin C, Thiamine, and Hydrocortisone on Ventilator- and Vasopressor-Free Days in Patients With Sepsis: The VICTAS Randomized Clinical Trial [published correction appears in JAMA. 2021 Sep 21;326(11):1072]. *JAMA*. 2021;325(8):742-750. doi:10.1001/jama.2020.24505 | Vitamin C combination therapy |
|  | Tsai SC, Liu CT, Liao HH, Lin FC. Effects of Enteral Glutamine and Vitamin C Supplementation on Cytokines and Outcomes in Surgical Intensive Care Unit Patients: A Randomized Clinical Trial. *Zhong shan yi xue za zhi*. 2020 Dec 1;31(2):101-14. | Vitamin C combination therapy |
|  | Aisa-Alvarez A, Soto ME, Guarner-Lans V, et al. Usefulness of Antioxidants as Adjuvant Therapy for Septic Shock: A Randomized Clinical Trial. *Medicina (Kaunas)*. 2020;56(11):619. Published 2020 Nov 17. doi:10.3390/medicina56110619 | Vitamin C combination therapy |
|  | Bansal D, Bhalla A, Bhasin DK, et al. Safety and efficacy of vitamin-based antioxidant therapy in patients with severe acute pancreatitis: a randomized controlled trial. *Saudi J Gastroenterol*. 2011;17(3):174-179. doi:10.4103/1319-3767.80379 | Vitamin C combination therapy |
|  | Darban M, Malek F, Memarian M, Gohari A, Kiani A, Emadi A, Lavvaf S, Bagheri B. Efficacy of high dose vitamin C, melatonin and zinc in Iranian patients with acute respiratory syndrome due to coronavirus infection: a pilot randomized trial. *J Cell Mol Anesth.* 2021;6(2):164-7. | Vitamin C combination therapy |
|  | Galley HF, Howdle PD, Walker BE, Webster NR. The effects of intravenous antioxidants in patients with septic shock. Free Radic. Biol. Med. 1997 Jan 1;23(5):768-74. | Vitamin C combination therapy |
|  | Karimpour H, Bahrami A, Amini S, Rezaei M, Amini-Saman J, Shahbazi F. Effects of a high dose of vitamin C along with thiamine in critically-ill patients with septic shock: a preliminary study. J Pharm Res Int. 2019;29(5):1-7. | Vitamin C combination therapy |
|  | Lubis AP, Sugiarto A, Madjid AS. Effect of Vitamin C and Vitamin B1 Combination on Mortality of Sepsis and Septic Shock Patients in Intensive Care Unit. International Journal of Health Sciences. 2019 Dec;7(4):9-12. | Vitamin C combination therapy |
|  | Wani SJ, Mufti SA, Jan RA, et al. Combination of vitamin C, thiamine and hydrocortisone added to standard treatment in the management of sepsis: results from an open label randomised controlled clinical trial and a review of the literature. *Infect Dis (Lond)*. 2020;52(4):271-278. doi:10.1080/23744235.2020.1718200 | Vitamin C combination therapy |
|  | Yadav AK, Singh VK, Singh G, Singh V. Outcome of Ulinastatin vs Metabolic Resuscitation using Ascorbic Acid, Thiamine and Glucocorticoid in Early Treatment of Sepsis-A Randomised Controlled Trial. *J Clin Diagn Res*. 2021 May 1;15(5). | Vitamin C combination therapy |

## Table S3: Critical Care Nutrition Methodological System

| **No** | **Author, year (country)** | **Concealed Randomization** | **Intention-to-treat**  **Analysis** | **Blinding** | **Patient**  **Selection** | **Comparability**  **of groups at**  **baseline** | **Extent of**  **Follow-up** | **Description**  **of treatment**  **protocol** | **Description**  **of treatment**  **co-interventions** | **Objectivity**  **of the definition**  **of outcomes** | **Total score**  **(max 14)** |
| --- | --- | --- | --- | --- | --- | --- | --- | --- | --- | --- | --- |
| 1 | Ferron-Celma 2008 (Spain) | 1 | 2 | 2 | 0 | 1 | 1 | 1 | 0 | 1 | **9** |
| 2 | Razmkon 2011 (Iran) | 1 | 2 | 2 | 0 | 0 | 1 | 0 | 1 | 1 | **8** |
| 3 | Fowler 2014 (USA) | 2 | 0 | 2 | 0 | 0 | 0 | 1 | 0 | 2 | **7** |
| 4 | Zabet 2016 (Iran) | 1 | 2 | 2 | 0 | 1 | 1 | 1 | 1 | 2 | **11** |
| 5 | Chen 2019 (China) | 1 | 0 | 1 | 0 | 1 | 1 | 1 | 1 | 2 | **8** |
| 6 | Fowler 2019  (USA) | 2 | 0 | 2 | 0 | 1 | 1 | 1 | 1 | 2 | **10** |
| 7 | Niu 2019 (China) | 1 | 2 | 0 | 0 | 1 | 1 | 1 | 1 | 2 | **9** |
| 8 | Lv 2020 (China) | 1 | 2 | 0 | 0 | 1 | 1 | 1 | 1 | 1 | **8** |
| 9 | Jamali Moghadam Siahkali 2021 (Iran) | 1 | 2 | 0 | 0 | 1 | 1 | 1 | 1 | 1 | **8** |
| 10 | Kassem 2021  (Egypt) | 1 | 2 | 1 | 1 | 0 | 1 | 1 | 0 | 2 | **9** |
| 11 | Mahmoodpoor 2021 (Iran) | 1 | 0 | 2 | 1 | 0 | 1 | 0 | 1 | 2 | **8** |
| 12 | Zhang 2021 (China) | 2 | 2 | 2 | 0 | 1 | 1 | 1 | 1 | 2 | **12** |
| 13 | Ap 2022 (India) | 1 | 2 | 0 | 0 | 0 | 1 | 0 | 0 | 1 | **5** |
| 14 | Rosengrave 2022 (New Zealand) | 2 | 2 | 2 | 0 | 1 | 1 | 1 | 0 | 2 | **11** |
| 15 | Wacker 2022 (USA) | 1 | 0 | 2 | 1 | 0 | 1 | 1 | 1 | 2 | **9** |
| 16 | Lamontagne 2022 (International) | 2 | 0 | 2 | 1 | 1 | 1 | 1 | 2 | 2 | **12** |

Median score=9

## Table S4: The Intervention

| **Author, year (country)** | **Control group** | **Vitamin C Gp 1** | **Vitamin C Gp 2** | **Method of administration** | **Start of intervention** | **Duration of Intervention** | **Total Vit C received per day in a 70kg patient** |
| --- | --- | --- | --- | --- | --- | --- | --- |
| Ferron-Celma 2008 (Spain) | Placebo: D5% | 450 mg/d in D5% | N/A | divided into 3 doses | Enrolled patients at 12 h post abdominal surgery | 6 days | 450 mg |
| Razmkon 2011 (Iran) | Placebo: unspecified | 500 mg/d | Admission and day 4: 10000 mg  Next 3 days: 4000 mg/d | Unclear | Enrolled patients ≤8 h of head trauma | 7 days | Gp 1: 500 mg  Gp 2: 10000 or 4000 mg |
| Fowler 2014 (USA) | Placebo: D5% | 50 mg/kg/d in 50 ml D5% | 200 mg/kg/d in 50 ml D5% | divided into 4 equal doses, administered over 30 minutes every 6 h | Enrolled patients diagnosed with sepsis ≤48 h of ICU admission | up to 96 h | Gp 1: 3500 mg  Gp 2: 14000 mg |
| Zabet 2016 (Iran) | Placebo: D5% | 25 mg/kg every 6 h in 50ml D5% | N/A | administered over 30 minutes | Enrolled sepsis patients - ICU admission to a diagnosis of sepsis: 96.43±22.78h | up to 72 h | 7000 mg |
| Chen 2019 (China) | Placebo: normal saline dilutes in 30ml D5% | 50 mg/kg/d in 30 ml D5% | 150 mg/kg/d in 30 ml D5% | divided into 4 equal doses, administered over 30 minutes, given 4 times/d | ≤96 h of randomization | 96 h* | Gp 1: 3500 mg  Gp 2: 10500 mg |
| Fowler 2019  (USA) | Placebo: D5% | 50 mg/kg every 6 h in 50 ml D5% | N/A | administered over 30 minutes | Randomized ≤24 h of ICU admission or ≤48 h of ARDS. Start within 6 h of randomization | up to 96 h | 14000 mg |
| Niu 2019 (China) | Placebo: D5% | 3000 mg in D5% | N/A | Unclear | On the day of ICU admission | until ICU discharge or dead (ICU LOS ~4 days) | 3000 mg |
| Lv 2020 (China) | Placebo: D5% | 3000 mg in 100ml D5%, 2 times/d | N/A | Unclear (administered daily at 8 am and 8 pm) | On the day of ICU admission | until ICU discharge (ICU LOS ~4 days) | 6000 mg |
| Jamali Moghadam Siahkali 2021 (Iran) | Usual care- no placebo | 1500 mg every 6 h | N/A | Unclear | Unclear | up to 5 days | 6000 mg |
| Kassem 2021  (Egypt) | Placebo: normal saline | 2500 mg every 6h | N/A | Unclear (using an infusion set) | Unclear | up to 96 h | 10000 mg |
| Mahmoodpoor 2021 (Iran) | Placebo: normal saline | 60 mg/kg/d | N/A | Continuous infusion | Unclear | up to 96 h | 4200 mg |
| Zhang 2021 (China) | Placebo: Sterile water | 12000 mg diluted in 50 ml sterile water every 12 h, 2X/d | N/A | infused at a rate of 12 ml/h | Randomized within 48 h of ICU admission. Start within the same day of randomization | up to 7 days | 24000 mg |
| Ap 2022 (India) | Usual care- no placebo | 2000 mg in D5% of normal saline, 3 times/d | N/A | administered over 30-60 minutes | Unclear | 5 days | 6000 mg |
| Rosengrave 2022 (New Zealand) | Placebo: D5% | 25 mg/kg in 50 ml D5% every 6 h | N/A | administered over 30 minutes | ICU admission to randomization: 17 (12-25) h; randomization to first treatment: 45 (33-73) minutes | up to 96 h | 7000 mg |
| Wacker 2022 (USA) | Placebo: Normal saline | 1000 mg bolus, then continuous 250 mg/h | N/A | bolus over 30 minutes, then continuous infusion | Enrolled patients ≤24 h of septic shock and initiated study drug ≤24 h of eligibility. Time from pressor initiation to study drug initiation (h): 11.4 (5.4-17.3) vs 8.8 (5.4-17.4) | up to 96 h or vasopressor-free for 24 consecutive hours | 7000 mg |
| Lamontagne 2022 (International) | Placebo: D5% or normal saline | 50 mg/kg actual body weight in 50 ml D5% or normal saline, every 6 h | N/A | administered over 30-60 minutes | ICU admission to randomization: 12.9±8.2 vs 12.3±6.7 h | up to 96 h | 14000 mg |

**ARDS: acute respiratory distress syndrome, D5%: dextrose 5%, Gp: group, LOS: length of stay**

***Chen 2019: if treatment was discontinued before 96h due to factors such as arrythmia, dialysis or dead of the patient, the patient will be excluded from the analysis**

## Table S5: Outcomes summary

| **No** | **Author year (country)** | **Mortality n(%)** | **Infections n( %)** | **Duration of mechanical ventilation and length of stays** | **Other outcomes** |
| --- | --- | --- | --- | --- | --- |
| 1 | Ferron-Celma 2008 (Spain) | **Unspecified**  6/10 (60) vs 4/10 (40) | NR | NR | NR |
| 2 | Razmkon 2011 (Iran) | **Hospital**  7/26 (26.9) vs 7/23 (30.4) vs 8/27 (29.7); p=NR  **60 day**  8/26 (30.8) vs 7/23 (30.4) vs 8/27 (29.7); p=NR  **6 month**  9/26 (34.6) vs 7/23 (30.4) vs 8/27 (29.7); p=NR | NR | **Hospital LOS**  All patients:15.2 ±4.3 (beween group p=0.08) | NR |
| 3 | Fowler 2014 (USA) | **28-day**  3/8 (38.1) vs. 4/8 (50.6) vs. 5/8 (62.5); p=NR | NR | **Ventilator-free days**  8.4 (0-22) vs 4.8 (0-19) vs 7.6 (0-23); p=NR  **ICU LOS**  8.1 (1-19) vs 9.1 (2-25) vs11 (2-25); p=NR | **Days on Pressors**  2.1(1-6) vs 3.6 (2-8) vs 3.9 (1-10); p=NR  **Change in SOFA score at day 4**:  -0.020 vs. -0.043 vs. 0.003 (high-dose vs placebo p<0.01) |
| 4 | Zabet 2016 (Iran) | **28-day**  2/14 (14) vs 9/14 (64); p=0.009 | NR | **ICU LOS**  21.45±10.23 (14) vs 20.57±13.04 (14); p=0.85 | **Mean dose of noradrenaline (ug/min) during 72h study period**  7.44±3.65 vs 13.79±6.48; p=0.004  **Duration of noradrenaline administration (h)**  49.64±25.67 vs 71.57±1.60; p=0.007  (2.07±1.07 vs 2.98±0.07 days) |
| 5 | Chen 2019 (China) | **28-day**  10/41 (24.4) vs 4/39 (10.3) vs 14/42 (33.3); p=0.046 | NR | **Duration of MV**  12.5±12.1 (41) vs 13.2±13.9 (39) vs 12.8±14.0 (42); p=0.979  Pooled: 12.84±13.01(80)  **ICU LOS**  19.5±11.6 (41) vs 19.1±14.4 (39) vs 19.7±14.9 (42); p=0.979  Pooled:19.31±13.04(80)  **Hosp LOS**  23.2±15.2 (41) vs 22.8±16.2 (39) vs 22.6±16.4 (42); p=0.987  Pooled:23.06±15.70(80) | **Change in SOFA score at 72h**: -0.15±0.24 (41) vs -0.29±0.25 (39) vs -0.05±0.31 (42); p<0.001  **Change in SOFA score at 96h**: -0.14±0.29 (41) vs -0.34±0.25 (39) vs -0.03±0.38 (42); p<0.001  **SOFA score at 72h**: 4.92±2.72 vs 5.37±2.75 vs 6.02±3.41; p=0.250  **SOFA score at 96h**: 4.56±2.64 (41) vs 5.44±3.19 (39) vs 6.17±3.74 (42); p=0.088 [pooled Vit C: 4.99±2.92 (80)]  **Time on norad (h)**: 80±27.3 (18) vs 72±28.1 (16) vs 84.6±19.5 (21); p=0.328  (3.33±1.14 vs 3±1.17 vs 3.53±0.81 days) [pooled Vit C: 3.17±1.15 (n=34)]  **Average 96h norad dose (ug/min)**: 26.0±39.2 (18) vs 15.5±17.6 (16) vs 22.5±23.0 (21); p=0.555  (pooled: 21.06±31.01 (34) vs 22.5±23.0 (21))7 |
| 6 | Fowler 2019  (USA) | **28-day**  25/84 (29.8) vs 38/82 (46.3); p=0.03  **60-day***  28/84 (33.3) vs 38/82 (46.9) | NR | **Ventilator-free days to day 28**  13.1 vs 10.6; p=0.15  **ICU LOS***  10.4±6.2 (84) vs 12.3±5.7 (82)  **Hospital-free days to day 60**  22.6 vs 15.5 days; p=0.04 | **Change in mSOFA score at 96h**  -3 (9.8 to 6.8) vs -3.5 (10.4-6.8) (mean difference: -0.10; 95% CI -1.23 to 1.03; p=0.86)  **Vasopressor use at 96h**  24/80 (30.0) vs 18/65 (27.7) |
| 7 | Niu 2019 (China) | **28-d**  34/122 (27.9) vs 48/112 (42.9); p=0.016 | 19/122 (15.6) vs 18/112 (16.1) | **Duration of MV**  NR (62/22 vs 56/112 were intubated)  **ICU LOS**  4.0 (3.0-8.0) (122) vs 4.0 (3.0-7.3) (112); p=0.812 | **Change in SOFA score at 72h**  4.0 (1.0-6.0) vs 2.0 (1.0-4.0); p<0.001  **Time on vasopressor (h)**  25.0 (18.0-40.0) vs 43.0 (24.0-66.0); p<0.001  (1.04 (0.75-1.67) vs 1.79 (1.0-2.75) days) |
| 8 | Lv 2020 (China) | **28-day**  15/61 (24.6) vs 24/56 (42.9), p=0.002 | NR | **ICU LOS**  4.1 (3.2-8.3) vs 3.9 (3.1-7.5); p=0.811 | **SOFA score at 72h (assumed to be the changed of SOFA score at 72h)#**  4.2 (1.2-6.6) vs 2.1 (1.1-4.3); p=0.001  **Time on vasoactive drugs, h**  25.6 (18.8-40.6) vs 43.8 (24.7-66.8); p=0.001  (1.07 (0.78-1.69) vs 1.83 (1.03-2.78) days) |
| 9 | Jamali Moghadam Siahkali 2021 (Iran) | **Unspecifed**  3/30 (10) vs 3/30 (10) | NR | **Duration of MV**  NR (5/30 vs 4/30 were intubated)  **ICU LOS**  5.5 (5-10) (30) vs 5 (5-7) (30); p=0.381  **Hosp LOS**  8.5 (7-12) (30) vs 6.5 (4-12) (30); p=0.028 | NR |
| 10 | Kassem 2021  (Egypt) | **7-d**  6/40 (15) vs 17/40 (42.5); p=0.013  **28-d**  13/40 (32.5) vs 20/40 (50.0); p=0.173 | NR | **Duration of MV**  4 (IQR 3) vs 5 (IQR 3); p=0.611  **ICU LOS** 8 (IQR 6) vs 7 (IQR 6); p=0.649 | **Duration of Vasopressor (days)**  1 (IQR 4) vs 4 (IQR 5); p=0.019  **AKI**: 4/40 vs 5/40 |
| 11 | Mahmoodpoor 2021 (Iran) | **28-d**  6/40 (15.0) vs 11/40 (27.5); p=0.17 | NR | **Duration of MV** 4.05±2.29 (40) vs 8.92±2.96 (40); p<0.001  **ICU LOS**  12.77±3.71 (40) vs 14.15±3.12 (40); p=0.07 | **Duration of Vasopressor (days)**  2.28±1.24 (40) vs 3.39±1.23 (40); p=0.003  **Vasopressor dose (ug/min)**  6.8±3.18 (40) vs 8.26±3.58 (40); p=0.14  **SOFA score at 96h**: 1.62±1.19 (40) vs 3.10±1.61 (40); p<0.001  **AKI**: 16/40 VS 22/40 |
| 12 | Zhang 2021 (China) | **ICU**  6/27 (22.2%) vs 11/29 (37.9%); p=0.20  **Hospital**  6/27 (22.2%) vs 11/29 (37.9%); p=0.20  **28 day**  6/27 (22.2%) vs 10/29 (34.5%), p=0.31  **ICU mortality**  **(in subgroup SOFA ≥ 3)**  5/27(21.7%) vs 11/29 (52.4%), p=0.04 | NR | **Duration of MV to day 28**  1.5 [0.0-19.0] vs 6.0 [0.0–16.0]; p=0.60  **ICU LOS**  22.9±14.8 vs 17.8±13.3; p=0.20  **Hosp LOS**  35.0±17.0 vs 32.8±17.0, p=0.65 | **Median SOFA score change Day 1-7**: 0 [-2.75 to 1] vs 0 [-1 to -3.5]; p=0.25  **AKI**: 3/27 VS 6/29  **CRRT on day 7**: 3/27 vs 1/29 |
| 13 | Ap 2022 (India) | **Unspecified**  2/20 (20) vs 1/20 (5); p=0.548 | NR | NR | **Change in SOFA score from day 1-6**  4.70±2.47 vs 3.85±1.79 (p-value not reported) |
| 14 | Rosengrave 2022 (New Zealand) | **30-d**  6/20 (30) vs 7/20 (35)  **90-d**  8/20 (40) vs 7/20 (35)  **Hospital**  7/20 (35) vs 7/20 (35) | NR | **ICU LOS (survivor)**  3.8 (2.2-9.8) vs 7.1 (3.1-20); p=0.12  6.38±6.21 (16) vs 10.71±14.25 (14)*  **Hosp LOS (survivor)**  18 (11-35) vs 22 (10-52); p=0.65  19.46±13.58 (12) vs 28.71±27.13 (13)* | **Mean dose of vaopressor (units/min):** 0.99±0.55 (20) vs 0.71±0.60 (20); p=0.35  **Mean duration of vasopressor (h):** 48 (95% CI 35-62) vs 54 (95% CI 41-62); p=0.54  (2 (95% CI 1.46 to 2.58) vs 2.25 (95% CI 1.71-2.58) days)  **SOFA score at 96h:** 6.7±8.3 (n=20) vs 5.5±7.0; p=0.64 (n=20) |
| 15 | Wacker 2022 (USA) | **28 day**  16/60 (26.7) vs 26/64 (40.6); p=0.10  **ICU**  14/60 (23.3) vs 20/64 (31.1); p=0.32 | NR | **Duration of MV**  0 (0-60) (60) vs 5 (0-48) (64); p=0.45  **ICU LOS**  2.9 (1.8-7.5) (60) vs 2.6 (1.5-5.3) (64); p=0.47  **Hosp LOS**  8.9 (4.0-20.0) (60) vs 6.3 (3.8-12.5) (64); p=0.15 | **Improvement in SOFA score at 96h**  3.5 (1-6) (n=58) vs 4 (1-6) (n=61); p=0.68  **Duration of pressors after initiation of study drug (h)**  27.7 (13.6-47.6) (n=60) vs 27.1 (16.4-45.2) (n=64); p=0.79  (1.15 (0.57-1.98) vs 1.13 (0.68-1.88) days)  **Incidence of RRT during 96h study period**  10/60 (16.7) vs 2/60 (3.3); p=0.02 |
| 16 | Lamontagne 2022 (International) | **28-d**  152/429 (35.4) vs 137/434 (31.6)  **6-mo**  191/417 (45.8) vs 185/426 (43.4) | NR | **Duration of MV (all patients)***  8.4±7.6 (306) vs 8.1±7.5 (296)  **ICU LOS**  6 (3-12) (429) vs 6 (3-11) (433)  10.4±13.6 (429) vs 9.5±11.3 (433)*  **Hosp LOS**  15 (8-32) (429) vs 16 (8-33) (433)  27.2±32 (429) vs 28.5±41.2 (433)* | **6-mo QOL by EQ-5D-5L**  65.8±20.9 (n=222) vs 63.8±22.5 (n=233)  **Day 3 SOFA score**  9.2±6.0 (n=429) vs 9.0±6.0 (n=432) (risk ratio 0.23 [95% CI: −0.57 to 1.03]  **Day 4 SOFA score**  8.7±6.5 (n=429) vs 8.7±6.6 (n=432) (risk ratio −0.03 [95%CI: −0.90 to 0.85])  **Days on vasopressors**  - Survivors: 3 (2-5) (n=298) vs 3 (2-5) (n=312); not sig  - Non-survivrs: 4 (2-8) (n=131) vs 4 (2-9) (n=121); not sig  **Incidence of RRT**: 109/429 vs 112/433  **Incidence of AKi:** 162/429 vs 164/433 |

The results are presented in this sequence: intervention (low-dose vs high dose) vs control

AEs: adverse events, AKI: acute kidney injury, ICU: intensive care unit, LOS: length of stay, NR: not reported; NS: not significant; h: hour

*Data obtained from the author

#Lv 2022: see Agarwal A, Lamontagne F, Adhikari NKJ. Unclear reporting of secondary outcome in randomized trial of Lv et al. Ir J Med Sci 2021. DOI: 10.1007/s11845-021-02810-0.

Note:

i) Ramzkon 2011 (mortality): for high- (7/26) vs low-dose (7/23) subgroup mortality analysis, the control group (8/27) is divided into two (4/14 vs 4/13)

ii) Fowler 2014 (mortality): for high- (4/8) vs low-dose (3/8) subgroup mortality analysis, the control group (5/8) is divided into two (3/4 vs 2/4)

iii) Chen 2019 (mortality): for high- (4/39) vs low-dose (10/41) subgroup mortality analysis, the control group (14/42) is divided into two (7/20 vs 7/22)

Ref for this method: Cochrane handbook 23.3.4 (https://training.cochrane.org/handbook/current/chapter-23#section-23-3-4)

## Table S6: Summary of Adverse Events

| **No** | **Author, year (country)** | **Definition of adverse events** | **Vitamin C Group 1** | **Vitamin C Group 2** | **Control** |
| --- | --- | --- | --- | --- | --- |
| 1 | Ferron-Celma 2008 (Spain) | NR | NR | N/A | N/R |
| 2 | **Razmkon 2011 (Iran)** | NR | 0/26 | 0/23 | 0/27 |
| 3 | **Fowler 2014 (USA)** | Hypotension, tachycardia, hypernatremia, or nausea/vomiting | 0/8 | 0/8 | 0/8 |
| 4 | Zabet 2016 (Iran) | Nausea, vomiting, abdominal pain, hematuria,flushing, and significant arterial blood pressure change | 0/14 | N/A | 0/14 |
| 5 | **Chen 2019 (China)** | Nausea/vomiting, abdominal pain, hematuria, significant blood glucose abnormality, significant change in arterial blood pressure | 0/41 | 0/39 | 0/42 |
| 6 | Fowler 2019  (USA) | NR | 0/84 | N/A | 0/83 |
| 7 | Niu 2019 (China) | NR | NR | N/A | NR |
| 8 | Lv 2020 (China) | NR | NR | N/A | NR |
| 9 | Jamali Moghadam Siahkali 2021 (Iran) | Headache, nausea, bloating, abdominal discmfort | 0/30 | N/A | 0/30 |
| 10 | Kassem 2021  (Egypt) | Hypernatremia  AKI | 3/40  4/40 | N/A | 1/40; p=0.615  5/40; p=1.000 |
| 11 | Mahmoodpoor 2021 (Iran) | Hypotension, tachycardia, nausea/vomiting, hypernatremia | 3/40 | N/A | 0/40 |
| 12 | Zhang 2021 (China) | Nausea/vomiting, electrolyte disturbance, AKI, | 0/27 | N/A | 0/29 |
| 13 | Ap 2022 (India) | NR | NR | N/A | NR |
| 14 | Rosengrave 2022 (New Zealand) | Common Terminology Criteria for Adverse Events (CTCAE, version 4.0) | 0/20 | N/A | 1/20 (Gastrointestinal bleeding) |
| 15 | Wacker 2022 (USA) | See Table S9 for all reported adverse events  Possible related to study drug | 15/60  1/60 (nausea) | N/A | 12/64  2/64 (bradycardia, loose stools) |
| 16 | Lamontagne 2022 (International) | AKI stage 3  Acute hemolysis  Hypoglycemia  Serious adverse event | 162/429  0/429  26/429  1/429 (anaphylatic reaction)  Total: 189/429 | N/A | 164/433  0/433  22/433  0/433  Total: 186/433 |

AKI: acute kidney injury, N/A: not applicable, NR: not reported

## Table S7: Trial sequential analysis for overall mortality (sensitivity analysis)

**Biggerstaff-Tweedie Random-effects models, Alpha 5%, beta 10%**

| **Relative Risk reduction, %** | **Control group event rate, %** | **I^2^,**  **%** | **D^2^,**  **%** | **RIS, n** | **RIS achieved,**  **%** | **TSA adjusted RR (95% CI)** | **Results** |
| --- | --- | --- | --- | --- | --- | --- | --- |
| **Overall analysis (16 studies; n=2130), Biggerstaff-Tweedie Random effect model** | | | | | | | |
| 30 | 35 | 42 | 49 | 1565 | - | 0.78 (0.75-0.80) | Sig risk reduction |
| 25 | 35 | 42 | 49 | 2292 | 92.9 | 0.78 (0.76-0.80) | Sig risk reduction |
| 20 | 35 | 42 | 49 | 3636 | 58.6 | 0.78 (0.75-0.80) | Sig risk reduction |
| **Subgroup with lower control group mortality (8 studies; n=1337), Biggerstaff-Tweedie Random effect model** | | | | | | | |
| 30 | 30 | 18 | 38 | 1580 | 84.6 | 0.97 (0.93-1.02) | Futile |
| 25 | 30 | 18 | 38 | 2319 | 57.7 | 0.97 (0.92-1.03) | Futile |
| 20 | 30 | 18 | 38 | 3690 | 36.2 | 0.97 (0.90-1.04) | Uncertain |
| **Subgroup with higher control group mortality (8 studies; n=793), Biggerstaff-Tweedie Random effect model** | | | | | | | |
| 30 | 45 | 0 | 16 | 646 | - | 0.65 (0.64-0.66) | Sig risk reduction |
| 25 | 45 | 0 | 16 | 941 | 84.3 | 0.65 (0.64-0.67) | Sig risk reduction |
| 20 | 45 | 0 | 16 | 1484 | 53.4 | 0.65 (0.63-0.67) | Sig risk reduction |

RIS: required information size, TSA: Trial sequential analysis

## Table S8: Differences between protocol and review

|  | The following **secondary outcomes** are added: incidences of acute kidney injury and renal replacement therapy, SOFA score at 96-hour, dose and duration of vasopressor used, and the Incidences of adverse event (as reported by the original manuscript). |
| --- | --- |
|  | The following **subgroup analyses** are added: studies that enrolled patients above versus below the median overall control group mortality, > versus ≤ median CCN score, low versus other risk of bias, duration of treatment > or ≤ 4 days, commencement of the intervention ≤ versus >24h of an event, and bolus versus continuous infusion |
|  | **Sensitivity analysis** for the primary outcome using the fixed-effect model was added. |
|  | **Sensitivity analysis** for the primary outcome using the Biggerstaff-Tweedie Random-effects models was added for TSA. |
|  | The p-value used to trend towards significance (for hypothesis-generating purposes) is lowered from <0.20 to <0.10. |
|  | Risk of bias assessment using the Cochrane Risk of Bias version 2 (**ROB2**) was added on top of the Canadian Critical Care Nutrition Methodological Scoring System. |
|  | Evaluation of certainty of the evidence of the primary outcome using the Grading of Recommendations Assessment, Development, and Evaluation (**GRADE**) system was added. |
|  | Trial sequential analysis was added to further confirm the significant findings of our meta-analysis of primary outcomes and subgroup of studies that enrolled patients above versus below the median overall control group mortality:   - 1. The control group event rate (Pc) was defined based on the control group mortality rate of our current meta-analysis: 365/1028= 35.5%≈ 35%   2. Alpha is set at conventional value of 5% and beta is set at 10% (power 90%)   3. DerSimonian-Laird Random effect model   4. Heterogeneity was assessed by the diversity of the current analysis   5. Relative risk reduction (RRR) from previous meta-analysis.  In the IV Vitamin C monotherapy subgroup analysis: Agarwal et al (DOI: 10.1056/EVIDoa2200105) found an RR of 0.67 (95% CI 0.55-0.82) [See Figure S18 by Agarwal et al], while Patel et al (DOI: 10.1097/CCM.0000000000005320) found an RR of 0.64 (95% CI 0.49, 0.83). These are translated to a relative risk reduction of 33% (18%-45%) and 36% (17%-51%). Therefore, we chose an RRR of 30% based on these findings and conducted sensitivity analyses with RRR 25% and 20%. |

## Figure S1: PRISMA flowchart

**
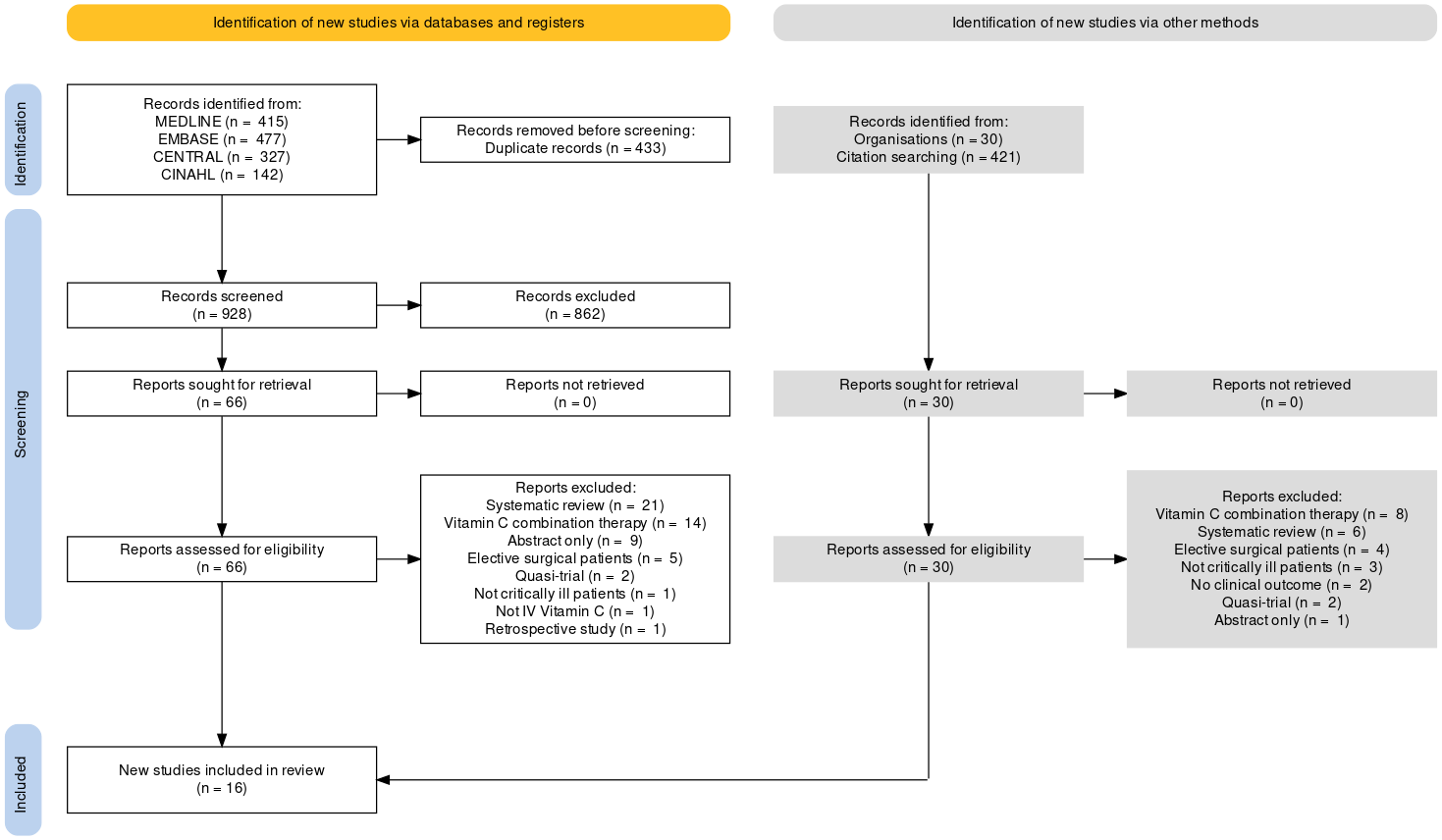
**

**Note: Organizations refer to personal files**

## Figure S2: Risk of bias 2 traffic light plot for Overall Mortality


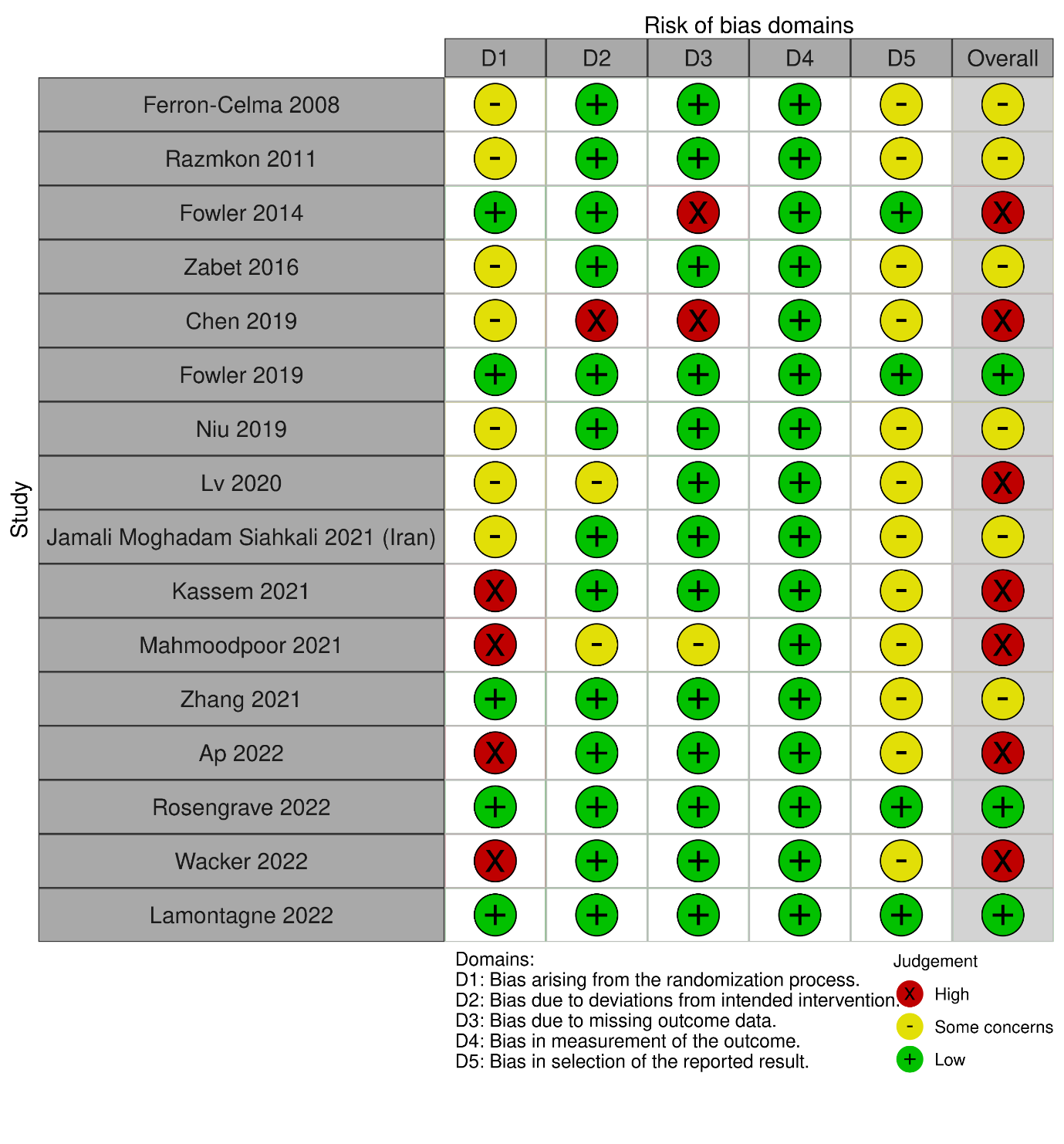


## Figure S3: Overall mortality (single vs multicenter trials)

**
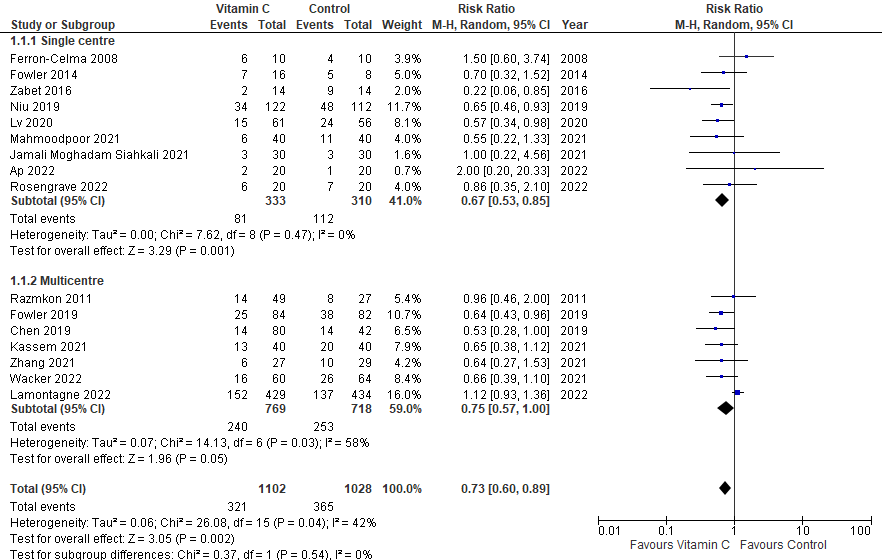
**

## Figure S4: Overall mortality (sepsis vs non-sepsis)


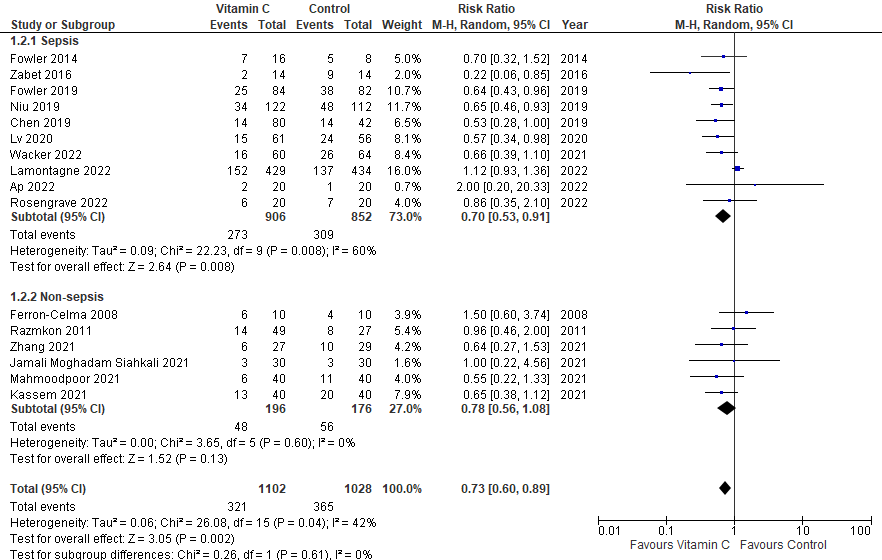


## Figure S5: Overall mortality (higher ≥10000 mg/day vs lower dose)


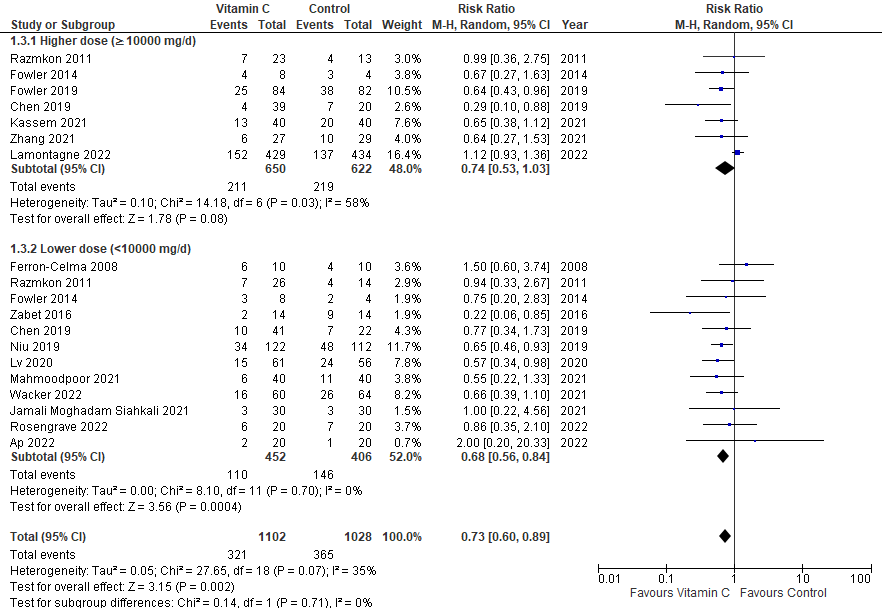


## Figure S6: Overall mortality (median control group mortality > vs ≤ 37.5%)

**
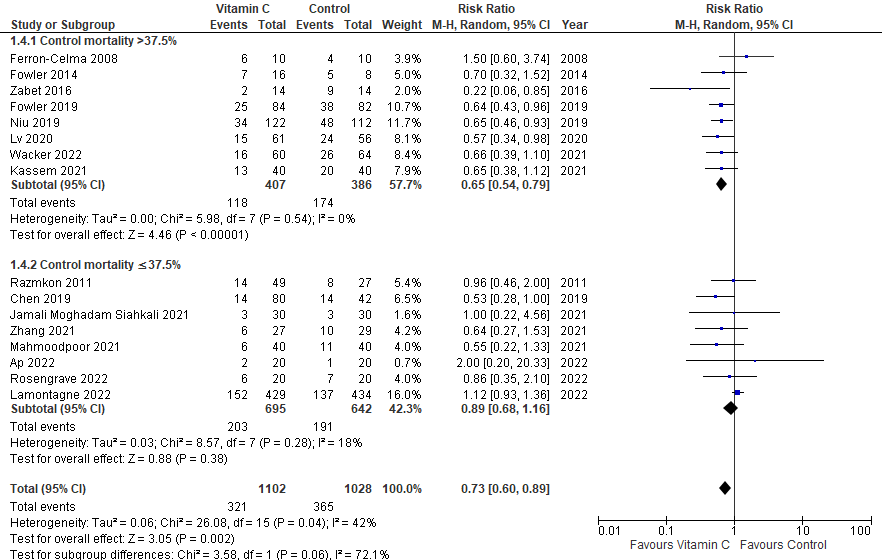
**

## Figure S7: Overall mortality (median CCN score >9 vs ≤9)


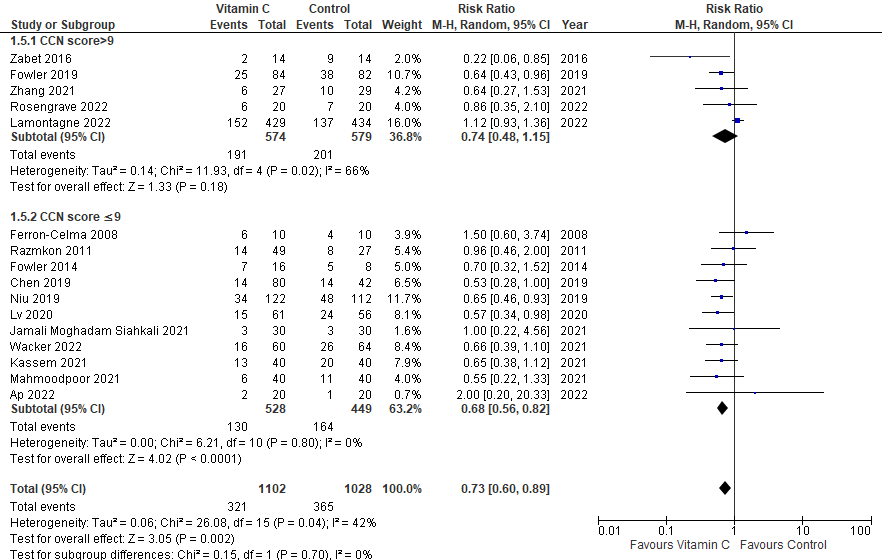


CCN: critical care nutrition

## Figure S8: Overall mortality (Low vs other risk of bias)

**
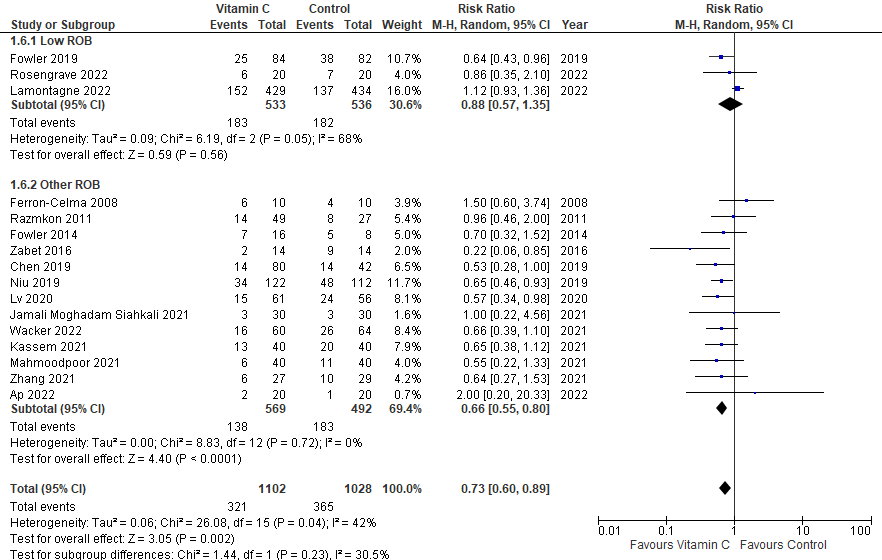
**

## Figure S9: Overall mortality (start of intervention ≤ vs >24h of ICU admission/septic shock/pressor initiation etc)

**
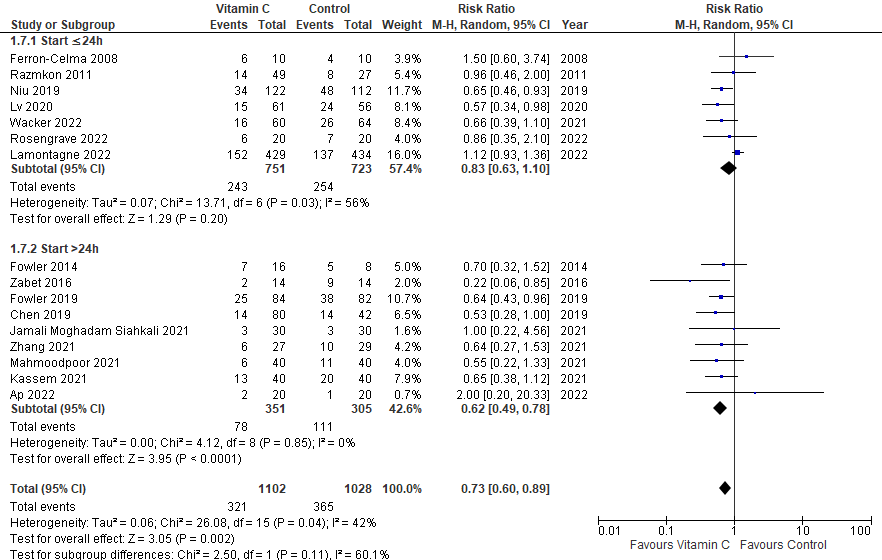
**

**Note: It is unclear when the intervention was initiated for Jamali Moghadam Siahkali 2021, Kassem 2021, Mahmoodpoor 2021, and Ap 2022, therefore they are assumed to be started >24 hours.**

Sensitivity analysis after excluding the above 4 studies showed a similar finding.

Start ≤24h: RR 0.83 (95% CI 0.63, 1.10); p=0.20; I^2^=56%; 7 studies

Start >24h: RR 0.59 (95% CI 0.45, 0.79); p=0.0004; I^2^ =0%; 5 studies

Overall: RR 0.73 (95% CI 0.58, 0.93); p=0.009; I^2^ =54%; 12 studies

Test for subgroup differences p=0.10; I^2^ =62.6%

## Figure S10: Overall mortality (Duration of treatment > vs ≤4 days)


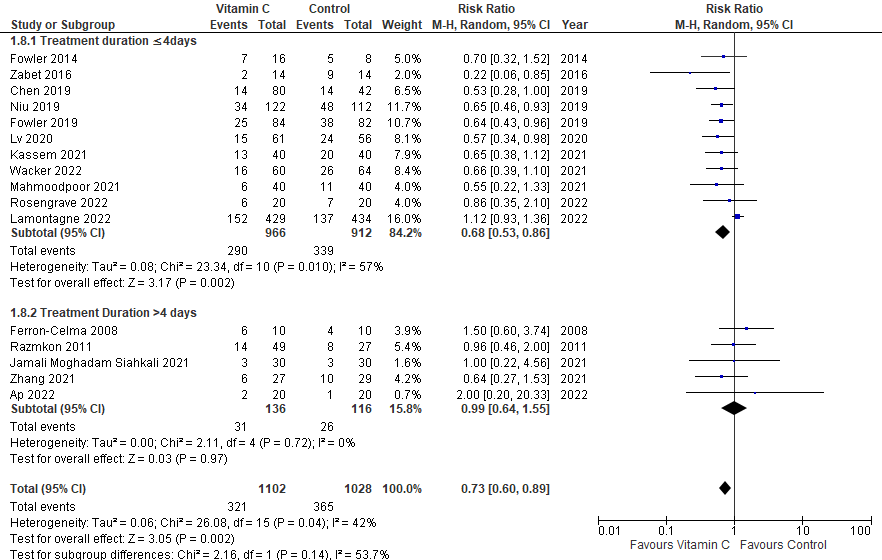


## Figure S11: Overall mortality (Bolus vs continuous infusion)

**
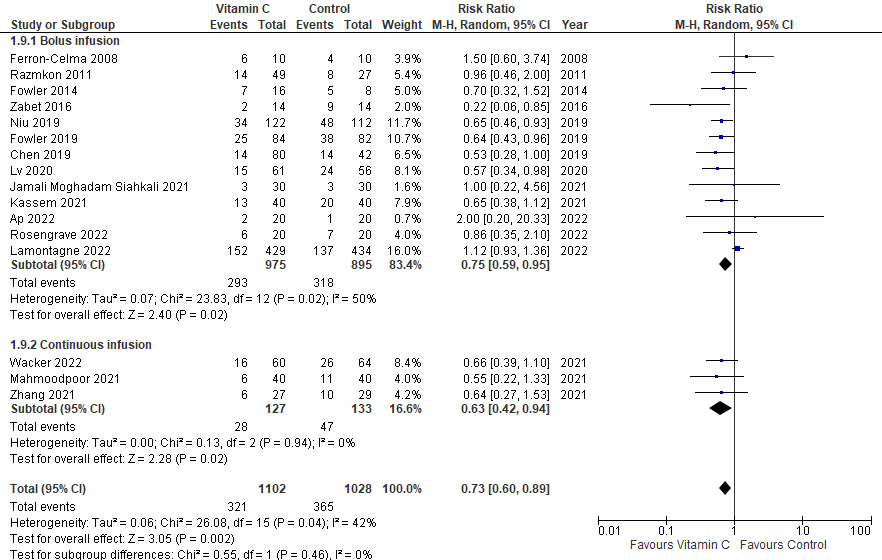
**

## Figure S12: Overall mortality: Sensitivity analysis: studies that measured and reported baseline vitamin C deficit

**
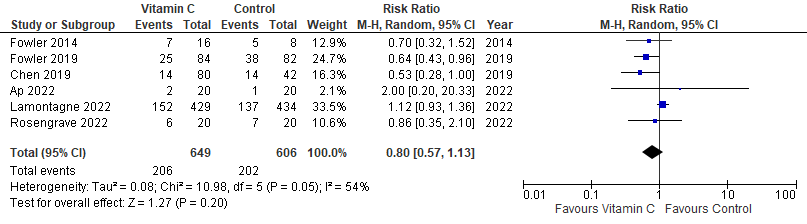
**

## Figure S13: 28-day mortality (single vs multicenter)


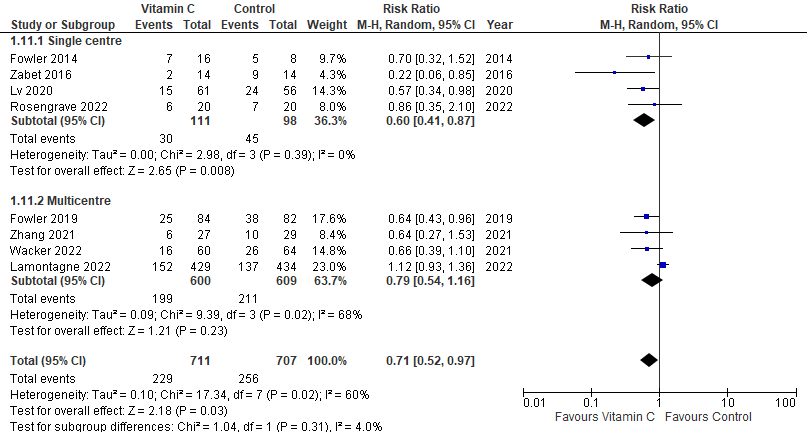


## Figure S14: 28-day mortality (sepsis vs non-sepsis)


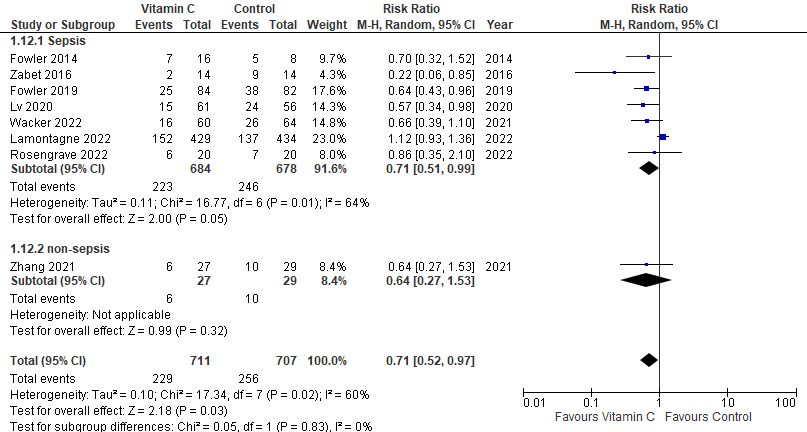


## Figure S15: 28-day mortality (higher dose ≥10000 mg/day vs lower dose)


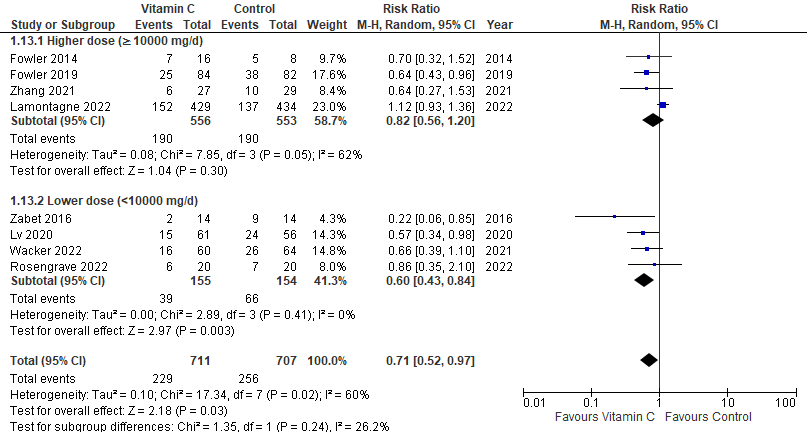


## Figure S16: 28-day mortality (median control group mortality > vs ≤ 37.5%)


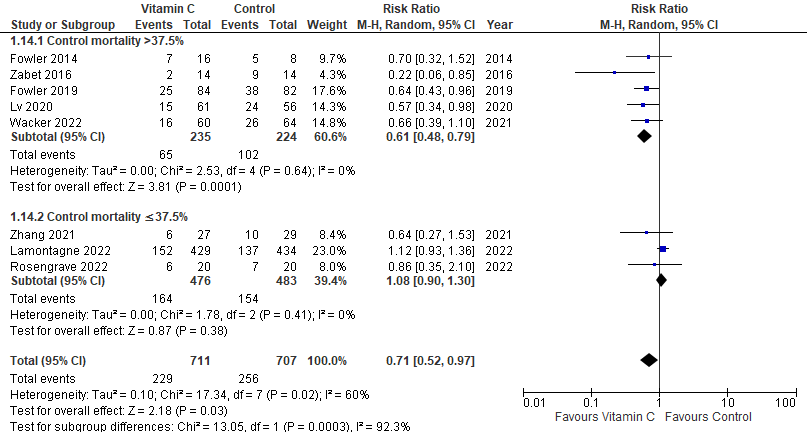


## Figure S17: 28-day mortality (median CCN score >9 vs ≤9)

**
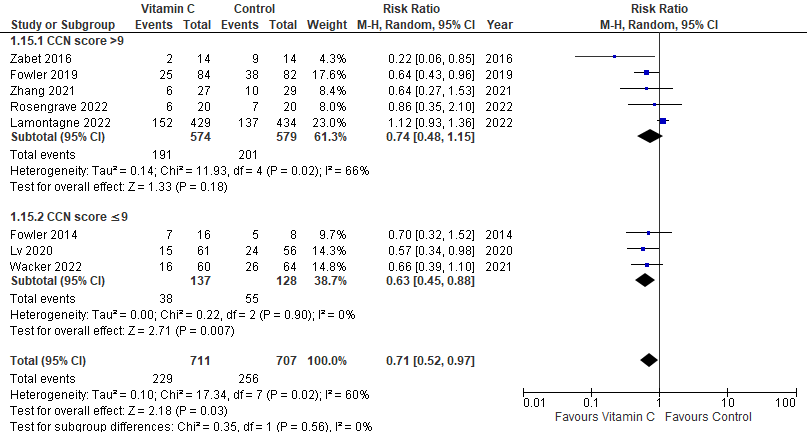
**

## Figure S18: 28-day mortality (low vs other risk of bias)

**
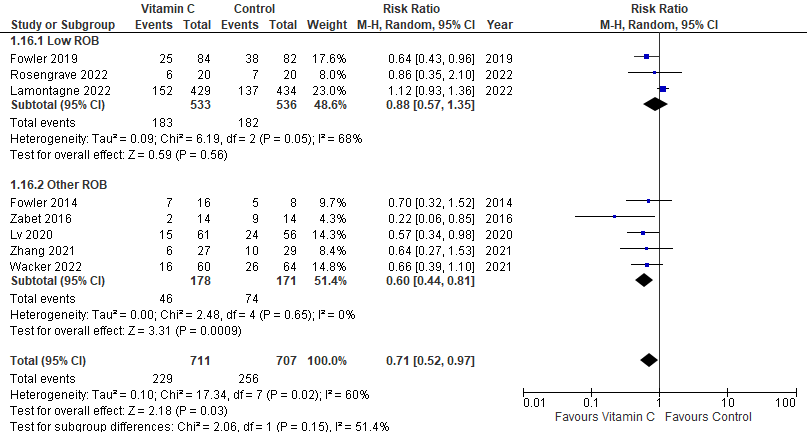
**

## Figure S19: 28-day mortality (start of intervention ≤ vs >24h of ICU admission/septic shock/pressor initiation etc.)

**
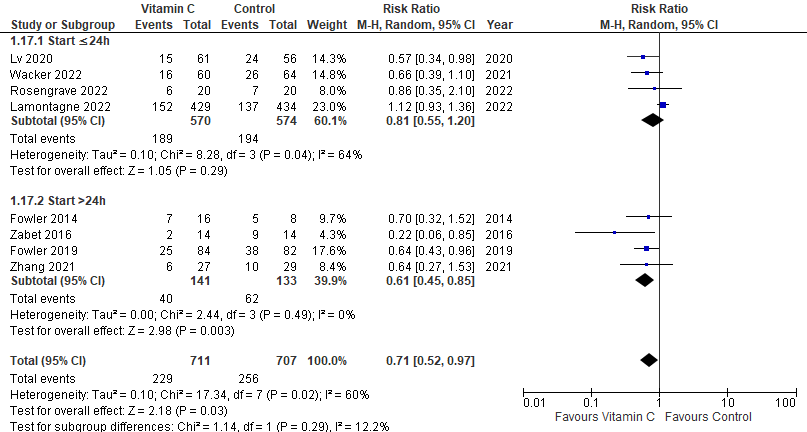
**

## Figure S20: 28-day mortality (Duration of treatment > vs ≤4 days)

**
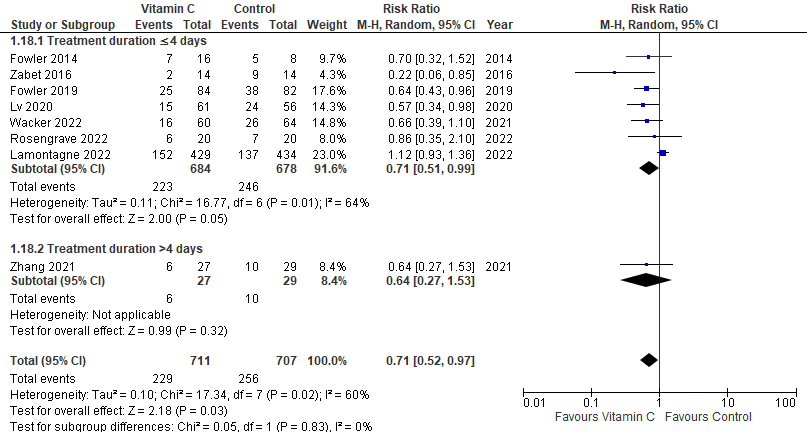
**

## Figure S21: 28-day mortality (Bolus vs continuous infusion)

**
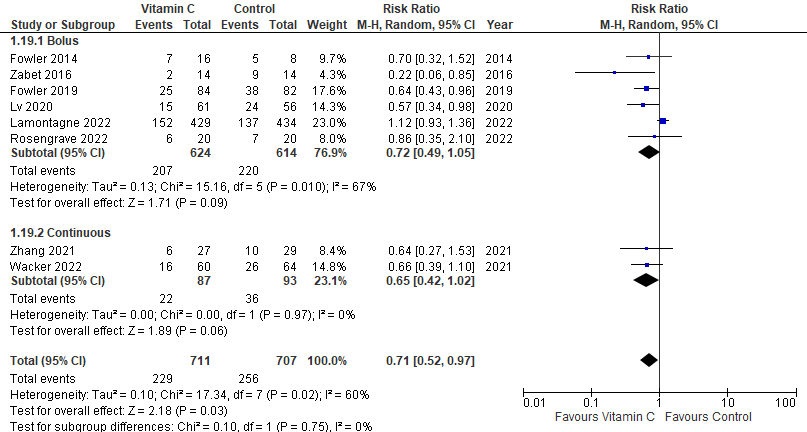
**

## Figure S22: Summary of subgroup analysis for 28-day mortality

**
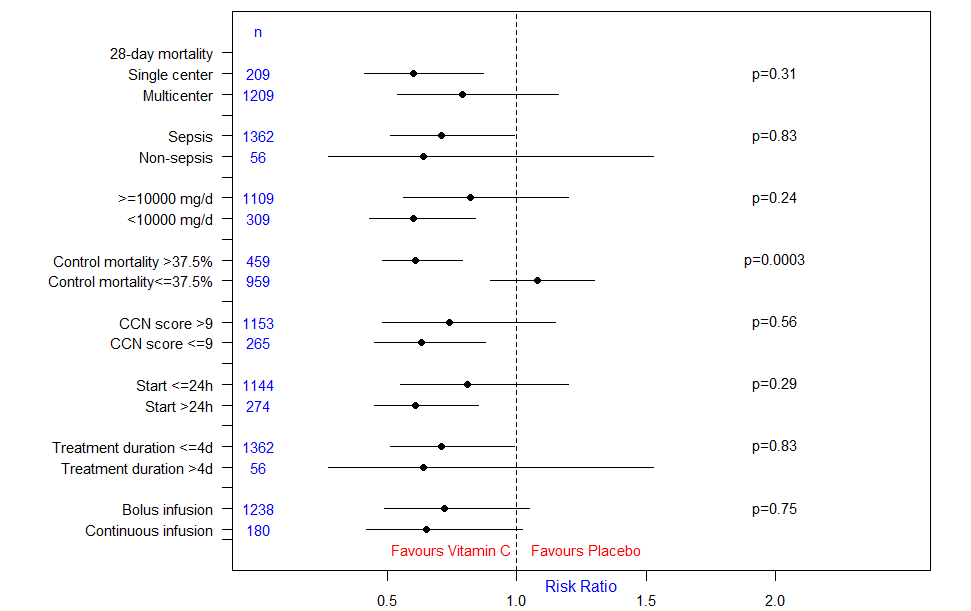
**

## Figure S23: Longer-term mortality (≥60 days and the longest follow-up reported)

**
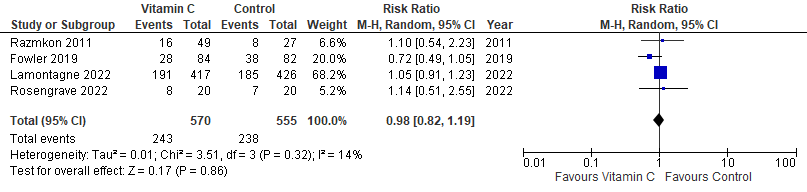
**

**Note:**

Razmkon 2011: 6-month, Fowler 2019: 60-day, Rosengrave: 90-day , Lamontagne 2022: 6-month

## Figure S24: Duration of mechanical ventilation


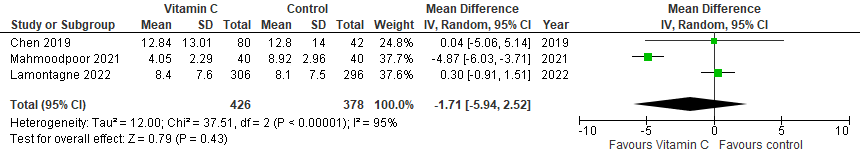


## Figure S25: ICU length of stay


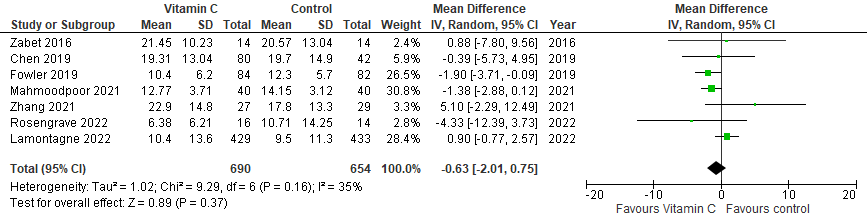


Figure S26: Hospital length of stay
**
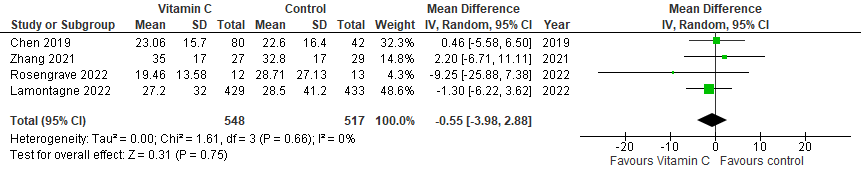
**

## Figure S27: Incidence of acute kidney injury

**
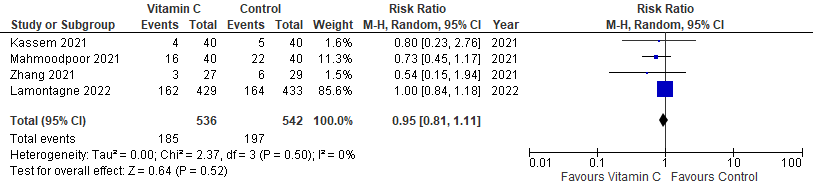
**

## Figure S28: Incidence of renal replacement therapy

**
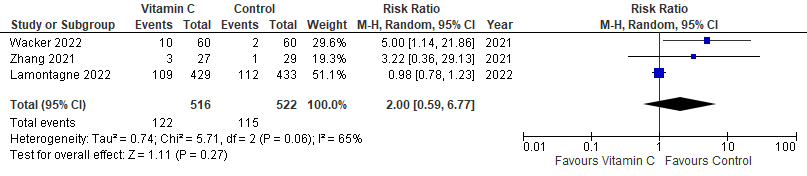
**

## Figure S29: Sequential organ failure assessment (SOFA) score at 96h

**
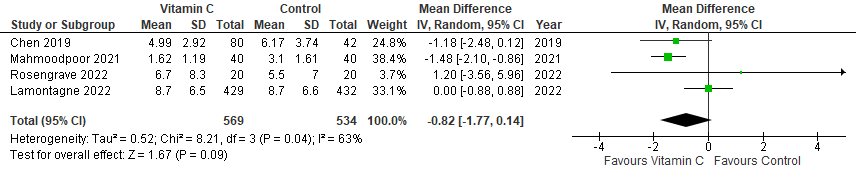
**

## Figure S30: Dose of vasopressors

**
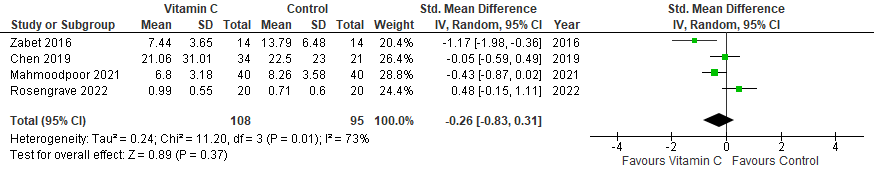
**

Zabet 2016: vasopressor usage in 72h in ug/min

Chen 2019: vasopressor usage in 96h in ug/min

Mahmoohpoor 2021: vasopressor dose in ug/min

Rosengrave 2022: mean vasopressor dose in units/min

## Figure S31: Days on vasopressors

**
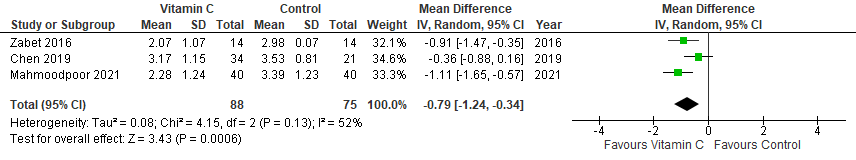
**

## Figure S32: (a) Adverse events

**
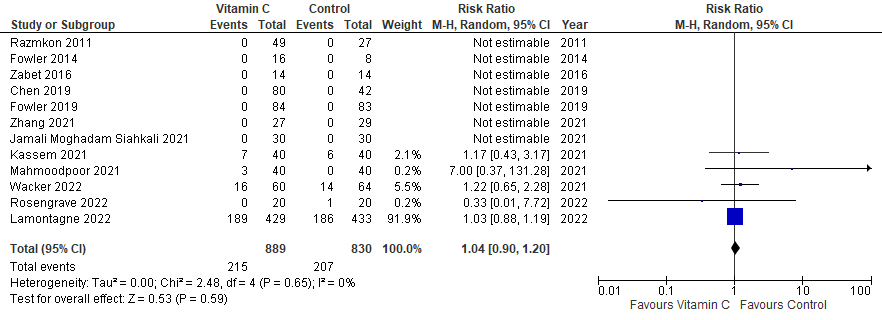
**

## Figure S32: (b) Adverse events (with continuity correction by adding 0.01 to cells with zero events)


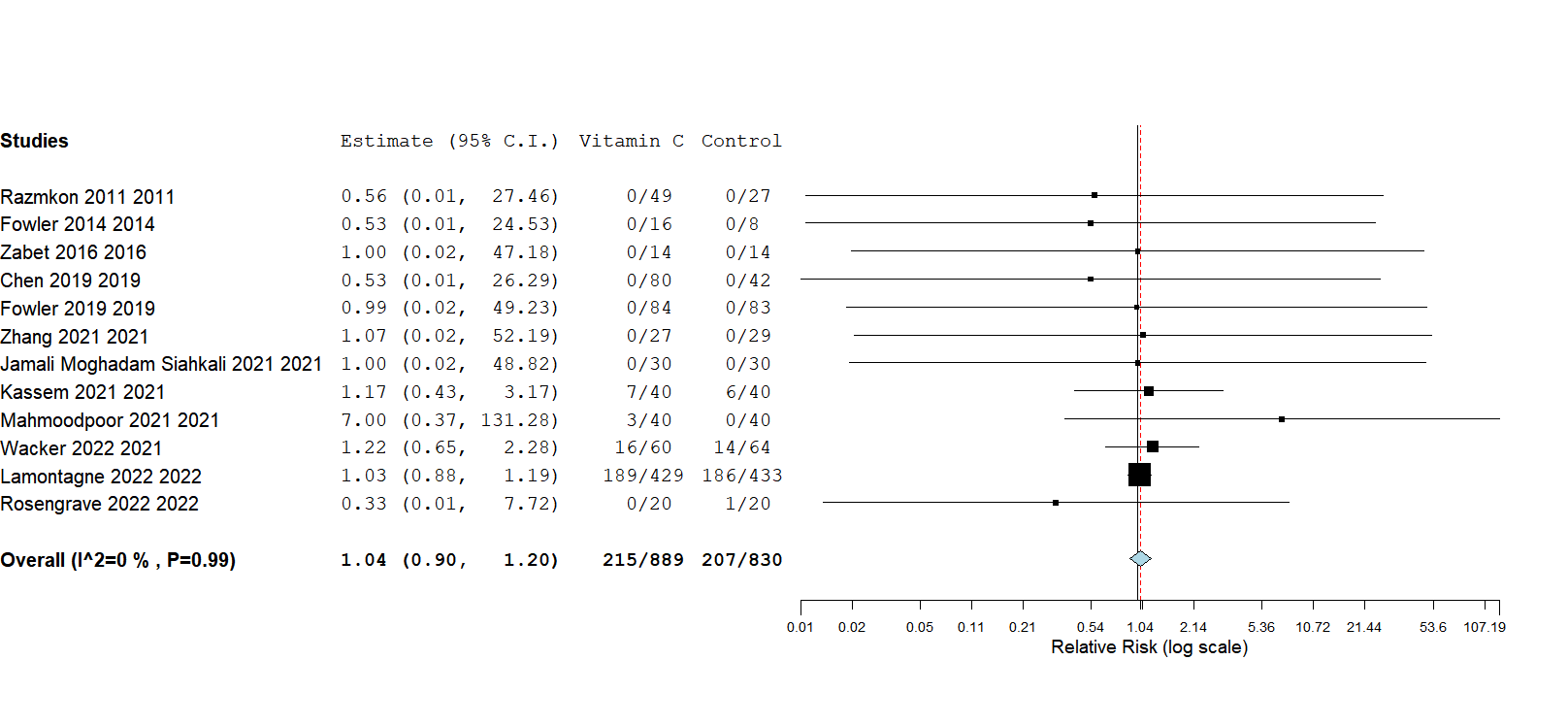


Note: this analysis is performed using Open Meta-Analyst software downloaded from http://www.cebm.brown.edu/openmeta/ [Accessed 15^th^ September 2022]

## Figure S33: Funnel plot for overall mortality

H0: beta1 = 0; no small-study effects, beta1 = -0.37, SE of beta1 = 0.598, z = -0.61, p-value = 0.5401

**
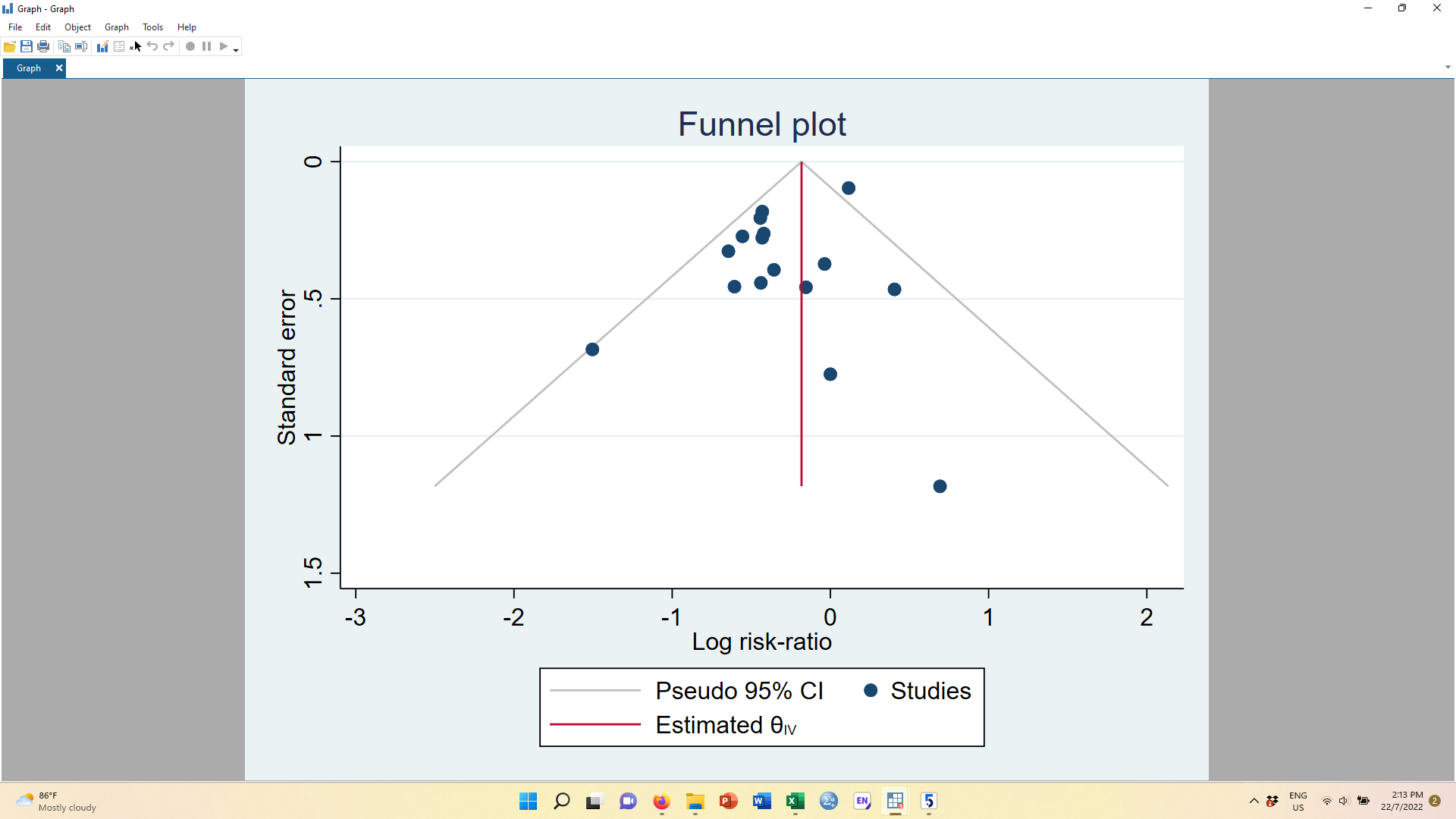
**

## Figure S34: TSA for Overall mortality - subgroup analysis in trials with below median control group mortality - Relative risk reduction 30%

**
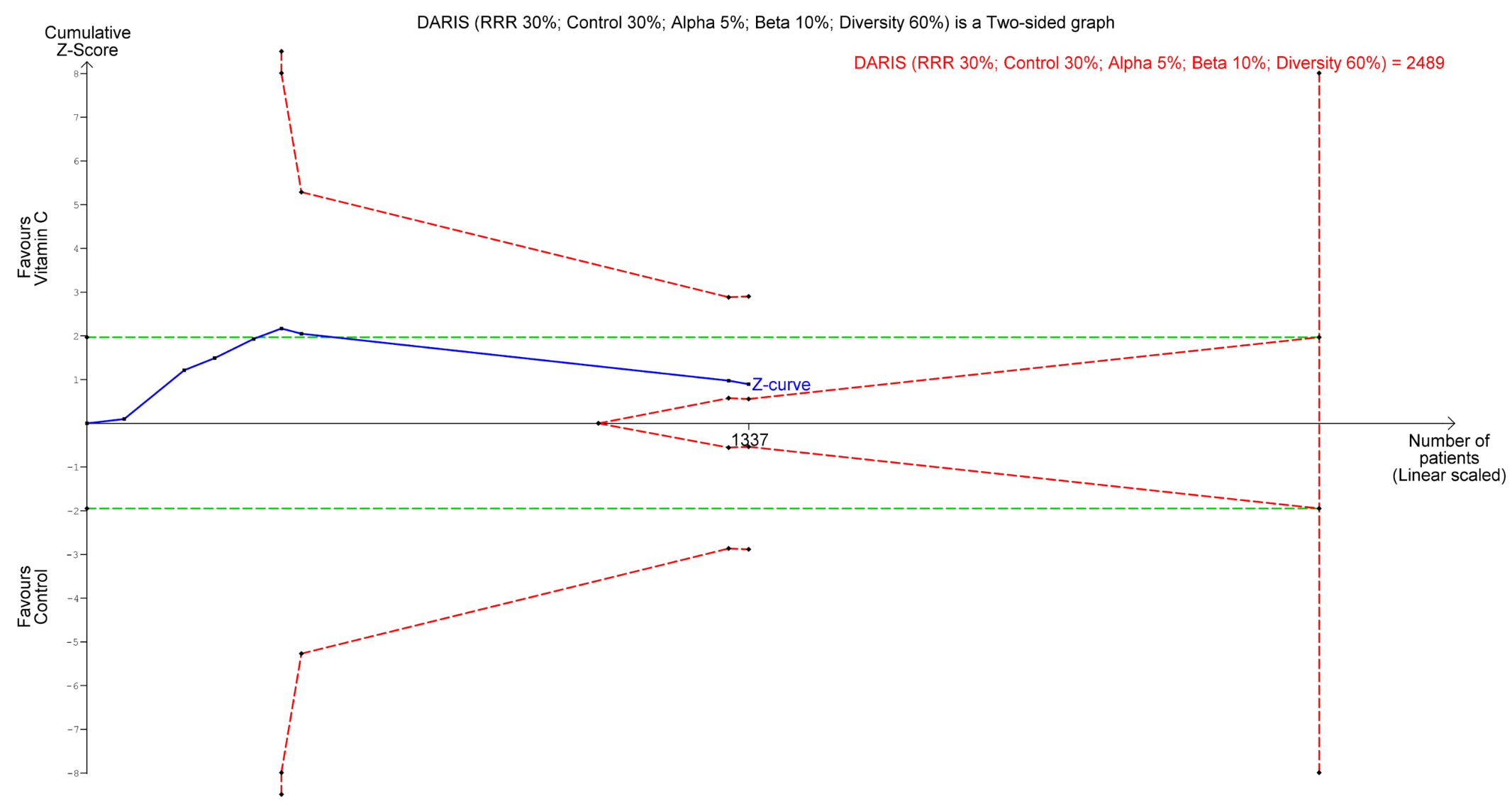
**

**DARIS: diversity-adjusted required information size; RRR: relative risk reduction**

## Figure S35: TSA for Overall mortality - subgroup analysis in trials below median control group mortality - Relative risk reduction 25%

**
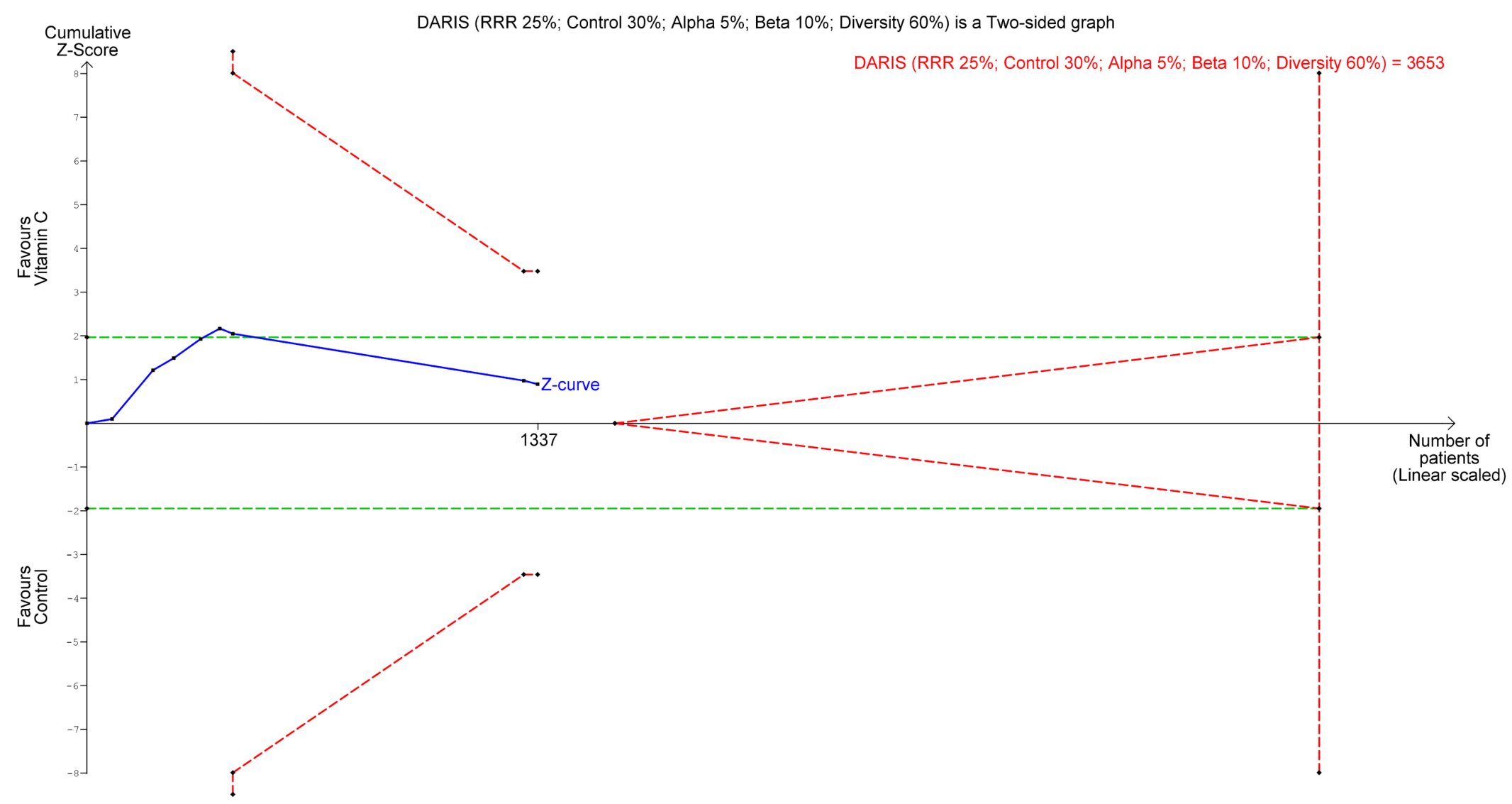
**

## Figure S36: TSA for Overall mortality - subgroup analysis in trials below median control group mortality (≤37.5%) - Relative risk reduction 20%

**
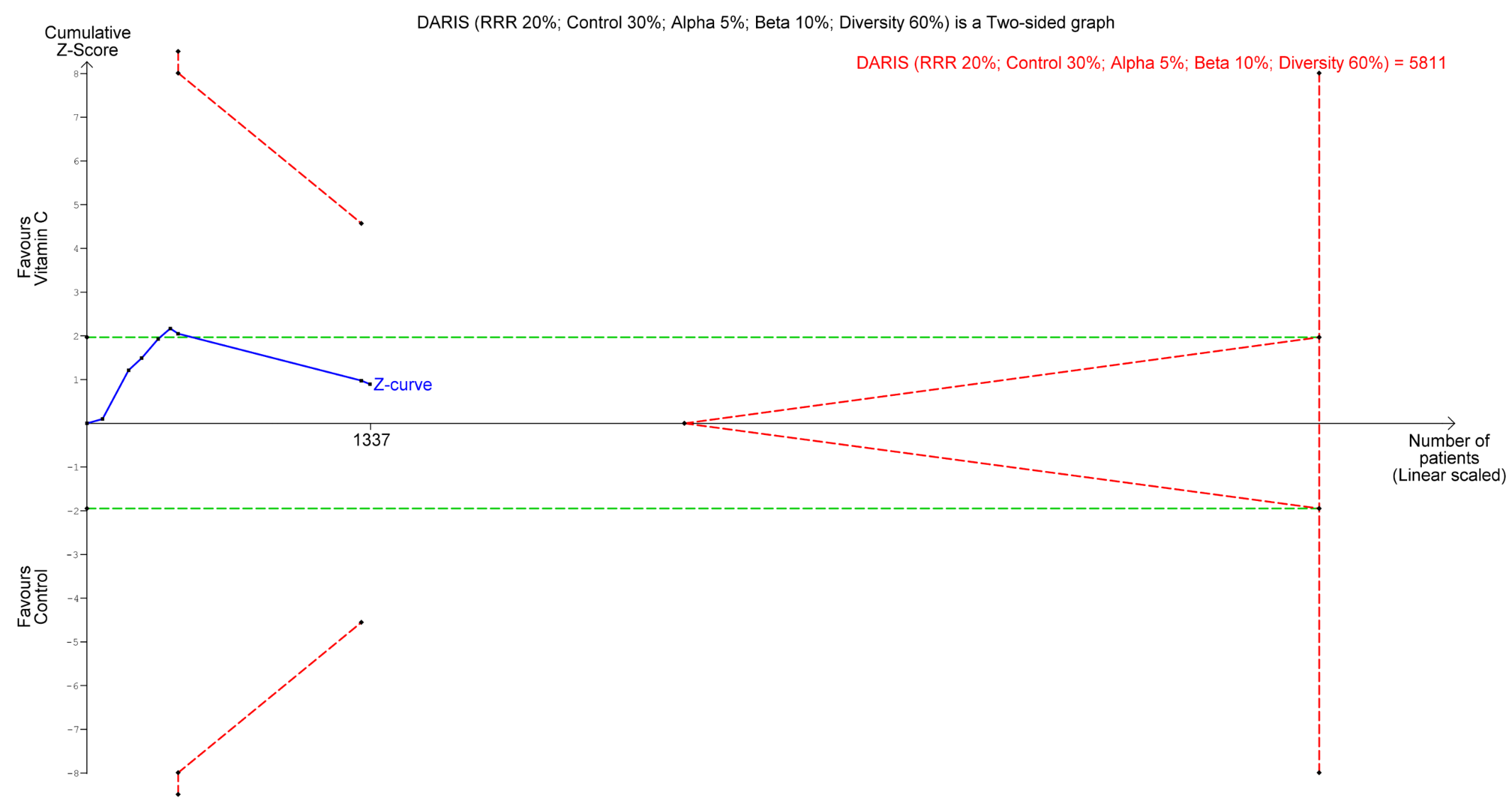
**

## Figure S37: TSA for Overall mortality - subgroup analysis in trials above (>37.5%) median control group mortality - Relative risk reduction 30%

**
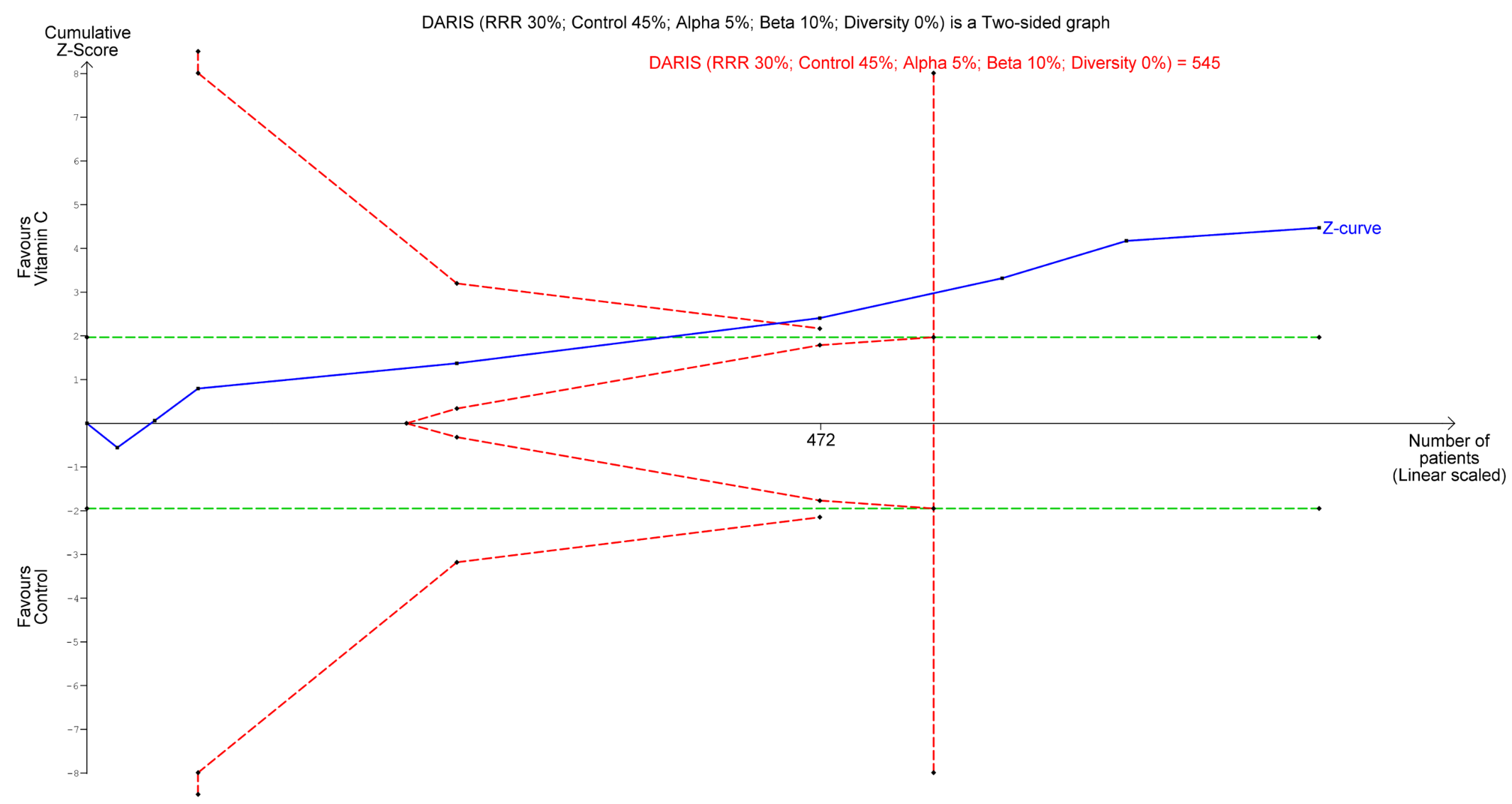
**

## Figure S38: TSA for Overall mortality - subgroup analysis in trials above (>37.5%) median control group mortality - Relative risk reduction 25%

**
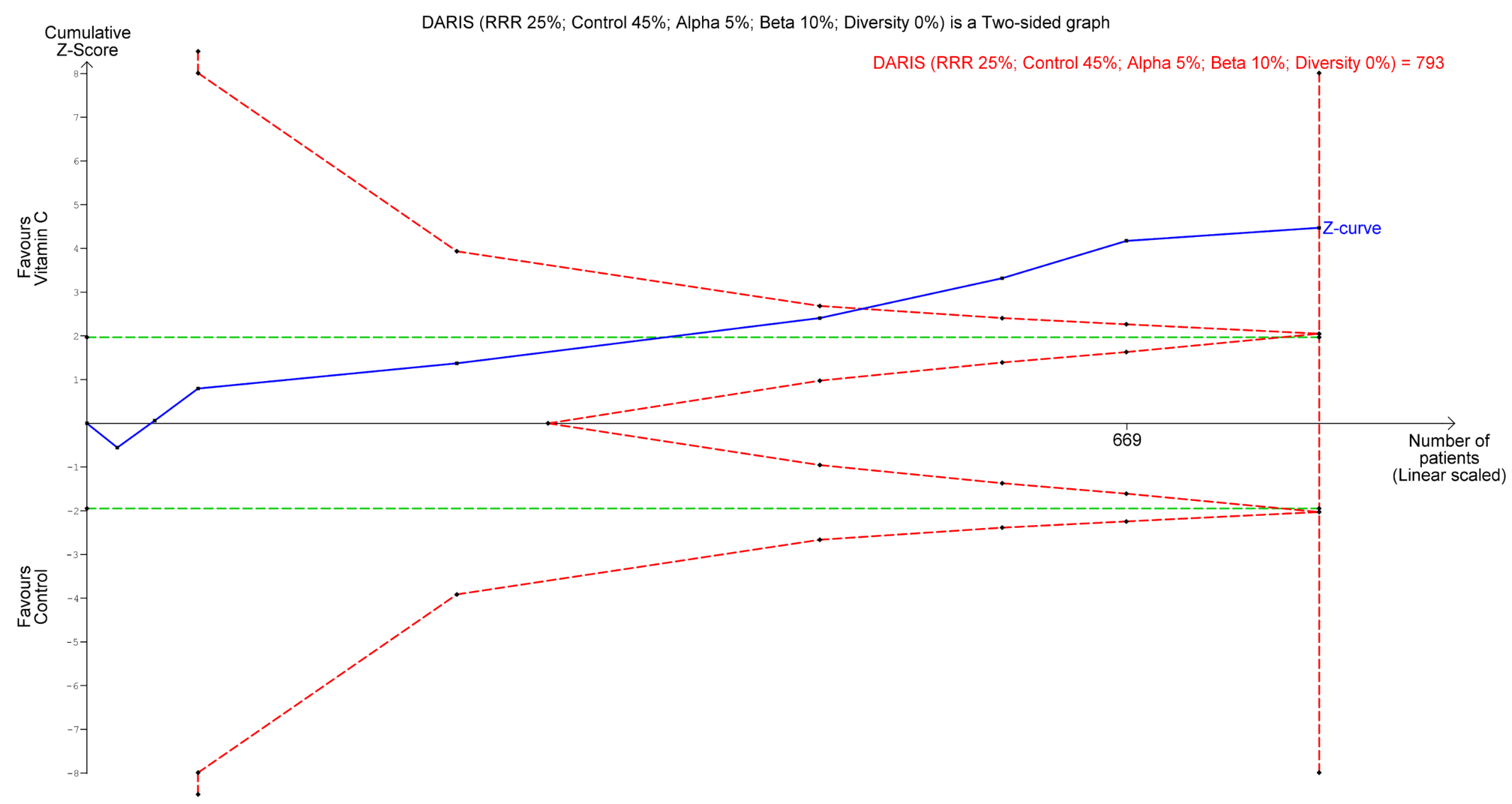
**

## Figure S39: TSA for Overall mortality - subgroup analysis in trials above (>37.5%) median control group mortality - Relative risk reduction 20%


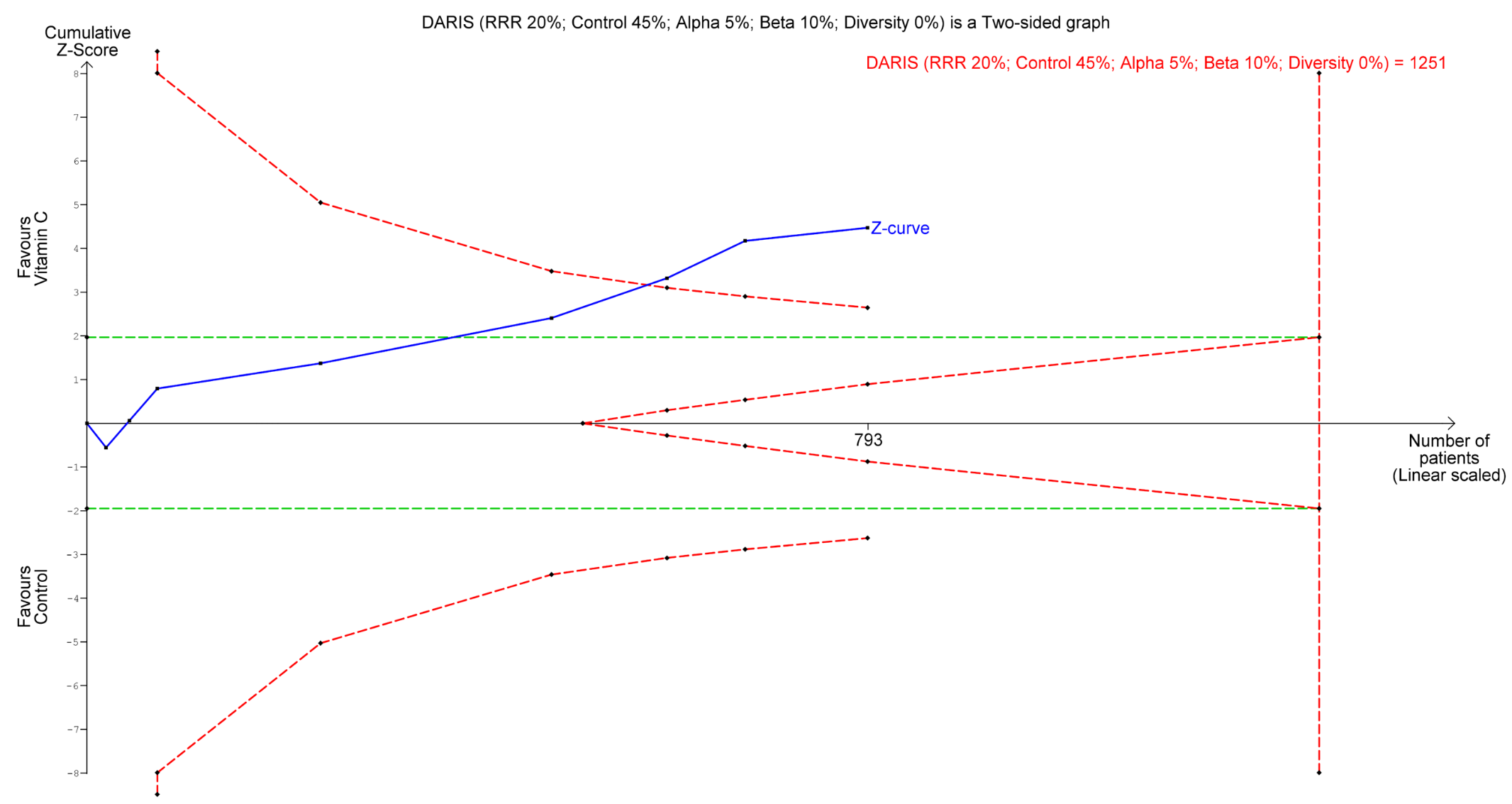

Supplement: Supplementary file 1 — Additional file 1: Methodology. PRISMA 2020 checklist. Results. Table S1 Search strategy. Table S2 List of excluded studies. Table S3 Critical care nutrition methodological system. Table S4 Intervention. Table S5 Outcomes summary. Table S6 Summary of adverse events. Table S7 Trial sequential analysis for overall mortality (sensitivity analysis). Table S8 Differences between protocol and review. Figure S1 PRISMA flowchart. Figure S2 Risk of bias 2 traffic light plot. Figure S3 Overall mortality (single vs multicenter trials). Figure S4 Overall mortality (sepsis vs non-sepsis). Figure S5 Overall mortality (higher ≥10000 mg/day vs lower dose). Figure S6 Overall mortality (median control group mortality > vs ≤ 37.5%). Figure S7 Overall mortality (median CCN score >9 vs ≤9). Figure S8 Overall mortality (low vs other risk of bias). Figure S9 Overall mortality (start of intervention ≤ vs >24 h of ICU admission/septic shock/pressor initiation etc.). Figure S10 Overall mortality (duration of treatment > vs ≤4 days). Figure S11 Overall mortality (bolus vs continuous infusion). Figure S12 Overall mortality sensitivity analysis studies that measured and reported baseline vitamin C deficit. Figure S13 28-day mortality (single vs multicenter). Figure S14 28-day mortality (sepsis vs non-sepsis). Figure S15 28-day mortality (higher dose ≥10000 mg/day vs lower dose). Figure S16 28-day mortality (median control group mortality > vs ≤ 37.5%). Figure S17 28-day mortality (median CCN score >9 vs ≤9). Figure S18 28-day mortality (low vs other risk of bias). Figure S19 28-day mortality (start of intervention ≤ vs >24h of ICU admission/septic shock/pressor initiation etc.). Figure S20 28-day mortality (Duration of treatment > vs ≤4 days). Figure S21 28-day mortality (bolus vs continuous infusion). Figure S22 Summary of subgroup analysis for 28-day mortality. Figure S23 Longer term mortality (≥60 days). Figure S24 Duration of mechanical ventilation. Figure S25 ICU length of stay. Figure S26 [file 13613_2023_1116_MOESM1_ESM.docx]
